# Comparative Phylogeography of West African Rainforest Frogs Reveals Regional Variation in Refugia Dynamics

**DOI:** 10.1111/mec.70043

**Published:** 2025-07-30

**Authors:** Mario Ernst, Daniel M. Portik, Gabriel H. Segniagbeto, Caleb Ofori‐Boateng, Joseph Doumbia, Johannes Penner, N'Goran G. Kouamé, Matthew K. Fujita, Adam D. Leaché, Mozes P. K. Blom, Mark‐Oliver Rödel

**Affiliations:** ^1^ Museum für Naturkunde – Leibniz Institute for Evolution and Biodiversity Science Berlin Germany; ^2^ Institut für Biologie Humboldt – Universität zu Berlin Berlin Germany; ^3^ California Academy of Sciences San Francisco California USA; ^4^ Laboratoire d'Ecologie et d'Ecotoxicologie, Faculté des Sciences Université de Lomé Lomé Togo; ^5^ CSIR‐Forestry Research Institute of Ghana, KNUST Kumasi Ghana; ^6^ Security Africa Mining and Environmental Consulting (SAMEC), Kipé T2 Conakry République de Guinée; ^7^ Frogs & Friends e.V. Berlin Germany; ^8^ Laboratoire de Biodiversité et Ecologie Tropicale, UFR Environnement Université Jean Lorougnon Guédé Daloa Côte d'Ivoire; ^9^ The University of Texas at Arlington Arlington Texas USA; ^10^ Department of Biology and Burke Museum of Natural History and Culture University of Washington Seattle Washington USA

**Keywords:** amphibians, conservation, ecology, genomics, phylogeography, population genetics

## Abstract

Understanding how species responded to climatic change in the past can help predict the long‐term implications of contemporary climate change. The Upper Guinean rainforests of West Africa are a global biodiversity hotspot, and it is well documented that climatic fluctuations in the Pleistocene drove the expansion and contraction of rainforest cover in this region. West African slippery frogs, genus *Conraua*, are rainforest specialists and present an excellent opportunity to study the effects of climate‐driven landscape changes on contemporary phylogeographic patterns and population dynamics. They exclusively inhabit rainforest streams and show little morphological or ecological disparity. We generated a population‐level dataset of genome‐wide restriction site‐associated DNA (RAD) loci for four species spread across Upper Guinea. The observed phylogeographic structure is consistent with previously postulated macro‐ and micro‐refugia. Building on these results, we used demographic modelling to trace demographic trends over time and infer population connectivity patterns. Overall, populations in topographically complex regions, like the Fouta Djallon, showed signatures of long‐term local persistence and milder changes in population size. In contrast, we found more dynamic histories of contraction and expansion in the main Upper Guinean rainforest block. Our findings provide insights into regional biodiversity patterns and show large variation in population responses to climatic fluctuations. This suggests that local environmental factors have played a key role in shaping population dynamics. Such insights are particularly relevant in relatively understudied biodiversity hotspots, such as the Upper Guinean rainforests of West Africa. Our results have implications for conservation management and prioritisation at the species, site, and ecosystem level.

## 1 Introduction

Climate refugia are important to conservation because of their potential to buffer climatic changes and support the persistence of species throughout periods of shifting climatic conditions, such as Pleistocene glaciations (Keppel et al. 2012; Willis et al. 2004). These glaciations had a major impact on the distribution of biodiversity in higher latitudes but simultaneously led to radical changes in temperature and precipitation in the tropics (Metcalfe and Nash 2012; Chiang 2009). At lower latitudes, such as tropical Africa, the climate became drier and cooler during glacial times, leading to the fragmentation and contraction of rainforests (Corlett and Primack 2011; Maley 1996). Climate refugia played an important role in supporting the persistence of ancient lineages throughout periods of climatic adversity and also in driving allopatric speciation (Maley 1996; Haffer 1969). However, the population dynamics associated with refugia can vary and depend not only on the biological properties of species but also on the geographic context (Bennett and Provan 2008; Hewitt 1996). Often, refugia are associated with areas that are topographically complex (Ashcroft et al. 2012; Dobrowski 2011). Topographically complex areas are landscapes with considerable variation in elevation and slope, including features such as steep mountains or deep valleys. Such areas offer diverse microenvironments and can allow species to persist locally (Byrne et al. 2022; Keppel et al. 2012). Whether or not populations persisting in refugia expand after periods of climatic adversity will depend on different factors, such as the dispersal capabilities of species and the presence of physical barriers. If the surrounding areas are dominated by permeable homogeneous landscapes, species will be more likely to expand than if the surrounding terrain is rugged. Topographically complex areas thus often show evidence of long‐term population persistence and isolation (Byrne et al. 2022; Moussalli et al. 2009). In contrast, regions characterised by more subdued or flatter landscapes are more frequently associated with demographic signatures of population contraction and expansion (Byrne et al. 2022; Byrne 2008). Identifying the locations where species have persisted and understanding the associated population dynamics is crucial in order to maximise ecosystem resilience against future climate change (Morelli et al. 2020; Hampe and Petit 2005).

**FIGURE 1:**
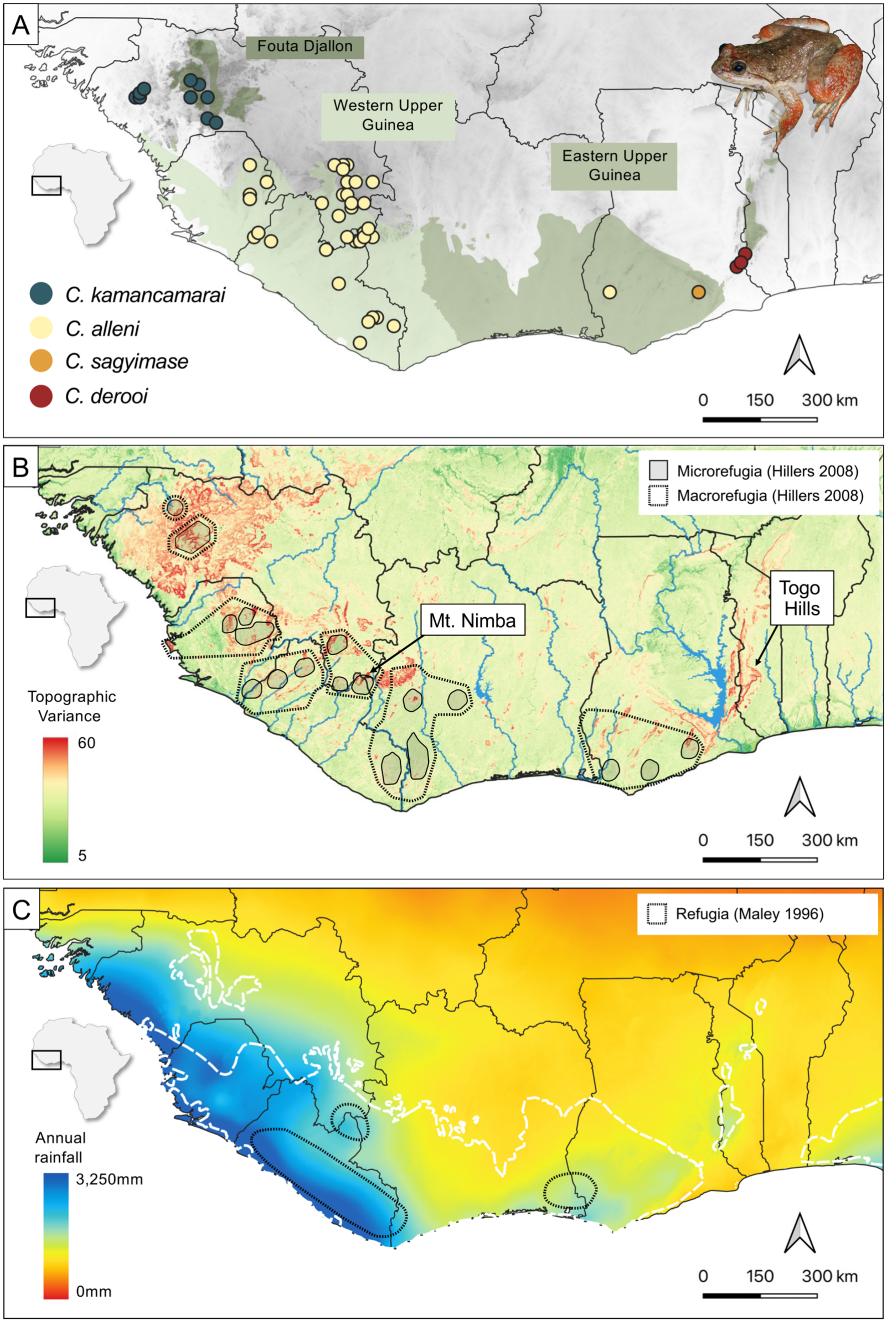
(A) Sampling localities of the four West African *Conraua* species (*Conraua kamancamarai*, *Conraua alleni*, *Conraua sagyimase*, and *Conraua derooi*). Green shades show the distribution of the Upper Guinea rainforest (modified from Olson et al. 2001). Upper right shows a picture of *Conraua derooi*. (B) Map showing topographic variance and major rivers across Upper Guinea. The variance was calculated in 9 × 9 pixel windows by taking the difference between the maximum and minimum elevation. This value was then logarithmically transformed and scaled by 10 for enhanced visualisation. Higher values (red) represent topographically complex terrain, such as steep slopes or deep valleys, while lower values (green) indicate flatter, more uniform landscapes. (C) Average annual sum of precipitation for the years 1970–2000 (Fick and Hijmans 2017). Legend with colour‐shading in the lower left in millimetres. West African forests delimitation in white contour.

The Upper Guinean rainforests are a global biodiversity hotspot (Myers et al. 2000). The region is expected to experience increasing temperatures and shifting precipitation patterns, which could trigger forest die‐off (Critical Ecosystem Partnership Fund (CEPF) 2022; Trisos et al. 2022). In particular, countries like Ghana, Togo, Benin, and Côte d'Ivoire are expected to receive decreased levels of mean annual rainfall (Ndehedehe et al. 2022), resembling changes in precipitation that have happened during previous climatic oscillations (Ernst et al. 2025; Wittig et al. 2007). Identifying areas that can buffer against such changes (i.e., climate refugia) is therefore important for conservation. Several refugia have been proposed for the last glacial maximum based on pollen and species distribution data (Ernst et al. 2025; Maley 1996; Figure 1c). However, comparative phylogeographic studies reveal a finer structure pointing at additional refugia (Hillers 2008; Nicolas et al. 2008). Hillers (2008) proposed a “refugia‐within‐refugia” scenario (Gómez and Lunt 2007) and identified larger refuge zones (macrorefugia) based on congruent phylogeographic signals across multiple species. Nested within these macrorefugia, Hillers (2008) also identified smaller refugial sites (microrefugia) harbouring independent evolutionary lineages (Ernst et al. 2025). These have been important for the persistence of specific species, thus constituting partial refugia (*sensu* Van Rompaey 1996). The Upper Guinean region has a diverse landscape with extensive lowlands as well as topographically complex regions, and both landforms contain putative refugia (Hardy et al. 2013; Hillers 2008). This region thus offers an ideal natural laboratory for testing how landscape context influences refugia dynamics. Many studies have investigated phylogeographic signals of past fragmentation in West Africa (e.g., Leaché et al. 2020; reviewed in Hardy et al. 2013), but fewer have explored the demographic signatures of refugia (Leaché et al. 2019; Hillers 2008; Nicolas et al. 2008). Furthermore, most studies focused on Lower Guinea and Central Africa (e.g., Helmstetter et al. 2020; Portik et al. 2017). West and Central African rainforests differ markedly in their biodiversity, which is being shaped by characteristic topographic, geologic, and climatic factors (Penner et al. 2011; Poorter et al. 2004). Therefore, the study of rainforest refugia and their role in shaping population dynamics requires a region‐specific approach (Billick and Price 2011). Furthermore, in order to infer how rainforests have responded to climatic fluctuations based on phylogeography, study systems need to fulfil certain characteristics: species need to be rainforest specialists, they should have limited dispersal abilities, and they need to have existed prior to the climatic events of interest. For example, to examine the impact of Plio‐ and Pleistocene aridification events, lineages should date back at least 5.33 million years.

The African frog genus *Conraua* comprises four species in the Upper Guinean forests of West Africa (*Conraua kamancamarai*, *Conraua alleni*, *Conraua sagyimase* and *Conraua derooi*). Their distribution range is patchy, allopatric and of varying size, but together they cover a large portion of the Upper Guinean biodiversity hotspot (Blackburn et al. 2020; Channing and Rödel 2019; Figure 1a). It encompasses areas of both highly complex and less heterogeneous topography, including montane and lowland rainforests (Figure 1a,b). *Conraua* are aquatic frogs living in permanent and fast‐flowing river streams. Tadpoles are usually found in the slow‐flowing or nearly stagnant sections of respective streams (Channing et al. 2012). West African *Conraua* species present limited terrestrial dispersal and are highly dependent on rainforests (Neira‐Salamea et al. 2022, 2021; Channing and Rödel 2019). Due to their close association with forested streams and their widespread occurrence in Upper Guinea, *Conraua* species are ideal candidates for studying the effect of past climatic fluctuations on rainforest‐adapted species. Based on the age of inter‐ and intraspecific splits (2–8 Myr) and the distribution patterns of these species, previous studies have speculated that climate‐driven rainforest fragmentation played an important role in shaping their diversification (Blackburn et al. 2020). The ecological similarities among the four species suggest that differences in their sensitivity or adaptability to changes in the environment are of no or only little importance. In particular, all species share a similar behaviour and dispersal abilities and are equally dependent on rainforests. Therefore, any variation in their responses is more likely due to environmental factors rather than differences in their ecological or evolutionary potential.

To better understand how past climatic change and landscape have shaped the diversity and distribution of rainforest taxa, we investigated phylogeographic patterns and quantified population dynamics within Upper Guinean *Conraua* species. Specifically, we predict that populations in heterogeneous landscapes (i.e., Fouta Djallon) present a deep phylogeographic structure, implying long‐term isolation and persistence. Populations should have smaller distribution ranges and fluctuate less over time in terms of population size. In contrast, rainforest cover likely fluctuated more extensively in homogeneous landscapes (i.e., western and eastern Upper Guinea; Figure 1a,b). We therefore predict less phylogeographic structure, visible at a larger spatial scale. Moreover, the dynamic history of these regions should be reflected in the demographic history of populations with stronger signatures of population expansion and contraction. To conduct phylogeographic and demographic analyses, we used double‐digest restriction site‐associated sequencing (ddRADseq). We generated a population‐level dataset for all West African *Conraua* species covering the majority of their distributional range. We investigate the spatial and demographic patterns in different regions in relation to the surrounding landscape characteristics. Finally, we discuss the conservation implications of our findings, which could significantly enhance the effectiveness of regional conservation strategies in the face of anthropogenic climate change.

## 2 Materials and Methods

### 2.1 Sampling and Sequencing

We used tissue samples stored in the collection of the MfN, Germany. We extracted DNA from 221 samples collected across the entire range of West African *Conraua* species (*C. kamancamarai*: 44 samples; *C. alleni* : 124; *C. sagyimase*: 12; *C. derooi* : 41). DNA was extracted from liver, muscle or toe clips using the QIAGEN DNeasy Blood and Tissue Kits. Samples were collected from 53 sites, and the average number of samples per site was eight. Species identification and metadata for the samples are summarised in Tables S2–S8.

To generate genomic data, we applied the ddRADseq protocol described by Peterson et al. (2012). Our library preparation procedure closely resembles those implemented in previous African frog phylogeography studies (Charles et al. 2018; Portik et al. 2017). Briefly, the process involved: (i) digesting 500 ng DNA with restriction enzymes SbfI and MspI, (ii) purifying the digested fragments with magnetic beads and ligating barcoded Illumina adapters, (iii) selecting fragments between 415 and 515 bp after accounting for adapter length, and (iv) quantifying the prepared libraries. The sequencing was conducted on a single Illumina HiSeq4000 lane (50 bp single‐end read sequencing with a final read length of 39 bp after trimming 11 bp restriction enzyme overhang and barcode).

After sequencing, we processed the raw Illumina reads using STACKS (V2.55; Catchen et al. 2011). First, we used USTACKS to generate stacks of loci for each individual based on the trimmed reads (39 bp). We built stacks if at least five reads (5× coverage) had a maximum of 2 bp mismatches (5% divergence). Next, we used CSTACKS to build a catalogue of loci and then we employed SSTACKS to assign loci to each sample. Subsequently, we used POPULATIONS to call single nucleotide polymorphisms (SNPs) that are present in at least 70% of the individuals using the ‐r flag. We removed any individuals with missing data for more than 50% of loci. One challenge with RADseq data is allele dropout, which can result from mutations in restriction sites or degraded DNA (Graham et al. 2015; Gautier et al. 2013). To evaluate and mitigate these effects, we ran the STACKS pipeline once for the entire dataset, once for each species, and once for the following two sister species comparisons: (i) *C. kamancamarai* and *C. alleni* and (ii) *C. derooi* and *C. sagyimase*. We performed population genetic analyses on the species‐specific datasets to increase the number of informative sites. However, in order to infer the species tree, to investigate interspecific gene flow and to estimate pairwise *F*_ST_ values, we employed the combined or the full datasets. Detailed statistics on loci recovery and coverage across datasets are shown in Tables S1–S8.

### 2.2 Nuclear Phylogeny

To explore the evolutionary relationships both within and between the study species, we used IQ‐TREE2 (Minh et al. 2020). The input FASTA files were generated by extracting sequences from the STACKS output and subsetting them into locus‐specific multi‐sequence alignments using a custom Python script. Each locus contained two haplotype sequences (h0 and h1), and we selected h0 for tree inference. The trees were built using the GTR + I + G model of substitution for 1000 bootstrap replicates. These analyses were carried out on the full dataset (Table S2), as well as on subsets including combinations of *C. kamancamarai* and *C. alleni* (Table S4), *C. derooi* and *C. sagyimase* (Table S3) and individual species datasets (Tables S5–S8). This was done to investigate evolutionary relationships at different taxonomic levels and to evaluate the impact of allele dropout caused by restriction site mutations.

### 2.3 Population Structure

**FIGURE 2:**
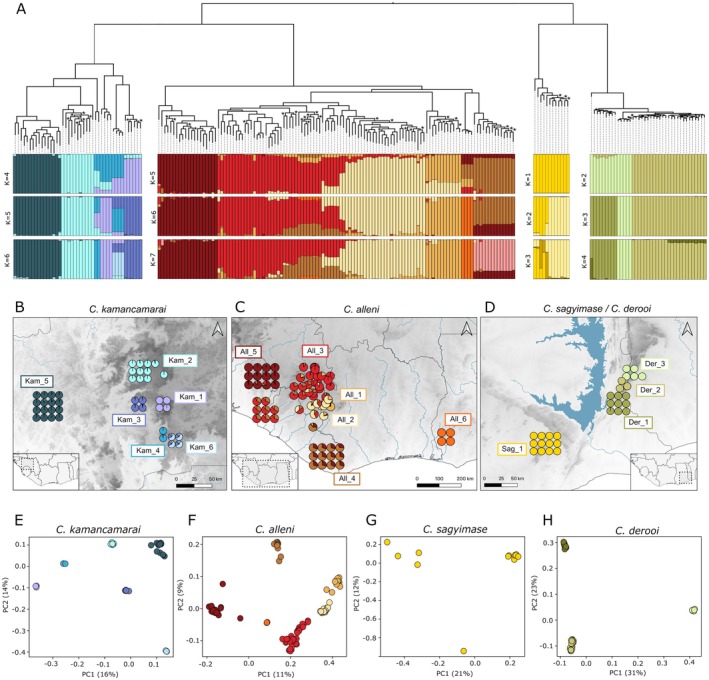
(A) Nuclear concatenated tree generated using IQ‐TREE2, including all four West African *Conraua* species (215 samples). Nodes with less than 95% bootstrap support are labelled with an asterisk. This tree is shown in combination with structure plots (tree tips are aligned with structure bars) under the 3 best *K* values of each species. (B–D) Admixture proportions from the structure plots shown as pie charts on the map (B: *Conraua kamancamarai*, C: *Conraua alleni*, D: *Conraua sagyimase*, and *Conraua derooi*). Labels indicate the IDs that were provided to each of the identified populations used in subsequent analyses. A zoomed‐in view of the Mount Nimba region is provided in Figure S17. (E–H) PCA plots for each species; populations were coloured following the same colour coding as in (A)–(D).

We employed two complementary approaches to characterise spatial population structure and investigate admixture levels. First, we conducted a principal component analysis (PCA) using PCAngsd (V0.98; Meisner and Albrechtsen 2018). The results were visualised using a customised Python script. Secondly, we employed STRUCTURE (Pritchard et al. 2000) to run a Bayesian model‐based clustering approach. To reduce linkage bias, we randomly selected a single SNP per RAD locus using VCFtools (V0.1.16; Danecek et al. 2011) for both analyses. We performed 10 independent replicates for each *K*‐value (ranging from 1 to 10) but increased the number of replicates to 20 if log‐likelihoods displayed substantial variance across runs. We ran STRUCTURE for 200,000 Markov chain Monte Carlo (MCMC) iterations and used a 10,000 burn‐in period. After the replicates were finished, we aligned them using CLUMPP (Jakobsson and Rosenberg 2007) and used the R package conStruct (Bradburd 2024) to plot them. Subsequently, we uploaded the STRUCTURE output to STRUCTURE HARVESTER (Earl and von Holdt 2012) to visualise the likelihood distribution under each *K*. We selected optimal *K* values based on multiple criteria: PCA results, STRUCTURE plots and their likelihood values across runs, as well as phylogenetic support (Figures S15 and S16). We refer to each cluster at the optimal *K* as a population, labelled with a species abbreviation and a numerical identifier (e.g., *C. alleni* populations are labelled All_1, All_2, etc.; *C. kamancamarai* as Kam_1, Kam_2, etc.; *C. derooi* as Der_1, Der_2, etc.; and *C. sagyimase* as Sag_1; Figure 2b–d; Table S9). We ran both population structure analyses on all dataset levels, from the full dataset comprising all West African *Conraua* species down to the species level, to ensure that any population substructure was detected (Figures S8–S14).

### 2.4 Demographic History

With population structure delineated for each species, we calculated summary statistics to assess patterns of genetic diversity, differentiation and potential deviations from neutrality. We used VCFtools (V0.1.16; Danecek et al. 2011) to compute pairwise *F*_ST_ (Weir and Cockerham 1984) and nucleotide diversity (π; Nei and Li 1979). To avoid underestimating genome‐wide diversity, we retained all SNPs per RAD locus. For estimating pairwise *F*_ST_ across all populations (inter‐ and intraspecific levels), we used the full dataset containing all species. For π, we subsetted the species‐specific VCF files to retain only individuals from the focal population (see Table S9 for population assignments). We calculated both statistics at the locus level and averaged them to obtain genome‐wide estimates. In addition, we also estimated neutrality statistics using the PopGenome R package (v2.7.5; Pfeifer et al. 2014). For this purpose, we split each species‐specific VCF file by scaffolds, concatenated the regions and defined populations using functions defined within this package. We then used the neutrality.stats() function to calculate Tajima's *D* (Tajima 1989), Fu and Li's F (Fu and Li 1993) and Fu and Li's D (Fu and Li 1993). To assess the significance of the observed neutrality statistics, we ran 1000 coalescent simulations under a neutral scenario using the Ms program (Hudson 2002). We considered that the deviation from neutrality was significant if the observed statistics fell within the 50 or 100 highest or lowest estimates out of the 1000 generated values. This would indicate a *p*‐value below 0.05 or 0.1, respectively.

For the demographic modelling, we tested alternative demographic histories using the diffusion approximation method of ∂a∂i (Gutenkunst et al. 2009) based on the folded site frequency spectrum (SFS). To account for linkage between sites and directly interpret likelihood and AIC values, we retained only a single randomly selected SNP per RAD locus (Coffman et al. 2016). In order to account for missing data and to maximise the number of segregating sites used in the analyses, we projected down the size of the populations. We did so by calculating the number of segregating sites in the SFS at each possible down projection and then selecting the number of alleles per population that maximises the size of the SFS. Based on the results from trial runs, we selected 10 as the minimum number of alleles that we could project down to. This means that in order to be run, each population had to retain at least 10 alleles after selecting the ideal down projection. We analysed both the one‐dimensional (1D; single population) and the two‐dimensional (2D; pairs of populations within species) SFS. In the 1D‐SFS models, we focused on each population separately and modelled neutral as well as 2‐ and 3‐epoch scenarios with changes in population size. In the 2D‐SFS models, we explored how population pairs diverged in terms of changes in population size and gene flow and modelled 1‐ and 2‐epoch models. Substructure within populations can influence demographic modelling in unpredictable ways, so we defined populations according to the clusters identified in the population structure analyses. Furthermore, we removed individuals showing recent signs of admixture (> 25% admixture proportion).

The optimisation process, based on Portik et al. (2017), consisted of three consecutive rounds. During the first round, we used threefold‐perturbed random starting parameters and carried out 50 independent optimisation replicates using the Nelder–Mead method. Each parameter optimisation step ran for a maximum of 20 iterations. During the second round, we repeated the same procedure but used the best‐scoring replicates from the previous run and a two‐fold random parameter perturbation. Finally, the last round of optimisation consisted of 100 independent optimisation replicates. As starting parameters, we selected the best‐scoring replicates from the second round after carrying out a one‐fold parameter perturbation. This optimisation procedure was the same for all 1D and 2D models, except for the standard 1D neutral model, which does not contain any parameters to be optimised. In this case, after extrapolating and calling the standard neutral model function, we calculated theta and log‐likelihood of the data given the model using the dadi.inference() module. Then, we calculated the AIC by multiplying the log‐likelihood by ‐2 (Akaike 1974). After the optimisation procedure was finalised, we summarised the statistics of each run by executing the summary and plotting functions available at github.com/dportik/dadi_pipeline. For the standard neutral model, we instead used the dadi.Plotting() module for visualisation.

## 3 Results

### 3.1 Sequencing Data

Using a ddRADseq approach, we generated a comprehensive population‐level genomic dataset covering most of Upper Guinea, from the Togo Hills in the East to the Fouta Djallon in the West (Figure 1a). We obtained approximately 703.26 million raw reads in total, with an average of 3.18 million reads per sample and a standard deviation of 1.37 million reads. After processing the raw data using the STACKS pipeline (including filtering for quality, intact restriction sites and sample barcodes), we produced seven distinct datasets: all West African *Conraua* species (215 samples; 32,010 SNPs; 6334 loci), *C. derooi* (40 samples; 7664 SNPs; 5364 loci), *C. sagyimase* (12 samples; 5375 SNPs; 4095 loci), *C. alleni* (120 samples; 42,827 SNPs; 16,578 loci), *C. kamancamarai* (43 samples; 27,946 SNPs; 12,781 loci), *C. derooi* –*C. sagyimase* (51 samples; 15,763 SNPs; 10,047 loci), and *C. alleni* –*C. kamancamarai* (154 samples; 42,382 SNPs; 9854 loci) (Table S1). These datasets allowed us to examine phylogeographic patterns across Upper Guinea, including regions previously unexplored at a genomic scale, such as the Fouta Djallon.

### 3.2 Population Structure and Phylogeographic Patterns

Our phylogenetic results align closely with Blackburn et al. (2020), confirming two sister lineages in Fouta Djallon and western Upper Guinea (*C. kamancamarai* and *C. alleni* , respectively) and two sister lineages in eastern Upper Guinea ( *C. derooi* and *C. sagyimase*, respectively) (Figure 2a). However, by sampling more localities, we were able to map the distribution of intraspecific populations with greater precision. Phylogeny, PCA and STRUCTURE analyses all revealed consistent intraspecific patterns (Figure 2, Figure S17). For *C. kamancamarai*, we identified five to six allopatric lineages situated in deep valleys with rainforest remnants. These lineages show high levels of intraspecific differentiation, evident from deep branches in the phylogenetic tree (Figure 2a) and elevated pairwise *F*_ST_ values (Table S12). Further east, in western Upper Guinea, we detected five distinct clusters of *C. alleni* . These include allopatric clusters (All_4, All_5), parapatric clusters (All_3 partially overlaps with All_1) and sympatric clusters (All_1 extensively overlaps with All_2) (Figure 2c). Interestingly, we also identified a *C. alleni* population in eastern Upper Guinea (All_6; Figure 2c). This population is separated from other *C. alleni* populations by approximately 500 km. The distribution gap spans nearly all of Côte d'Ivoire, and *Conraua* appears to be absent in this region. While the *C. alleni* clusters show notable differentiation and have a much broader range, they are less genetically distinct than the *C. kamancamarai* lineages (Figure 2a; Table S12). In eastern Upper Guinea, *C. derooi* and *C. sagyimase* exhibited limited distributions, confined to elevated areas (Figure 2d). We identified distinct clusters within *C. derooi* , whereas *C. sagyimase* formed a single cluster (Figure S15). Remarkably, *C. derooi* has much shorter branch lengths, indicating lower levels of intraspecific divergence (Figure 2a).

### 3.3 Demographic Dynamics

**FIGURE 3:**
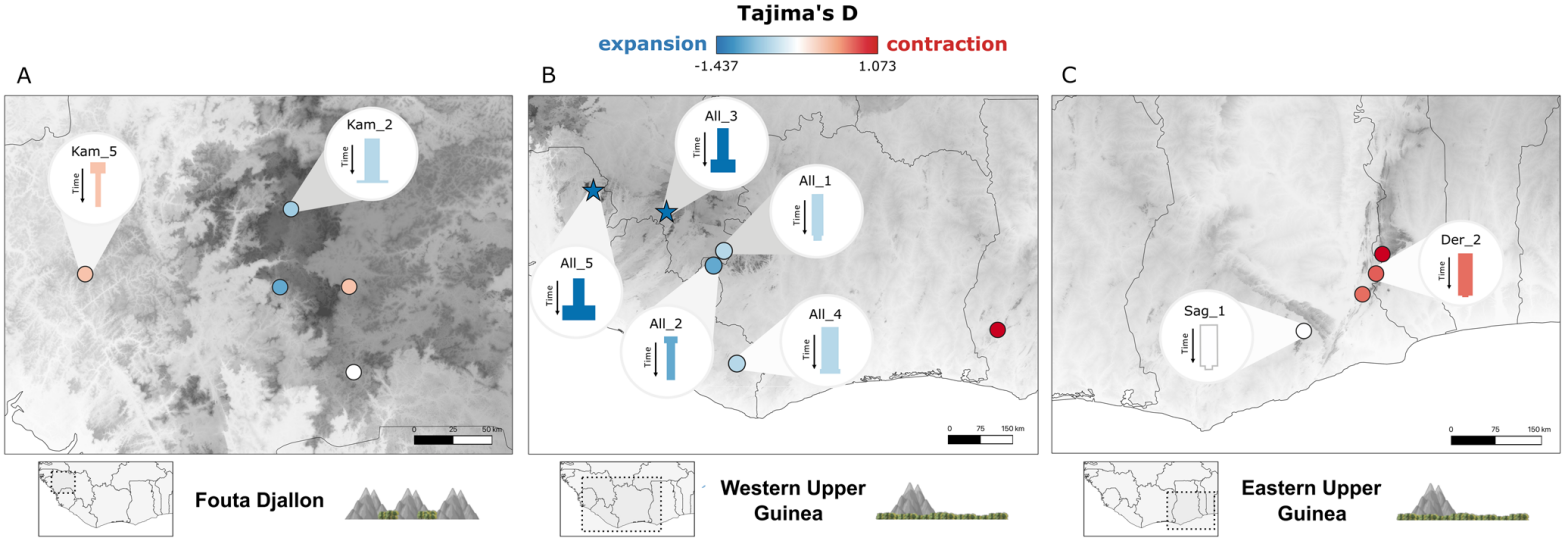
Demographic signatures of population size changes across Upper Guinea. This figure presents signatures of population size change across three regions: (A) Fouta Djallon, which is topographically complex across a broader region; (B) western Upper Guinea, where *Conraua alleni* inhabits both topographically complex areas and lowlands; (C) eastern Upper Guinea, with predominantly lowlands, but *Conraua* populations mainly constrained to topographically complex patches. Each population is represented by a dot on the map, coloured according to its Tajima's *D* value. Red indicates positive values (population contraction), and blue indicates negative values (population expansion). Significant Tajima's *D* values are symbolised with a star. For populations with sufficient data (> 10 alleles), additional plots show the inferred population size changes over time using 1D ∂a∂i models. Elevation is shown in greyscale: light areas indicate low altitude, dark areas indicate high altitude.

To assess the demographic processes underlying regional differences in Upper Guinea, we analysed population size dynamics and connectivity across species and regions using Tajima's *D* (Tajima 1989) and demographic modelling with ∂a∂i (Gutenkunst et al. 2009). Our primary question is whether there are shared demographic responses across populations, and if so, we aimed to identify the predominant demographic signatures in each region/species. *C. kamancamarai* inhabits the Fouta Djallon region, which is characterised by topographically complex terrain. In this region, *Conraua* are found in streams within deep valleys with rainforests. Demographic analyses revealed mixed signals among populations. For example, Kam_2 showed evidence of population expansion, while Kam_5 indicated signs of contraction (Figure 3a). These findings were supported by both Tajima's *D* values and demographic models, although the Tajima's *D* estimates were moderate and not statistically significant (Table S10; Figure 3a, Figures S23 and S24). Moving further east, *C. alleni* spans both lowland and montane areas with more topographically complex terrain, and we detected several populations with distribution ranges of varying sizes (Figure 2c). Populations with broad distributions (All_3, All_4 and All_5) consistently showed overall trends of population expansion (Figure 3b). All_3 and All_5 had significantly negative Tajima's *D* values (All_3: −1.43, *p* < 0.05; All_5: −1.12, *p* < 0.1; Table S10) and signs of secondary contact (Figures S28, S30 and S31). Furthermore, for all three populations, the best‐fitting 1D and several 2D ∂a∂i models present expansion events (Figures S20–S22, S29 and S31). Populations with narrower distributions (All_1 and All_2) presented less consistent results. All_1 and All_2 had a negative Tajima's *D*, suggesting expansion (Table S10) and several 2D models also indicated expansion (Figures S27 and S29) but the best‐fitting 1D models pointed to contraction (Figures S18, S19). In contrast to the overall trends of expansion observed in western Upper Guinea, the *C. alleni* population in eastern Upper Guinea (All_6) displayed strongly positive Tajima's *D* values, indicating contraction (Figure 3b; Table S10). This pattern aligns with trends in other species in the region, such as *C. sagyimase* and *C. derooi* , where demographic models and/or Tajima's *D* also indicated signs of population contraction (Figure 3c, Figure S26; Table S10). Overall, these results show distinct regional demographic patterns: mixed and moderate population size changes in the Fouta Djallon, predominant signs of expansion in western Upper Guinea and predominant signs of contraction in eastern Upper Guinea.

## 4 Discussion

Upper Guinean rainforests experienced cyclical climate‐driven changes in rainforest cover throughout the Miocene‐Pleistocene (23.03 Mya–10 Kya; Hardy et al. 2013). Previous studies relying on a few mitochondrial loci have provided indications of where refugia may be located across Upper Guinea (Hillers 2008), but the population dynamics underlying persistence in these refugia remain largely unexplored. For instance, it is unknown whether populations experienced similar changes in size across space and time. This study is the first to leverage genomic data to explore the phylogeographic and demographic history of any species across the entire Upper Guinean rainforest hotspot, including the Fouta Djallon. Our results corroborate multiple refugia and reveal regional differences in population dynamics. Broadly speaking, we found smaller changes in population size in populations inhabiting topographically complex regions, such as the Fouta Djallon. In contrast, we predominantly found signs of population expansion in western Upper Guinea and population decline in eastern Upper Guinea. Our results offer valuable insights into the interaction between climate and landscape in shaping evolutionary processes. Such information can aid in identifying key biodiversity areas and corridors and provide a foundation for conservation strategies that anticipate population responses to future climate changes.

### 4.1 Refugia Location

Climate‐driven rainforest fragmentation and persistence in refugia have left lasting imprints on contemporary phylogeographic patterns of Upper Guinean species (Hillers 2008; Nicolas et al. 2008). Persistence in separate refugia typically leads to divergence and to the formation of allopatric or parapatric phylogeographic lineages (Avise 2000; Hewitt 1996). We therefore examined the distribution of distinct phylogeographic lineages and interpreted them as proxies for possible refugia. We find distinct phylogeographic lineages, probably resulting from isolation in distinct refugia in: (a) Fouta Djallon (northern Guinea); (b) the Loma Mountains, Nimini Hills and Tingi Hills (Sierra Leone); (c) southeastern Sierra Leone to northwestern Liberia; (d) Mount Nimba (border region of Côte d'Ivoire, Guinea and Liberia); (e) eastern Liberia; (f) Ghana; and (g) the Ghana‐Togo border (Figure 2b–d). These phylogeographic results largely align with previous studies on the location of macro‐ and microrefugia (Figures 1b and 2b–d; Hillers 2008). However, we also found some unexpected phylogeographic patterns that warrant discussion. Mount Nimba has been postulated as an important refugium (Maley 1996; Sosef 1994), and Hillers (2008) suggested that two microrefugia in close proximity exist in this region. We identified two overlapping populations at Mount Nimba with signs of recent gene flow (Figure 2c, Figure S17). A similar distributional pattern has also been observed in sabre‐toothed frogs (genus *Odontobatrachus*) (Barej et al. 2015). Possibly, Mount Nimba is not only a region where populations have persisted but also a contact zone where populations expanding out of adjacent refugia meet. Another interesting finding is the disjunct distribution of *C. alleni* , which mirrors patterns seen in giant squeaker frogs ( *Arthroleptis krokosua* ; Sandberger‐Loua et al. 2018; Adum et al. 2011; Ernst et al. 2008). In our phylogenetic tree (Figure 2a), the *C. alleni* population in Ghana (All_6) appears as a sister lineage to All_4, but interestingly, pairwise *F*_ST_ values are lowest when comparing All_6 to All_3 and All_5 (Table S12). Additionally, the STRUCTURE plots (Figure 2a) show admixture between All_4, All_5 and All_6. This suggests recent or ongoing contact between eastern and western *C. alleni* populations, but we interpret these signals with caution. Despite targeted sampling efforts, *C. alleni* has not been detected in the intervening region. Furthermore, in our dataset All_6 is underrepresented and populations with smaller sample sizes tend to exhibit higher *F*_ST_ values (Table S12). Both the inflated *F*_ST_ values as well as admixture of All_6 at K=5 (Figure 2a) could be the result of uneven sampling (Lawson et al. 2018). Regardless of the contemporary connectivity among populations, the presence of deep lineages in western Ghana points to an unrecognised microrefugium. Furthermore, our results also identify an additional refugium in the Togo Hills, an often undervalued rainforest fragment (Segniagbeto et al. 2024; Hillers et al. 2009). To gain a comprehensive understanding of refugia dynamics and how populations responded to climatic changes, we further explored the demographic processes that have shaped these phylogeographic patterns.

### 4.2 The Role of Landscape in Modulating Population Response

By leveraging our dense sampling and genomic dataset, we were able to compare phylogeographic patterns and demographic trajectories across Upper Guinea, revealing a heterogeneous population response to climate fluctuations. Populations in the Fouta Djallon present narrow distributions, are highly divergent (Figure 2b) and show more variable demographic histories with less intense changes in population size compared to other regions (Figure 3a; Table S10). Populations in western Upper Guinea occupy larger areas and present signs of recent admixture and secondary contact, as well as moderate to strong signs of population expansion. Populations in eastern Upper Guinea have restricted distributions, exhibit relatively low genetic diversity and predominantly show signatures of declining population size (Figures 2a and 3c, Figures S25 and S26; Table S10). Given that Upper Guinean *Conraua* species have similar ecological properties, these regional differences are likely explained by factors that go beyond species ecology. Refugia dynamics can differ based on the landscape's complexity (Byrne et al. 2022; Byrne 2008). The Fouta Djallon is characterised by deep valleys with rainforests and high plateaus with savannas. This complex topography has climate‐buffering properties and provides a range of microenvironments where populations can persist (Trew and Maclean 2021; Byrne 2008). However, the same topography that fosters stability may also limit dispersal. As such, our results show much higher phylogeographic structure in a relatively small area in this region (Figure 2a,b). Several *C. kamancamarai* populations are only found in specific locations, and they are highly differentiated from each other, suggesting that they have persisted in these locations over relatively long time scales. This supports our hypothesis, showing that topographically complex regions are more likely to present signs of long‐term local persistence.

In contrast, western and eastern Upper Guinea contain a mix of mountains or hills interspersed with flatter areas, which could allow populations to track changes in the distribution of suitable habitat. This led us to hypothesise that populations in these regions would exhibit dynamic histories of expansion and contraction. In western Upper Guinea, we predominantly observed signals of population expansion, especially among populations distributed broadly in lowland rainforests. These populations likely had more restricted distributions in the past, potentially persisting in microrefugia, as suggested by Hillers (Hillers 2008; Figure 1b). While we also anticipated dynamic demographic histories in eastern Upper Guinea, our findings predominantly indicate population contraction, and populations show more restricted distributions. The observed signs of contraction imply that these populations are currently in a reduced state, suggesting they once occupied a wider range. The question arises as to why populations in western Upper Guinea expanded, while those in eastern Upper Guinea remain contracted. Western and eastern Upper Guinea differ substantially in contemporary rainfall levels (Figure 1c), which in turn shape the dominant forest types in each region (Poorter et al. 2004). The difference in demographic trends between western and eastern Upper Guinea may be linked to these contrasting overall moisture levels and habitat types. The higher rainfall in western Upper Guinea likely maintains suitable lowland habitat for *Conraua* today, whereas in eastern Upper Guinea, their distribution appears more restricted under the present environmental conditions. However, during wetter periods, such as humid phases in the Holocene, eastern Upper Guinean populations may have had broader ranges. This indicates that population response to climatic fluctuations may not only be shaped by landscape (i.e., heterogeneous vs. homogeneous) but also regional differences in climate (e.g., varying levels of overall rainfall).

### 4.3 Conservation Implications

**FIGURE 4:**
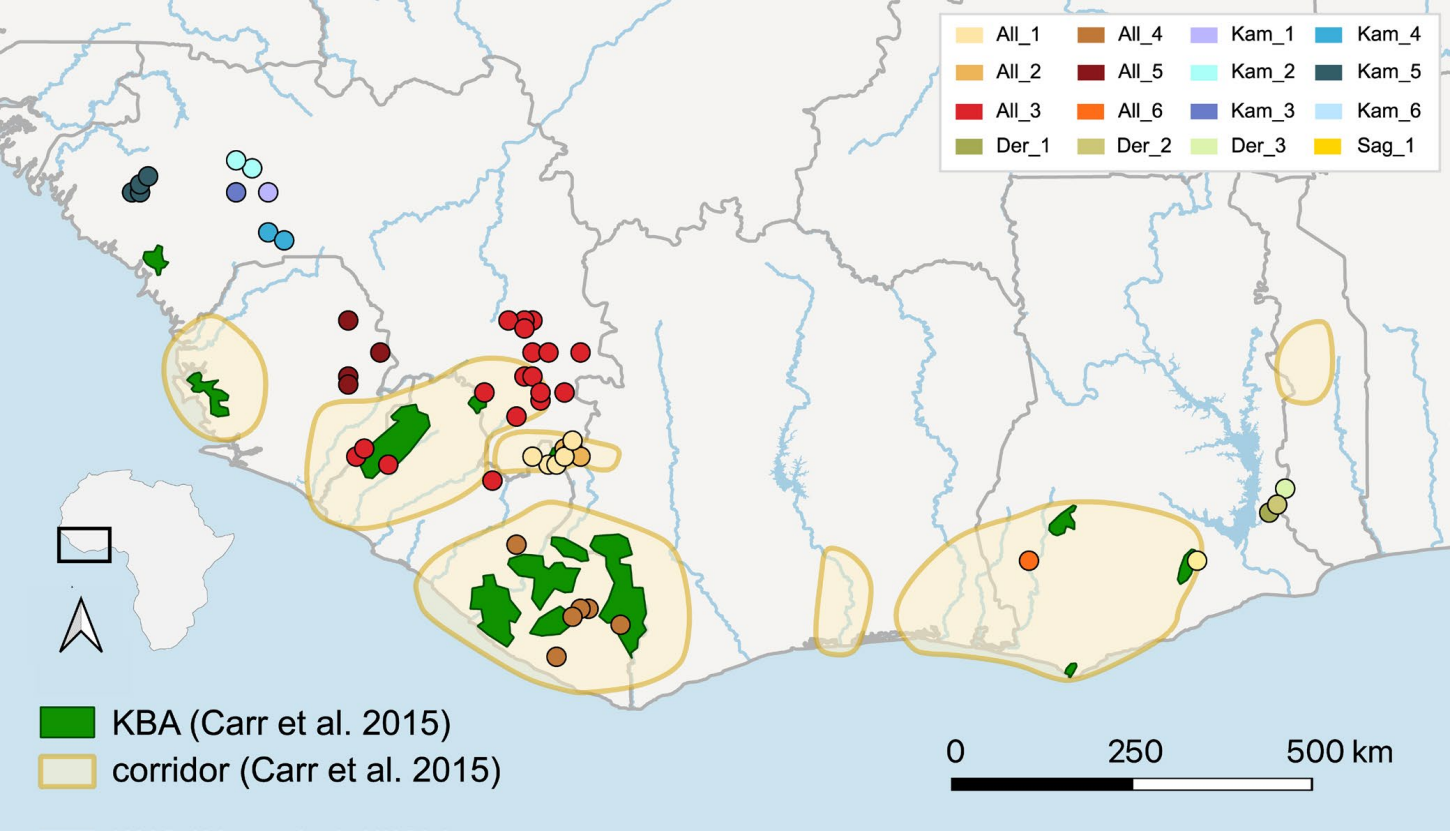
Map showing the distribution of the identified populations for each of our focal species. *Conraua kamancamarai* – five populations Kam_1—Kam_5, *Conraua alleni* – six populations All_1—All_6, *Conraua sagyimase* – one population Sag_1, *Conraua derooi* – three populations Der_1—Der_3. Sampling localities are coloured according to the colours assigned in Figure 2. The map also shows the distribution of key biodiversity areas (KBA's) and biodiversity corridors suggested by Carr et al. (2015). These are recommended priority areas, which does not mean that there are any effective conservation measures in place.

The growing threat of climate change is increasingly recognised as a critical factor affecting the health of many wild species (Wittig et al. 2007). Effective biodiversity conservation under future climate scenarios demands that we integrate an understanding of species responses to climate shifts directly into conservation strategies (Carr et al. 2014). The CEPF identifies and prioritises key biodiversity areas (KBAs) and biodiversity corridors, using species distribution data to assess where conservation needs are highest. KBAs represent sites that significantly contribute to the global persistence of biodiversity, while biodiversity corridors aim to maintain ecological and evolutionary processes across broader landscapes (Carr et al. 2015). In Upper Guinea, existing KBAs and corridors provide important conservation frameworks that generally align well with the results of our study (Figure 4). Most populations are captured partly by KBAs and most biodiversity corridors correspond with the layout of our populations. This suggests that these protected zones could indeed protect a large fraction of the genetic diversity within *Conraua* and maintain regional evolutionary processes. However, there are also populations that fall outside recommended KBAs and biodiversity corridors. This applies, for example, to populations in the Fouta Djallon (Figure 4). Populations in this area present deep, locally restricted lineages with evidence of long‐term persistence and stability through past climatic fluctuations. Any loss of rainforest habitat where these populations are found would lead to a significant loss of unique genetic diversity. We thus recommend that the localities should be considered for KBA status. In western Upper Guinea, KBAs and biodiversity corridors align well with our populations, but there is one exception. The *C. alleni* population in Sierra Leone (All_5) is currently not covered by any KBA nor biodiversity corridor despite harbouring unique genetic lineages. This montane region in Sierra Leone may therefore also warrant KBA designation and be a valuable additional biodiversity corridor. Lastly, preserving the last populations of unique genetic lineages in eastern Upper Guinea is critical. Some populations, like *C. sagyimase*, are found in KBAs but *C. derooi* and population All_6 show concerning population declines and may warrant KBA status as well. Overall, the current KBA and corridor model provide substantial support for *Conraua* conservation under climate change. However, we identify populations that fall outside these areas. Protecting them would prevent the loss of regionally unique biodiversity and essential evolutionary processes.

## 5 Conclusion

Upper Guinean rainforests have experienced turbulent climatic fluctuations that shaped the evolutionary trajectory of species. Understanding how rainforest‐dependent species responded to these changes is crucial, particularly as anthropogenic climate change intensifies. Here, we provide a detailed genomic and population‐level dataset on *Conraua*, a system well‐suited for examining the evolutionary effects of climate‐driven rainforest fragmentation (Blackburn et al. 2020). Our findings support the presence and relative age of previously postulated refugia (Hillers 2008) but reveal distinct demographic responses within them. In the Fouta Djallon, we see signs of stable, long‐term persistence, while populations in western and eastern Upper Guinea exhibit more dynamic demographic histories with evidence of contraction and expansion. These differences can be explained by differences in the regional landscape and suggest that topographic complexity is a key factor influencing the evolutionary dynamics within different refugia. Characterising refugia types and identifying the factors that underlie diverse population dynamics may enhance our ability to generalise findings and detect other refugia globally. This study exemplifies how genomics, dense sampling, and comparative phylogeographic approaches can offer insights into these dynamics and how this can contribute to conservation.

## Author Contributions

M.E., D.M.P., M.‐O.R., M.P.K.B., A.D.L. and M.K.F. conceptualised the study. A.D.L. conducted the lab work. D.M.P. carried out the primary data processing, and M.E. carried out the majority of the subsequent analyses. G.H.S., C.O.‐B., J.D., J.P., N.G.K. and M.‐O.R. provided tissue samples. M.E. wrote the manuscript and prepared the figures, with input from M.P.K.B. and M.‐O.R. All authors contributed to manuscript revision and approved the final version.

## Disclosure

Benefit‐sharing statement: The genetic resources examined in this study are legally accessioned in the herpetology collection of the MfN and were obtained during earlier projects under research, collection and export permits issued by the respective national authorities. Consequently, no additional permits were necessary for the present work. Research collaborations have been developed with scientists who collected the specimens or contributed data and expertise. All collaborators wishing to be involved are included as co‐authors. The new phylogeographic insights generated in this study will help guide regional conservation priorities and are being shared openly with the wider scientific community.

## Conflicts of Interest

The authors declare no conflicts of interest.

## Supporting information


Appendix S1

## Data Availability

Raw sequence reads and VCF files are deposited in Dryad (https://doi.org/10.5061/dryad.41ns1rnq). Sample metadata can be found in Tables S2–S8.
