# Supplementary material for: Comparative Phylogeography of West African Rainforest Frogs Reveals Regional Variation in Refugia Dynamics
Source: Mol Ecol. 2025 Jul 30;34(17):e70043. doi: 10.1111/mec.70043 (PMC12376955; doi:10.1111/mec.70043)
Supplement: Supplementary file 1 — Appendix S1 [file MEC-34-e70043-s001.pdf]

MOLECULAR ECOLOGY

Comparative phylogeography of West African rainforest frogs reveals regional variation in refugia dynamics

Mario Ernst, Daniel M. Portik, Gabriel H. Segniagbeto, Caleb Ofori-Boateng, Joseph Doumbia, Johannes Penner, N'Goran Paul Kouamé, Matthew K. Fujita, Adam D. Leaché, Mozes P.K. Blom & Mark-Oliver Rödel

Table of Contents:

**Table S1**	Page 2
**Table S2**	Pages 2-5
**Table S3**	Pages 6
**Table S4**	Pages 7-9
**Table S5**	Pages 10
**Table S6**	Pages 11-13
**Table S7**	Page 13
**Table S8**	Page 13-14
**Table S9**	Page 14
**Table S10**	Page 15
**Table S11**	Page 15
**Table S12**	Page 16
**Fig. S1**	Page 17
**Fig. S2**	Page 18
**Fig. S3**	Page 19
**Fig. S4**	Page 20
**Fig. S5**	Page 21
**Fig. S6**	Page 22
**Fig. S7**	Page 23
**Fig. S8**	Page 24
**Fig. S9**	Page 25
**Fig. S10**	Page 26
**Fig. S11**	Page 27
**Fig. S12**	Page 28
**Fig. S13**	Page 29
**Fig. S14**	Page 30
**Fig. S15**	Page 31
**Fig. S16**	Page 32
**Fig. S17**	Page 33
**Fig. S18**	Page 34
**Fig. S19**	Page 35
**Fig. S20**	Page 36
**Fig. S21**	Page 37
**Fig. S22**	Page 38
**Fig. S23**	Page 39
**Fig. S24**	Page 40
**Fig. S25**	Page 41
**Fig. S26**	Page 42
**Fig. S27**	Page 43
**Fig. S28**	Page 43
**Fig. S29**	Page 44
**Fig. S30**	Page 44
**Fig. S31**	Page 45
**Fig. S32**	Page 45

MOLECULAR ECOLOGY

Table S1: Summary of sample sizes, recovered loci and SNP counts in the final VCF's after applying missing data filters at the genotype and individual level. In each dataset, we retained only loci present in at least 70% of individuals and removed individuals with >50% missing data across sites.

Dataset	# samples	# loci	# SNPs
full dataset	215	6.334	32.010
C. sagyimase & *derooi*	51	10.047	15.763
C. alleni & *kamancamarai*	154	9.854	42.382
C. alleni	120	16.578	42.827
C. kamancamarai	43	12.781	27.946
C. derooi	40	5.364	7.664
C. sagyimase	12	4.095	5.375

Table S2: Sample information for the full dataset (*C. kamancamarai*, *C. alleni*, *C. sagyimase*, and *C. derooi*). Details include sample ID, sampling locality (latitude and longitude), species assignment, and sequencing statistics: frequency of missing data per sample in the final VCF's and mean coverage per site based on the BAM files.

Species	Sample ID	Latitude	Longitude	Freq. missing data (% loci)	Mean coverage
C. alleni	CB2010_180	8,351	-9,417	10,8	84,9
C. alleni	CB2010_181	8,351	-9,417	4,6	112,9
C. alleni	CB2010_182	8,351	-9,417	14,9	85,4
C. alleni	CB2010_183	8,351	-9,417	4,1	97,2
C. alleni	FO_5	8,531	-8,906	24,9	63,7
C. alleni	GN11_436	7,697	-8,396	4,5	117,6
C. alleni	GN11_439	7,712	-8,395	4,3	144,9
C. alleni	GN11_442	7,674	-8,375	6,7	104,8
C. alleni	GN11_443	7,646	-8,336	5,2	120,2
C. alleni	GN11_446	7,649	-8,424	5,1	92,9
C. alleni	GN11_447	7,649	-8,424	4,4	146,7
C. alleni	GN11_448	7,631	-8,414	4,7	135,9
C. alleni	GN11_453	7,631	-8,414	6,1	114,4
C. alleni	GN11_454	7,631	-8,414	14,6	76,7
C. alleni	GN11_457	7,633	-8,352	5,1	112,8
C. alleni	GN11_458	7,67	-8,354	5,1	136,1
C. alleni	GN11_459	7,633	-8,352	8,8	84,7
C. alleni	GN11_462	7,647	-8,368	6,4	101,8
C. alleni	GN11_463	7,647	-8,368	6,3	150,7
C. alleni	GN11_466	7,647	-8,368	5,6	163,3
C. alleni	GN11_469	7,615	-8,45	5,7	134,4
C. alleni	GN11_470	7,615	-8,45	7,5	115,4
C. alleni	GN11_473	7,624	-8,418	9,7	96,5
C. alleni	GN11_474	7,624	-8,418	6,7	124,7
C. alleni	GN11_478	7,624	-8,418	5,8	130,9
C. alleni	GN11_482	7,67	-8,374	4,3	148,8
C. alleni	GN11_483	7,677	-8,385	4,4	144,8
C. alleni	GN11_484	7,593	-8,468	3,8	151
C. alleni	GN11_485	7,593	-8,468	3,5	135,1
C. alleni	GN11_489	7,671	-8,395	4,2	113,3
C. alleni	GN11_490	7,671	-8,395	4	153
C. alleni	GN11_493	7,677	-8,385	5	115,1
C. alleni	GN11_494	7,677	-8,385	5,1	111,3
C. alleni	GO20	7,453	-10,692	37,3	45,9
C. alleni	GOL12	7,597	-11,023	47,8	22,8
C. alleni	GRE39	5,405	-7,73	10,8	71,2
C. alleni	GRE44	5,405	-7,73	42,6	58,3
C. alleni	GS096	7,658	-10,903	29,3	44,4
C. alleni	GS111	7,658	-10,905	4,1	111,6

MOLECULAR ECOLOGY

C. alleni	GS112	7,658	-10,905	3,8	131,5
C. alleni	GS113	7,658	-10,905	4,1	120,4
C. alleni	GS114	7,658	-10,905	3,7	111,3
C. alleni	GS90	7,658	-10,903	13,1	91,6
C. alleni	JP0142	8,501	-11,147	5,5	107,4
C. alleni	JP0143	8,501	-11,147	19,1	59,7
C. alleni	JP7_122	8,354	-9,416	5,8	127,1
C. alleni	JP7_123	8,354	-9,416	5,2	111,7
C. alleni	JP7_154	8,554	-8,888	5,4	107,7
C. alleni	JP7_191	8,365	-8,433	12,6	99
C. alleni	LI006	5,662	-8,161	8,4	115,5
C. alleni	LI007	5,662	-8,161	6,7	132,4
C. alleni	LI008	5,662	-8,161	14,7	77,6
C. alleni	LI009	5,662	-8,161	12,4	78,4
C. alleni	LI10_032	7,458	-8,666	4,1	125,6
C. alleni	LI10_034	7,445	-8,658	11	109,1
C. alleni	LI10_068	7,441	-8,593	5,1	119,7
C. alleni	LI10_117	7,484	-8,577	7,8	100,1
C. alleni	LI10_122	7,441	-8,593	7,5	108,5
C. alleni	LI10_138	7,483	-8,537	8,6	104,6
C. alleni	LI157	5,688	-8,215	7,4	157,7
C. alleni	LOM107	9,224	-11,159	41,7	42,9
C. alleni	LOM52	9,213	-11,144	20,9	96,9
C. alleni	LOM55	9,213	-11,144	7,4	101,7
C. alleni	LOM66	9,209	-11,173	7,6	98,4
C. alleni	LOM67	9,209	-11,173	11,2	99,3
C. alleni	LOM89	9,213	-11,144	9,4	89,3
C. alleni	LOM90	9,213	-11,144	10,6	109,4
C. alleni	MH0065	7,596	-8,263	10,9	75,3
C. alleni	MH0081	7,544	-8,838	6,2	79,6
C. alleni	MH0082	7,544	-8,838	3,9	134,7
C. alleni	MH0096	8,283	-8,738	5,2	120,1
C. alleni	MH0108	8,334	-8,709	8,4	122
C. alleni	MH0124	8,318	-8,713	6,5	144,9
C. alleni	MH0125	8,318	-8,713	8,1	128,4
C. alleni	MH0126	8,815	-8,862	9,1	117,2
C. alleni	MH0127	8,815	-8,862	18,5	96,5
C. alleni	MH0128	8,815	-8,862	5,6	101,7
C. alleni	MH0140	8,884	-8,294	7,6	121,5
C. alleni	MH0141	8,884	-8,294	4,9	128,5
C. alleni	MTN11	7,714	-8,362	48,7	60,9
C. alleni	MTN190	7,59	-8,452	3,9	135,2
C. alleni	MTN208	7,59	-8,452	4,1	122
C. alleni	MTN5	7,714	-8,362	42,8	75
C. alleni	NIM121	8,511	-11,102	28,1	60,5
C. alleni	NIM87	8,501	-11,147	6,5	116,8
C. alleni	NIM95	8,499	-11,151	6,7	112,9
C. alleni	NIM98	8,499	-11,151	10,5	81,5
C. alleni	P_LI_12_409	6,443	-9,056	8,7	111,9
C. alleni	P_LI_12_458	5,643	-8,208	8,3	117
C. alleni	P_LI_12_480	5,647	-8,207	9,8	85,8
C. alleni	PB_11_10	8,043	-9,076	4,5	109,5
C. alleni	PB_11_104	8,043	-9,076	4,8	107,3
C. alleni	PB_11_11	8,043	-9,076	7,8	76,5
C. alleni	PB_11_267	7,235	-9,31	5,8	107
C. alleni	PB_11_40	8,043	-9,076	7,5	113,5
C. alleni	PB_11_459	9,208	-8,926	7,1	95,8
C. alleni	PB_11_462	9,21	-8,927	8	77
C. alleni	PB_11_474	9,263	-8,931	7	101

MOLECULAR ECOLOGY

C. alleni	PB_11_584	9,252	-8,874	8,1	96,8
C. alleni	PB_11_9	8,043	-9,076	6,1	102,1
C. alleni	PB_11_924	9,262	-8,928	5,4	123,9
C. alleni	PG_L_13_066	5,074	-8,545	11,3	101,9
C. alleni	PG_L_13_067	5,077	-8,54	11	99,4
C. alleni	PG_L_13_174	5,082	-8,501	16,2	77,7
C. alleni	PLI_12_277	5,52	-8,345	9,2	106,6
C. alleni	SI21	9,28	-9,114	4,9	122,2
C. alleni	SI25	9,28	-9,114	9,9	93,1
C. alleni	SI41	9,161	-8,933	7,5	84,3
C. alleni	SI69	8,889	-8,616	4,9	59,9
C. alleni	SI78	8,881	-8,295	17,8	56,5
C. alleni	SSF_001	6,234	-2,69	44,5	59,6
C. alleni	SSF_002	6,236	-2,695	39,6	59,4
C. alleni	SSF_003	6,238	-2,696	41	61,2
C. alleni	SSF_005	6,236	-2,696	41,4	65,3
C. alleni	TI10	8,89	-10,79	12,4	60,5
C. alleni	TI11	8,89	-10,79	6,7	101,2
C. alleni	TI17	8,89	-10,79	6,9	92,4
C. alleni	TI62	8,89	-10,79	7,8	98,6
C. alleni	TI63	8,89	-10,79	11,1	75,1
C. alleni	TI66	8,865	-10,792	8,8	79,2
C. alleni	TI67	8,867	-10,792	9,4	93,2
C. derooi	AM01	6,85	0,446	19,1	112,6
C. derooi	AM03	6,85	0,446	23,8	102,8
C. derooi	AM06	6,85	0,446	20,9	87,3
C. derooi	AM07	6,85	0,446	25,7	57,2
C. derooi	AM08	6,85	0,446	19,8	96,4
C. derooi	AM09	6,85	0,446	24,6	75
C. derooi	AM12	6,85	0,446	20,5	101,7
C. derooi	AM18	6,85	0,446	21,3	84,8
C. derooi	AM55	6,845	0,438	23,2	91,4
C. derooi	Anedi_1	6,933	0,566	17,2	85,7
C. derooi	Anedi_2	6,933	0,566	17,5	78,1
C. derooi	Anedi_3	6,933	0,566	18	77,7
C. derooi	Anedi_4	6,933	0,566	19,2	69,5
C. derooi	Anedi_5	6,933	0,566	20,5	65,9
C. derooi	Douane_1	6,948	0,575	23,9	61
C. derooi	Douane_3	6,948	0,575	24,2	58
C. derooi	Douane_4	6,948	0,575	23	55,1
C. derooi	Douane_5	6,948	0,575	24,1	50,9
C. derooi	Kamalo_1	6,951	0,598	16,8	74,3
C. derooi	Kamalo_2	6,951	0,598	24,8	51,9
C. derooi	Kamalo_3	6,951	0,598	18,5	60,7
C. derooi	Kamalo_4	6,951	0,598	23,2	56,8
C. derooi	Kamalo_5	6,951	0,598	19,2	63
C. derooi	Kamalo_6	6,951	0,598	18	67,4
C. derooi	TOG76	6,952	0,565	18,3	87,6
C. derooi	TOG78	6,952	0,565	21,5	77,8
C. derooi	TOG79	6,952	0,565	20,8	74,6
C. derooi	TOG80	6,952	0,565	32	68,9
C. derooi	TOG81	6,952	0,565	33,9	68,1
C. derooi	TOG86	6,952	0,565	23,2	84,1
C. derooi	Yikpa_1	7,128	0,601	18	72,7
C. derooi	Yikpa_2	7,128	0,601	21	54,3
C. derooi	Yikpa_3	7,128	0,601	20,4	58
C. derooi	Yikpa_4	7,128	0,601	20,2	55,1
C. derooi	Yikpa_5	7,128	0,601	34,9	52,6
C. derooi	Zoto_1	6,943	0,575	24,8	51

C. derooi	Zoto_2	6,943	0,575	21,3	53,9
C. derooi	Zoto_3	6,943	0,575	22,5	57,4
C. derooi	Zoto_4	6,943	0,575	19,3	73,5
C. kamancamarai	CB2010_001	11,296	-12,507	18,4	75,9
C. kamancamarai	CB2010_002	11,296	-12,507	18,4	100,7
C. kamancamarai	CB2010_016	11,296	-12,513	21,9	111,1
C. kamancamarai	CB2010_017	11,296	-12,513	26,5	93,7
C. kamancamarai	CB2010_030	11,287	-12,504	18,5	108,5
C. kamancamarai	CB2010_044	11,192	-12,316	22,3	101,6
C. kamancamarai	CB2010_055	10,851	-12,521	29,1	92
C. kamancamarai	CB2010_056	10,851	-12,521	27,2	101
C. kamancamarai	CB2010_057	10,851	-12,521	22,2	91,9
C. kamancamarai	CB2010_059	10,851	-12,521	18,2	119,3
C. kamancamarai	CB2010_061	10,851	-12,521	22,7	90,2
C. kamancamarai	CB2010_082	10,84	-12,556	21,1	209,2
C. kamancamarai	CB2010_088	10,82	-12,192	22,8	98,6
C. kamancamarai	CB2010_089	10,82	-12,192	20,6	102,2
C. kamancamarai	CB2010_090	10,82	-12,192	18,8	115,5
C. kamancamarai	CB2010_091	10,82	-12,192	20,3	109,8
C. kamancamarai	FD27	11,295	-12,513	22,7	79,1
C. kamancamarai	FD4	11,295	-12,513	19,9	86,6
C. kamancamarai	FD44	11,295	-12,513	29,3	79,9
C. kamancamarai	FD45	11,295	-12,513	19,8	105,4
C. kamancamarai	FD48	11,295	-12,513	16,7	77,2
C. kamancamarai	GN11_130	10,341	-12,171	27,3	157
C. kamancamarai	GN11_140	10,341	-12,171	21,5	101,7
C. kamancamarai	GN11_176	10,297	-11,942	24,6	93,1
C. kamancamarai	GN11_179	10,297	-11,942	22,5	125,6
C. kamancamarai	GN11_184	10,297	-11,942	22,3	164
C. kamancamarai	GN11_188	10,297	-11,942	22,3	190,7
C. kamancamarai	KD061	10,844	-13,809	17,3	94,8
C. kamancamarai	KD062	10,844	-13,809	20,3	87,7
C. kamancamarai	KD063	10,844	-13,809	16,5	76,8
C. kamancamarai	KD069	10,835	-13,742	21,1	84,7
C. kamancamarai	KD070	10,835	-13,742	16,4	71,9
C. kamancamarai	KD071	10,855	-13,742	17,1	100,4
C. kamancamarai	KD076	10,865	-13,741	15,7	128,6
C. kamancamarai	KD077	10,865	-13,741	17,8	164
C. kamancamarai	KD078	10,865	-13,741	15,2	153,3
C. kamancamarai	KD079	10,865	-13,741	15,7	143,6
C. kamancamarai	KD081	10,877	-13,72	19,4	103,3
C. kamancamarai	KD084	10,958	-13,711	19,1	103,9
C. kamancamarai	KD092	10,974	-13,708	18,1	179
C. kamancamarai	KD124	11,075	-13,671	16,7	57,6
C. kamancamarai	KD125	11,075	-13,671	16,1	114,1
C. kamancamarai	KD126	11,075	-13,671	18,2	112,4
C. sagyimase	155	6,262	-0,555	32,8	102,8
C. sagyimase	212	6,271	-0,565	36	75,2
C. sagyimase	213	6,271	-0,565	25,2	74,7
C. sagyimase	217	6,271	-0,565	29,5	67,9
C. sagyimase	218	6,271	-0,565	28,9	91,5
C. sagyimase	AT01	6,207	-0,577	29,2	83,8
C. sagyimase	AT02	6,207	-0,577	31,4	77,4
C. sagyimase	AT04	6,207	-0,577	33,2	78
C. sagyimase	AT09	6,207	-0,577	34,3	62,6
C. sagyimase	AT11	6,207	-0,577	32,9	68,1
C. sagyimase	AT13	6,207	-0,577	41,5	86,7
C. sagyimase	AT20	6,207	-0,577	29,9	79,2

MOLECULAR ECOLOGY

Table S3: Sample information for the dataset comprising samples of *C. sagyimase*, and *C. derooi*. Details include sample ID, sampling locality (latitude and longitude), species assignment, and sequencing statistics: frequency of missing data per sample in the final VCF's and mean coverage per site based on the BAM files.

Species	Sample ID	Latitude	Longitude	Freq. missing data (% loci)	Mean coverage
C. derooi	AM01	6,85	0,45	35,9	112
C. derooi	AM03	6,85	0,45	42,1	102,8
C. derooi	AM06	6,85	0,45	36,5	86,7
C. derooi	AM07	6,85	0,45	41,2	56,9
C. derooi	AM08	6,85	0,45	38,4	96,6
C. derooi	AM09	6,85	0,45	39	74,6
C. derooi	AM12	6,85	0,45	35,1	101,2
C. derooi	AM18	6,85	0,45	34,8	84,2
C. derooi	AM55	6,85	0,44	45,2	91
C. derooi	Anedi_1	6,93	0,57	31,9	85,2
C. derooi	Anedi_2	6,93	0,57	31,8	77,4
C. derooi	Anedi_3	6,93	0,57	32,1	77,2
C. derooi	Anedi_4	6,93	0,57	32,7	69
C. derooi	Anedi_5	6,93	0,57	33,3	65,6
C. derooi	Douane_1	6,95	0,58	35,2	60,7
C. derooi	Douane_3	6,95	0,58	35,8	57,6
C. derooi	Douane_4	6,95	0,58	34,8	54,7
C. derooi	Douane_5	6,95	0,58	35,8	50,6
C. derooi	Kamalo_1	6,95	0,6	31,7	73,8
C. derooi	Kamalo_2	6,95	0,6	36	51,5
C. derooi	Kamalo_3	6,95	0,6	32,7	60,4
C. derooi	Kamalo_4	6,95	0,6	35,1	56,3
C. derooi	Kamalo_5	6,95	0,6	32,7	62,7
C. derooi	Kamalo_6	6,95	0,6	31,9	67,1
C. derooi	TOG76	6,95	0,56	42,5	87,3
C. derooi	TOG78	6,95	0,56	34	77,4
C. derooi	TOG79	6,95	0,56	33,6	74,1
C. derooi	TOG80	6,95	0,56	42,3	68,4
C. derooi	TOG81	6,95	0,56	43,5	67,8
C. derooi	TOG86	6,95	0,56	36,4	83,5
C. derooi	Yikpa_1	7,13	0,6	26,8	72,3
C. derooi	Yikpa_2	7,13	0,6	28,1	53,8
C. derooi	Yikpa_3	7,13	0,6	27,5	57,5
C. derooi	Yikpa_4	7,13	0,6	27,6	54,6
C. derooi	Yikpa_5	7,13	0,6	37,8	52,1
C. derooi	Zoto_1	6,94	0,57	36,1	50,4
C. derooi	Zoto_2	6,94	0,57	33,8	53,3
C. derooi	Zoto_3	6,94	0,57	34,9	56,8
C. derooi	Zoto_4	6,94	0,57	34	72,7
C. sagyimase	155	6,26	-0,56	39	104,4
C. sagyimase	212	6,27	-0,56	38,2	76,9
C. sagyimase	213	6,27	-0,56	33,3	76,3
C. sagyimase	217	6,27	-0,56	35,9	69,9
C. sagyimase	218	6,27	-0,56	33,7	93,8
C. sagyimase	AT01	6,21	-0,58	32,6	86,2
C. sagyimase	AT02	6,21	-0,58	33,4	79,2
C. sagyimase	AT04	6,21	-0,58	34,2	80,5
C. sagyimase	AT09	6,21	-0,58	36,1	64,5
C. sagyimase	AT11	6,21	-0,58	34,1	70,2
C. sagyimase	AT13	6,21	-0,58	42,5	89
C. sagyimase	AT20	6,21	-0,58	32,5	81,4

MOLECULAR ECOLOGY

Table S4: Sample information for the dataset comprising samples of *C. kamancamarai*, and *C. alleni*. Details include sample ID, sampling locality (latitude and longitude), species assignment, and sequencing statistics: frequency of missing data per sample in the final VCF's and mean coverage per site based on the BAM files.

Species	Sample ID	Latitude	Longitude	Freq. missing data (% loci)	Mean coverage
C. alleni	CB2010_180	8,351	-9,417	13	85,4
C. alleni	CB2010_181	8,351	-9,417	5,6	113,8
C. alleni	CB2010_182	8,351	-9,417	18,6	86,1
C. alleni	CB2010_183	8,351	-9,417	4,7	98
C. alleni	FO_5	8,531	-8,906	28,8	64,1
C. alleni	GN11_436	7,697	-8,396	4,7	118,2
C. alleni	GN11_439	7,712	-8,395	4,4	145,4
C. alleni	GN11_442	7,674	-8,375	8,7	105,7
C. alleni	GN11_443	7,646	-8,336	6,1	121,8
C. alleni	GN11_446	7,649	-8,424	6,1	93,9
C. alleni	GN11_447	7,649	-8,424	5	148
C. alleni	GN11_448	7,631	-8,414	5,3	137
C. alleni	GN11_453	7,631	-8,414	6,9	114,9
C. alleni	GN11_454	7,631	-8,414	17,7	77,2
C. alleni	GN11_457	7,633	-8,352	6,1	113,1
C. alleni	GN11_458	7,67	-8,354	6,3	137,4
C. alleni	GN11_459	7,633	-8,352	11,1	85,5
C. alleni	GN11_462	7,647	-8,368	7,3	102,2
C. alleni	GN11_463	7,647	-8,368	6,3	151,4
C. alleni	GN11_466	7,647	-8,368	6	164,8
C. alleni	GN11_469	7,615	-8,45	5,7	135,6
C. alleni	GN11_470	7,615	-8,45	8,1	116
C. alleni	GN11_473	7,624	-8,418	11,3	96,9
C. alleni	GN11_474	7,624	-8,418	7,3	125,7
C. alleni	GN11_478	7,624	-8,418	6	130,6
C. alleni	GN11_482	7,67	-8,374	4,4	149,7
C. alleni	GN11_483	7,677	-8,385	4,4	145,5
C. alleni	GN11_484	7,593	-8,468	3,6	152,1
C. alleni	GN11_485	7,593	-8,468	3,9	136,2
C. alleni	GN11_489	7,671	-8,395	4,5	114,1
C. alleni	GN11_490	7,671	-8,395	3,8	153,8
C. alleni	GN11_493	7,677	-8,385	5,4	115,7
C. alleni	GN11_494	7,677	-8,385	5,8	112,4
C. alleni	GRE39	5,405	-7,73	13,9	71,3
C. alleni	GS096	7,658	-10,903	34	44,8
C. alleni	GS111	7,658	-10,905	4,3	112
C. alleni	GS112	7,658	-10,905	4,4	131,9
C. alleni	GS113	7,658	-10,905	4,3	121,1
C. alleni	GS114	7,658	-10,905	4,2	112
C. alleni	GS90	7,658	-10,903	15,9	92
C. alleni	JP0142	8,501	-11,147	5,9	107,8
C. alleni	JP0143	8,501	-11,147	23,4	60
C. alleni	JP7_122	8,354	-9,416	7,1	128,3
C. alleni	JP7_123	8,354	-9,416	7	112
C. alleni	JP7_154	8,554	-8,888	7	107,9
C. alleni	JP7_191	8,365	-8,433	16,9	99,5
C. alleni	LI006	5,662	-8,161	10,1	116,2
C. alleni	LI007	5,662	-8,161	8,1	133
C. alleni	LI008	5,662	-8,161	18,3	77,7
C. alleni	LI009	5,662	-8,161	15,6	78,7
C. alleni	LI10_032	7,458	-8,666	4,6	126,6

C. alleni	LI10_034	7,445	-8,658	13,5	110,6
C. alleni	LI10_068	7,441	-8,593	6	120,8
C. alleni	LI10_117	7,484	-8,577	9,8	101
C. alleni	LI10_122	7,441	-8,593	9	109,5
C. alleni	LI10_138	7,483	-8,537	11,1	105,6
C. alleni	LI157	5,688	-8,215	8,8	158,2
C. alleni	LOM52	9,213	-11,144	24,8	97,4
C. alleni	LOM55	9,213	-11,144	7,8	101,5
C. alleni	LOM66	9,209	-11,173	8	98,5
C. alleni	LOM67	9,209	-11,173	12,6	99,6
C. alleni	LOM89	9,213	-11,144	11,2	89,4
C. alleni	LOM90	9,213	-11,144	12,5	110,4
C. alleni	MH0065	7,596	-8,263	12,2	75,4
C. alleni	MH0081	7,544	-8,838	7,2	80,3
C. alleni	MH0082	7,544	-8,838	4	135,9
C. alleni	MH0096	8,283	-8,738	6	121,1
C. alleni	MH0108	8,334	-8,709	10,2	122,7
C. alleni	MH0124	8,318	-8,713	8	145,5
C. alleni	MH0125	8,318	-8,713	9,8	129,7
C. alleni	MH0126	8,815	-8,862	11	118
C. alleni	MH0127	8,815	-8,862	21,4	96,5
C. alleni	MH0128	8,815	-8,862	6,9	102,1
C. alleni	MH0140	8,884	-8,294	9,6	121,9
C. alleni	MH0141	8,884	-8,294	5,8	129,4
C. alleni	MTN190	7,59	-8,452	4	136,7
C. alleni	MTN208	7,59	-8,452	4,2	122,7
C. alleni	NIM121	8,511	-11,102	31,9	60,7
C. alleni	NIM87	8,501	-11,147	6,9	116,9
C. alleni	NIM95	8,499	-11,151	8,6	113,2
C. alleni	NIM98	8,499	-11,151	12,5	81,8
C. alleni	P_LI_12_409	6,443	-9,056	9,6	112,7
C. alleni	P_LI_12_458	5,643	-8,208	9,6	117,9
C. alleni	P_LI_12_480	5,647	-8,207	12,3	86,4
C. alleni	PB_11_10	8,043	-9,076	5	110,2
C. alleni	PB_11_104	8,043	-9,076	5,1	108,1
C. alleni	PB_11_11	8,043	-9,076	9,3	77
C. alleni	PB_11_267	7,235	-9,31	7,1	107,7
C. alleni	PB_11_40	8,043	-9,076	9,8	114,4
C. alleni	PB_11_459	9,208	-8,926	7,8	96,4
C. alleni	PB_11_462	9,21	-8,927	9,7	77,9
C. alleni	PB_11_474	9,263	-8,931	8,8	101,3
C. alleni	PB_11_584	9,252	-8,874	9,2	97,6
C. alleni	PB_11_9	8,043	-9,076	7,5	103,3
C. alleni	PB_11_924	9,262	-8,928	6,2	125
C. alleni	PG_L_13_066	5,074	-8,545	13	102,3
C. alleni	PG_L_13_067	5,077	-8,54	12,6	100,5
C. alleni	PG_L_13_174	5,082	-8,501	19	78,5
C. alleni	PLI_12_277	5,52	-8,345	10,4	107,6
C. alleni	SI21	9,28	-9,114	5,6	122,9
C. alleni	SI25	9,28	-9,114	14,2	94
C. alleni	SI41	9,161	-8,933	9	84,6
C. alleni	SI69	8,889	-8,616	6	60,3
C. alleni	SI78	8,881	-8,295	21,5	57,1
C. alleni	TI10	8,89	-10,79	14,5	60,9
C. alleni	TI11	8,89	-10,79	7,6	101,5
C. alleni	TI17	8,89	-10,79	7,4	92,7
C. alleni	TI62	8,89	-10,79	8,7	98,8
C. alleni	TI63	8,89	-10,79	13,1	75,6
C. alleni	TI66	8,865	-10,792	11	79,7

C. alleni	TI67	8,867	-10,792	12	93,7
C. kamancamarai	CB2010_001	11,296	-12,507	25,1	76,2
C. kamancamarai	CB2010_002	11,296	-12,507	25	101
C. kamancamarai	CB2010_016	11,296	-12,513	30,1	112,1
C. kamancamarai	CB2010_017	11,296	-12,513	34,6	94,1
C. kamancamarai	CB2010_030	11,287	-12,504	25,1	108,8
C. kamancamarai	CB2010_044	11,192	-12,316	30,1	102
C. kamancamarai	CB2010_055	10,851	-12,521	37,5	91,7
C. kamancamarai	CB2010_056	10,851	-12,521	35,2	100,5
C. kamancamarai	CB2010_057	10,851	-12,521	28,3	92,2
C. kamancamarai	CB2010_059	10,851	-12,521	22,6	119,9
C. kamancamarai	CB2010_061	10,851	-12,521	29,4	90,4
C. kamancamarai	CB2010_082	10,84	-12,556	27,2	209,5
C. kamancamarai	CB2010_088	10,82	-12,192	29,9	98,5
C. kamancamarai	CB2010_089	10,82	-12,192	27,5	102,1
C. kamancamarai	CB2010_090	10,82	-12,192	25,6	115,7
C. kamancamarai	CB2010_091	10,82	-12,192	26,8	109,7
C. kamancamarai	FD27	11,295	-12,513	30,8	79,4
C. kamancamarai	FD4	11,295	-12,513	28,2	86,9
C. kamancamarai	FD44	11,295	-12,513	38,9	80,8
C. kamancamarai	FD45	11,295	-12,513	26,6	106,3
C. kamancamarai	FD48	11,295	-12,513	22,4	77,6
C. kamancamarai	GN11_130	10,341	-12,171	33,5	158
C. kamancamarai	GN11_140	10,341	-12,171	28	101,9
C. kamancamarai	GN11_176	10,297	-11,942	31,2	93,1
C. kamancamarai	GN11_179	10,297	-11,942	28,9	126,5
C. kamancamarai	GN11_184	10,297	-11,942	28	163,6
C. kamancamarai	GN11_188	10,297	-11,942	27,8	189,8
C. kamancamarai	KD061	10,844	-13,809	23,9	94,8
C. kamancamarai	KD062	10,844	-13,809	28	87,2
C. kamancamarai	KD063	10,844	-13,809	22,4	76,4
C. kamancamarai	KD069	10,835	-13,742	28,6	85
C. kamancamarai	KD070	10,835	-13,742	22,1	71,7
C. kamancamarai	KD071	10,855	-13,742	22,7	100,3
C. kamancamarai	KD076	10,865	-13,741	20,8	129,2
C. kamancamarai	KD077	10,865	-13,741	24,3	164
C. kamancamarai	KD078	10,865	-13,741	19,5	153
C. kamancamarai	KD079	10,865	-13,741	20,1	144,3
C. kamancamarai	KD081	10,877	-13,72	26,7	103
C. kamancamarai	KD084	10,958	-13,711	25,9	104,5
C. kamancamarai	KD092	10,974	-13,708	23,9	178,6
C. kamancamarai	KD124	11,075	-13,671	22,5	57,8
C. kamancamarai	KD125	11,075	-13,671	22,6	113,9
C. kamancamarai	KD126	11,075	-13,671	24,6	111,6

MOLECULAR ECOLOGY

Table S5: Sample information for the dataset comprising samples of *C. kamancamarai*. Details include sample ID, sampling locality (latitude and longitude), species assignment, and sequencing statistics: frequency of missing data per sample in the final VCF's and mean coverage per site based on the BAM files.

Species	Sample ID	Latitude	Longitude	Freq. missing data (% loci)	Mean coverage
C. kamancamarai	CB2010_001	11,296	-12,507	24,6	76,2
C. kamancamarai	CB2010_002	11,296	-12,507	23,8	100,8
C. kamancamarai	CB2010_016	11,296	-12,513	28,1	112,1
C. kamancamarai	CB2010_017	11,296	-12,513	32,5	94,3
C. kamancamarai	CB2010_030	11,287	-12,504	24,1	108,7
C. kamancamarai	CB2010_044	11,192	-12,316	27,6	102,6
C. kamancamarai	CB2010_055	10,851	-12,521	35,3	93,6
C. kamancamarai	CB2010_056	10,851	-12,521	31,6	102,8
C. kamancamarai	CB2010_057	10,851	-12,521	29,3	93,4
C. kamancamarai	CB2010_059	10,851	-12,521	26,4	120,8
C. kamancamarai	CB2010_061	10,851	-12,521	30,3	91,7
C. kamancamarai	CB2010_082	10,84	-12,556	25,5	212,4
C. kamancamarai	CB2010_088	10,82	-12,192	32,9	100,3
C. kamancamarai	CB2010_089	10,82	-12,192	31,1	103,9
C. kamancamarai	CB2010_090	10,82	-12,192	29,6	117,5
C. kamancamarai	CB2010_091	10,82	-12,192	30,1	111,7
C. kamancamarai	FD27	11,295	-12,513	28	79,6
C. kamancamarai	FD4	11,295	-12,513	25,7	87,6
C. kamancamarai	FD44	11,295	-12,513	36,6	81,2
C. kamancamarai	FD45	11,295	-12,513	26,4	106,2
C. kamancamarai	FD48	11,295	-12,513	25,6	77,4
C. kamancamarai	GN11_130	10,341	-12,171	35,1	158,6
C. kamancamarai	GN11_140	10,341	-12,171	30,3	103,1
C. kamancamarai	GN11_176	10,297	-11,942	32,5	96,2
C. kamancamarai	GN11_179	10,297	-11,942	30,6	128,8
C. kamancamarai	GN11_184	10,297	-11,942	30,3	167,8
C. kamancamarai	GN11_188	10,297	-11,942	30,2	195
C. kamancamarai	KD061	10,844	-13,809	23,8	95,6
C. kamancamarai	KD062	10,844	-13,809	25,5	88,3
C. kamancamarai	KD063	10,844	-13,809	20,4	77,2
C. kamancamarai	KD069	10,835	-13,742	24,7	85,9
C. kamancamarai	KD070	10,835	-13,742	20,9	72,2
C. kamancamarai	KD071	10,855	-13,742	20,1	101,3
C. kamancamarai	KD076	10,865	-13,741	20,7	130,6
C. kamancamarai	KD077	10,865	-13,741	20,8	166,1
C. kamancamarai	KD078	10,865	-13,741	20,2	154,6
C. kamancamarai	KD079	10,865	-13,741	20,9	144,8
C. kamancamarai	KD081	10,877	-13,72	23,1	104,2
C. kamancamarai	KD084	10,958	-13,711	24,9	105,5
C. kamancamarai	KD092	10,974	-13,708	21,7	179,9
C. kamancamarai	KD124	11,075	-13,671	20,7	58,4
C. kamancamarai	KD125	11,075	-13,671	21,4	115,3
C. kamancamarai	KD126	11,075	-13,671	21,6	113,2

Table S6: Sample information for the dataset comprising samples of *C. alleni*. Details include sample ID, sampling locality (latitude and longitude), species assignment, and sequencing statistics: frequency of missing data per sample in the final VCF's and mean coverage per site based on the BAM files.

Species	Sample ID	Latitude	Longitude	Freq. missing data (% loci)	Mean coverage
C. alleni	CB2010_180	8,351	-9,417	27,7	84,5
C. alleni	CB2010_181	8,351	-9,417	21,9	112,7
C. alleni	CB2010_182	8,351	-9,417	32,4	85,2
C. alleni	CB2010_183	8,351	-9,417	21,1	96,7
C. alleni	FO_5	8,531	-8,906	41,6	63,7
C. alleni	GN11_436	7,697	-8,396	21,4	117,7
C. alleni	GN11_439	7,712	-8,395	21	144,7
C. alleni	GN11_442	7,674	-8,375	24,6	105,1
C. alleni	GN11_443	7,646	-8,336	22,2	121
C. alleni	GN11_446	7,649	-8,424	22,2	93,1
C. alleni	GN11_447	7,649	-8,424	21,4	146,9
C. alleni	GN11_448	7,631	-8,414	21,6	136,7
C. alleni	GN11_453	7,631	-8,414	23,6	114,3
C. alleni	GN11_454	7,631	-8,414	31,5	77
C. alleni	GN11_457	7,633	-8,352	22,3	112,7
C. alleni	GN11_458	7,67	-8,354	22,6	135,3
C. alleni	GN11_459	7,633	-8,352	26,5	84,8
C. alleni	GN11_462	7,647	-8,368	19,8	102,1
C. alleni	GN11_463	7,647	-8,368	19,2	151,7
C. alleni	GN11_466	7,647	-8,368	18,9	163,9
C. alleni	GN11_469	7,615	-8,45	18,9	134,9
C. alleni	GN11_470	7,615	-8,45	20,5	116,1
C. alleni	GN11_473	7,624	-8,418	22,9	97,1
C. alleni	GN11_474	7,624	-8,418	19,8	125,6
C. alleni	GN11_478	7,624	-8,418	19,3	131,2
C. alleni	GN11_482	7,67	-8,374	21	148,2
C. alleni	GN11_483	7,677	-8,385	20,9	144,7
C. alleni	GN11_484	7,593	-8,468	20,7	151
C. alleni	GN11_485	7,593	-8,468	21,1	135
C. alleni	GN11_489	7,671	-8,395	21,3	113,5
C. alleni	GN11_490	7,671	-8,395	20,7	152,6
C. alleni	GN11_493	7,677	-8,385	20,1	115,9
C. alleni	GN11_494	7,677	-8,385	19,4	111,7
C. alleni	GO20	7,453	-10,692	51,8	45,5
C. alleni	GOL12	7,597	-11,023	60,1	22,6
C. alleni	GRE39	5,405	-7,73	47	71,2
C. alleni	GRE44	5,405	-7,73	60,2	58,1
C. alleni	GS096	7,658	-10,903	46,1	44,4
C. alleni	GS111	7,658	-10,905	21,5	111,3
C. alleni	GS112	7,658	-10,905	21,5	131
C. alleni	GS113	7,658	-10,905	21,5	120,3
C. alleni	GS114	7,658	-10,905	21,3	111,4
C. alleni	GS90	7,658	-10,903	30,8	91,5
C. alleni	JP0142	8,501	-11,147	29	108,3
C. alleni	JP0143	8,501	-11,147	39,4	60,4
C. alleni	JP7_122	8,354	-9,416	23,3	126,6
C. alleni	JP7_123	8,354	-9,416	23	111,3
C. alleni	JP7_154	8,554	-8,888	23,1	107,1
C. alleni	JP7_191	8,365	-8,433	31	98,9
C. alleni	LI006	5,662	-8,161	45,3	115,4
C. alleni	LI007	5,662	-8,161	45,6	131,7
C. alleni	LI008	5,662	-8,161	46,9	77
C. alleni	LI009	5,662	-8,161	46,7	77,7

C. alleni	LI10_032	7,458	-8,666	21,2	125,6
C. alleni	LI10_034	7,445	-8,658	28,5	109,2
C. alleni	LI10_068	7,441	-8,593	23	119,9
C. alleni	LI10_117	7,484	-8,577	25,1	100,5
C. alleni	LI10_122	7,441	-8,593	24,9	108,7
C. alleni	LI10_138	7,483	-8,537	26,6	104,8
C. alleni	LI157	5,688	-8,215	45,4	157,3
C. alleni	LOM107	9,224	-11,159	55,2	43
C. alleni	LOM52	9,213	-11,144	39	97,9
C. alleni	LOM55	9,213	-11,144	29	101,6
C. alleni	LOM66	9,209	-11,173	28,6	98,4
C. alleni	LOM67	9,209	-11,173	30,8	99,4
C. alleni	LOM89	9,213	-11,144	30,3	89,3
C. alleni	LOM90	9,213	-11,144	30,4	110,3
C. alleni	MH0065	7,596	-8,263	25,6	75,6
C. alleni	MH0081	7,544	-8,838	23,4	79,6
C. alleni	MH0082	7,544	-8,838	21	135,6
C. alleni	MH0096	8,283	-8,738	22,6	119,6
C. alleni	MH0108	8,334	-8,709	26	122
C. alleni	MH0124	8,318	-8,713	23,8	144,8
C. alleni	MH0125	8,318	-8,713	25,4	128,5
C. alleni	MH0126	8,815	-8,862	26,3	117
C. alleni	MH0127	8,815	-8,862	35,2	95,7
C. alleni	MH0128	8,815	-8,862	23,1	101,4
C. alleni	MH0140	8,884	-8,294	25,3	121,1
C. alleni	MH0141	8,884	-8,294	22,7	127,9
C. alleni	MTN190	7,59	-8,452	20,9	135,7
C. alleni	MTN208	7,59	-8,452	21,1	122,3
C. alleni	MTN5	7,714	-8,362	56,1	74,5
C. alleni	NIM121	8,511	-11,102	43,8	60,7
C. alleni	NIM87	8,501	-11,147	28,6	117,6
C. alleni	NIM95	8,499	-11,151	29,8	113,6
C. alleni	NIM98	8,499	-11,151	31,4	81,7
C. alleni	P_LI_12_409	6,443	-9,056	46,4	111,4
C. alleni	P_LI_12_458	5,643	-8,208	45,4	116,9
C. alleni	P_LI_12_480	5,647	-8,207	45,8	85,6
C. alleni	PB_11_10	8,043	-9,076	21,5	109,5
C. alleni	PB_11_104	8,043	-9,076	21,9	107
C. alleni	PB_11_11	8,043	-9,076	25,2	76,7
C. alleni	PB_11_267	7,235	-9,31	23,5	106,3
C. alleni	PB_11_40	8,043	-9,076	25,7	113,1
C. alleni	PB_11_459	9,208	-8,926	24,2	95,5
C. alleni	PB_11_462	9,21	-8,927	25,7	76,9
C. alleni	PB_11_474	9,263	-8,931	24,9	100,3
C. alleni	PB_11_584	9,252	-8,874	25,5	96,8
C. alleni	PB_11_9	8,043	-9,076	23,8	102,1
C. alleni	PB_11_924	9,262	-8,928	22,9	123,9
C. alleni	PG_L_13_066	5,074	-8,545	46,1	101,1
C. alleni	PG_L_13_067	5,077	-8,54	46,2	99,7
C. alleni	PG_L_13_174	5,082	-8,501	47,7	77,8
C. alleni	PLI_12_277	5,52	-8,345	45,9	105,7
C. alleni	SI21	9,28	-9,114	22,2	121,7
C. alleni	SI25	9,28	-9,114	29,8	92,6
C. alleni	SI41	9,161	-8,933	25,4	83,4
C. alleni	SI69	8,889	-8,616	23,6	59,6
C. alleni	SI78	8,881	-8,295	35,1	56,6
C. alleni	SSF_001	6,234	-2,69	62,1	59,5
C. alleni	SSF_002	6,236	-2,695	59,3	59,4
C. alleni	SSF_003	6,238	-2,696	59,7	60,7

C. alleni	SSF_005	6,236	-2,696	60,3	65
C. alleni	TI10	8,89	-10,79	31,6	60,7
C. alleni	TI11	8,89	-10,79	28,7	101,5
C. alleni	TI17	8,89	-10,79	28,4	92,8
C. alleni	TI62	8,89	-10,79	29,2	99
C. alleni	TI63	8,89	-10,79	31,2	75,6
C. alleni	TI66	8,865	-10,792	30,1	79,6
C. alleni	TI67	8,867	-10,792	31,9	93,6

Table S7: Sample information for the dataset comprising samples of *C. sagyimase*. Details include sample ID, sampling locality (latitude and longitude), species assignment, and sequencing statistics: frequency of missing data per sample in the final VCF's and mean coverage per site based on the BAM files.

Species	Sample ID	Latitude	Longitude	Freq. missing data (% loci)	Mean coverage
C. sagyimase	155	6,262	-0,555	13,8	103,4
C. sagyimase	212	6,271	-0,565	13	76,3
C. sagyimase	213	6,271	-0,565	5,8	75,3
C. sagyimase	217	6,271	-0,565	9,4	69,2
C. sagyimase	218	6,271	-0,565	6,1	92,9
C. sagyimase	AT01	6,207	-0,577	4,9	85,5
C. sagyimase	AT02	6,207	-0,577	5,8	78,6
C. sagyimase	AT04	6,207	-0,577	7,4	79,8
C. sagyimase	AT09	6,207	-0,577	9,5	64,1
C. sagyimase	AT11	6,207	-0,577	6,6	69,6
C. sagyimase	AT13	6,207	-0,577	19,9	88,2
C. sagyimase	AT20	6,207	-0,577	5,2	80,8

Table S8: Sample information for the dataset comprising samples of *C. derooi*. Details include sample ID, sampling locality (latitude and longitude), species assignment, and sequencing statistics: frequency of missing data per sample in the final VCF's and mean coverage per site based on the BAM files.

Species	Sample ID	Latitude	Longitude	Freq. missing data (% loci)	Mean coverage
C. derooi	AM01	6,85	0,446	12,9	112,1
C. derooi	AM03	6,85	0,446	22,9	102,7
C. derooi	AM06	6,85	0,446	12,3	87
C. derooi	AM07	6,85	0,446	13,7	57,1
C. derooi	AM08	6,85	0,446	16,8	96,7
C. derooi	AM09	6,85	0,446	16,3	74,8
C. derooi	AM12	6,85	0,446	8,7	101,5
C. derooi	AM18	6,85	0,446	8,3	84,7
C. derooi	AM55	6,845	0,438	27,8	91,4
C. derooi	Anedi_1	6,933	0,566	3,3	85,6
C. derooi	Anedi_2	6,933	0,566	2,4	77,8
C. derooi	Anedi_3	6,933	0,566	3,2	77,9
C. derooi	Anedi_4	6,933	0,566	4,1	69,5
C. derooi	Anedi_5	6,933	0,566	4,3	66
C. derooi	Douane_1	6,948	0,575	6,7	61,1
C. derooi	Douane_3	6,948	0,575	7,7	58
C. derooi	Douane_4	6,948	0,575	6,5	55,2
C. derooi	Douane_5	6,948	0,575	7,8	50,9
C. derooi	Kamalo_1	6,951	0,598	2,4	74,2
C. derooi	Kamalo_2	6,951	0,598	7,6	51,8
C. derooi	Kamalo_3	6,951	0,598	3,8	60,8
C. derooi	Kamalo_4	6,951	0,598	6,1	56,7
C. derooi	Kamalo_5	6,951	0,598	3,6	63,1
C. derooi	Kamalo_6	6,951	0,598	2,8	67,3

C. derooi	TOG76	6,952	0,565	25,1	87,4
C. derooi	TOG78	6,952	0,565	6,9	77,8
C. derooi	TOG79	6,952	0,565	6,2	74,5
C. derooi	TOG80	6,952	0,565	17,7	68,9
C. derooi	TOG81	6,952	0,565	20	68
C. derooi	TOG86	6,952	0,565	12,1	84
C. derooi	Yikpa_1	7,128	0,601	4,7	72,6
C. derooi	Yikpa_2	7,128	0,601	6,6	54,2
C. derooi	Yikpa_3	7,128	0,601	5,9	58
C. derooi	Yikpa_4	7,128	0,601	5,5	55
C. derooi	Yikpa_5	7,128	0,601	17,4	52,4
C. derooi	Zoto_1	6,943	0,575	8,1	51
C. derooi	Zoto_2	6,943	0,575	4,9	53,8
C. derooi	Zoto_3	6,943	0,575	6,5	57,4
C. derooi	Zoto_4	6,943	0,575	5,7	73,3
C. derooi	Zoto_5	6,943	0,575	33,7	54,6

Table S9: Overview of samples included in the population-based analyses for calculating the summary statistics and for demographic inference with ∂a∂i. Sample selection was based on low levels of admixture to minimize bias in demographic inference. For ∂a∂i analyses, only populations with more than 10 individuals were included. For estimating Tajima's D with PopGenome, we only excluded Kam_4, due to its small sample size (n = 2).

Species	Population	Sample size	Sample IDs
C. alleni	All_1	11	GN11_462, GN11_463, GN11_466, GN11_469, GN11_470, GN11_473, GN11_474, GN11_478, GN11_493, GN11_494, MH0065
C. alleni	All_2	26	GN11_436, GN11_439, GN11_442, GN11_443, GN11_446, GN11_447, GN11_448, GN11_453, GN11_454, GN11_457, GN11_458, GN11_459, GN11_482, GN11_483, GN11_484, GN11_485, GN11_489, GN11_490, LI10_032, LI10_034, LI10_068, LI10_117, LI10_122, LI10_138, MTN190, MTN208
C. alleni	All_3	32	CB2010_180, CB2010_181, CB2010_183, FO_5, JP7_122, JP7_123, JP7_154, JP7_191, MH0096, MH0108, MH0124, MH0125, MH0126, MH0127, MH0128, MH0140, MH0141, PB_11_10, PB_11_104, PB_11_11, PB_11_40, PB_11_459, PB_11_462, PB_11_474, PB_11_584, PB_11_9, PB_11_924, SI21, SI25, SI41, SI69, SI78
C. alleni	All_4	14	GRE39, GRE44, LI006, LI007, LI008, LI009, LI157, P_LI_12_409, P_LI_12_458, P_LI_12_480, PG_L_13_066, PG_L_13_067, PG_L_13_174, PLI_12_277
C. alleni	All_5	20	JP0142, JP0143, LOM107, LOM52, LOM55, LOM66, LOM67, LOM89, LOM90, NIM121, NIM87, NIM95, NIM98, TI10, TI11, TI17, TI62, TI63, TI66, TI67
C. alleni	All_6	4	SSF_001, SSF_002, SSF_003, SSF_005
C. kamancamarai	Kam_1	4	CB2010_088, CB2010_089, CB2010_090, CB2010_091
C. kamancamarai	Kam_2	11	CB2010_001, CB2010_002, CB2010_016, CB2010_017, CB2010_030, CB2010_044, FD27, FD4, FD44, FD45, FD48
C. kamancamarai	Kam_3	6	CB2010_055, CB2010_056, CB2010_057, CB2010_059, CB2010_061, CB2010_082
C. kamancamarai	Kam_4	2	GN11_130, GN11_140
C. kamancamarai	Kam_5	16	KD061, KD062, KD063, KD069, KD070, KD071, KD076, KD077, KD078, KD079, KD081, KD084, KD092, KD124, KD125, KD126
C. kamancamarai	Kam_6	4	GN11_176, GN11_179, GN11_184, GN11_188
C. derooi	Der_1	8	AM01, AM03, AM06, AM08, AM09, AM12, AM18, AM55
C. derooi	Der_2	25	Anedi_1, Anedi_2, Anedi_3, Anedi_4, Anedi_5, Douane_1, Douane_3, Douane_4, Douane_5, Kamalo_1, Kamalo_2, Kamalo_3, Kamalo_4, Kamalo_5, Kamalo_6, TOG76, TOG78, TOG79, TOG80, TOG81, TOG86, Zoto_1, Zoto_2, Zoto_3, Zoto_4
C. derooi	Der_3	5	Yikpa_1, Yikpa_2, Yikpa_3, Yikpa_4, Yikpa_5
C. sagyimase	Sag_1	12	155, 212, 213, 217, 218, AT01, AT02, AT04, AT09, AT11, AT13, AT20

MOLECULAR ECOLOGY

Table S10: Neutrality statistics for investigated *Conraua* populations (Tajima's D, Fu and Li's F, Fu and Li's D). Significance of Tajima's D was determined by comparing observed values to a neutral distribution generated from 1,000 simulations, with p-values indicating significant deviations from neutrality (* p < 0.05; ** p < 0.10).**

Species	Population ID	Number of sites	Tajima's D	p-value	Significant	Fu & Li's F	Fu & Li's D
C. alleni	All_1	6.689	-0,4003	0,3870		-0,5215	-0,5639
C. alleni	All_2	6.814	-0,9286	0,1850		-1,1801	-1,0841
C. alleni	All_3	9.302	-1,4372	0,0350	***	-2,1299	-2,0694
C. alleni	All_4	3.534	-0,4379	0,3670		-0,8717	-0,9875
C. alleni	All_5	8.072	-1,1275	0,0950	**	-1,7917	-1,8113
C. alleni	All_6	605	0,7500	0,2050		0,8659	0,6772
C. derooi	Der_1	4.532	0,7398	0,1610		0,6579	0,4160
C. derooi	Der_2	2.278	0,7019	0,1860		0,5286	0,2405
C. derooi	Der_3	2.489	1,0727	0,1040		1,0112	0,7414
C. kamancamarai	Kam_1	1.725	0,3083	0,2210		0,4736	0,2845
C. kamancamarai	Kam_2	2.309	-0,4370	0,3680		-0,5883	-0,6453
C. kamancamarai	Kam_3	5.993	-0,7619	0,2210		-0,7413	-0,8185
C. kamancamarai	Kam_4	3.116	-0,0814	0,3370		0,4444	-0,0814
C. kamancamarai	Kam_5	1.479	0,0497	0,4010		0,2386	0,2239
C. kamancamarai	Kam_6	6.271	0,7837	0,1550		0,8281	0,5926
C. sagyimase	Sag_1	1.422	-0,0910	0,4820		-0,0980	-0,1660

Table S11: Nucleotide diversity statistics for investigated *Conraua* populations. *C. kamancamarai* species present comparatively high nucleotide diversity values.

Species	Population ID	Nucleotide diversity (π)
C. alleni	*All_1*	0,0073
C. alleni	*All_2*	0,0075
C. alleni	*All_3*	0,0060
C. alleni	*All_4*	0,0070
C. alleni	*All_5*	0,0062
C. alleni	*All_6*	0,0107
C. derooi	*Der_1*	0,0070
C. derooi	*Der_2*	0,0098
C. derooi	*Der_3*	0,0067
C. kamancamarai	*Kam_1*	0,0129
C. kamancamarai	*Kam_2*	0,0122
C. kamancamarai	*Kam_3*	0,0077
C. kamancamarai	*Kam_4*	0,0086
C. kamancamarai	*Kam_5*	0,0162
C. kamancamarai	*Kam_6*	0,0088
C. sagyimase	*Sag_1*	0,0125

MOLECULAR ECOLOGY

Table S12: Pairwise Fst values between populations across and within species. This heatmap illustrates the genetic differentiation (Fst) values for each population pair. Populations within the same species generally exhibit lower Fst values compared to populations from different species, reflecting closer genetic relatedness within species. Population comparisons within *C. kamanamcarai*, show more elevated levels of differentiation compared to population comparisons within *C. alleni* or *C. derooi*.

	All_1	*All_2*	*All_3*	*All_4*	*All_5*	*All_6*	*Der_1*	*Der_2*	*Der_3*	*Kam_1*	*Kam_2*	*Kam_3*	*Kam_4*	*Kam_5*	*Kam_6*	*Sag_1*
All_1	NA	0,19	0,16	0,29	0,22	0,38	0,63	0,66	0,66	0,60	0,50	0,57	0,61	0,51	0,62	0,56
All_2	0,19	NA	0,09	0,27	0,21	0,33	0,58	0,60	0,60	0,55	0,47	0,53	0,55	0,47	0,56	0,53
All_3	0,16	0,09	NA	0,18	0,15	0,20	0,44	0,47	0,46	0,41	0,38	0,40	0,40	0,38	0,42	0,41
All_4	0,29	0,27	0,18	NA	0,24	0,41	0,66	0,69	0,70	0,63	0,51	0,60	0,65	0,51	0,66	0,59
All_5	0,22	0,21	0,15	0,24	NA	0,26	0,49	0,53	0,51	0,46	0,41	0,45	0,46	0,41	0,47	0,46
All_6	0,38	0,33	0,20	0,41	0,26	NA	0,82	0,83	0,89	0,81	0,57	0,74	0,85	0,57	0,86	0,69
Der_1	0,63	0,58	0,44	0,66	0,49	0,82	NA	0,21	0,25	0,77	0,57	0,72	0,78	0,58	0,81	0,59
Der_2	0,66	0,60	0,47	0,69	0,53	0,83	0,21	NA	0,21	0,80	0,63	0,76	0,82	0,62	0,82	0,62
Der_3	0,66	0,60	0,46	0,70	0,51	0,89	0,25	0,21	NA	0,83	0,61	0,78	0,86	0,61	0,88	0,63
Kam_1	0,60	0,55	0,41	0,63	0,46	0,81	0,77	0,80	0,83	NA	0,37	0,51	0,64	0,40	0,65	0,66
Kam_2	0,50	0,47	0,38	0,51	0,41	0,57	0,57	0,63	0,61	0,37	NA	0,38	0,28	0,25	0,42	0,53
Kam_3	0,57	0,53	0,40	0,60	0,45	0,74	0,72	0,76	0,78	0,51	0,38	NA	0,58	0,41	0,55	0,63
Kam_4	0,61	0,55	0,40	0,65	0,46	0,85	0,78	0,82	0,86	0,64	0,28	0,58	NA	0,35	0,72	0,67
Kam_5	0,51	0,47	0,38	0,51	0,41	0,57	0,58	0,62	0,61	0,40	0,25	0,41	0,35	NA	0,45	0,54
Kam_6	0,62	0,56	0,42	0,66	0,47	0,86	0,81	0,82	0,88	0,65	0,42	0,55	0,72	0,45	NA	0,69
Sag_1	0,56	0,53	0,41	0,59	0,46	0,69	0,59	0,62	0,63	0,66	0,53	0,63	0,67	0,54	0,69	NA

MOLECULAR ECOLOGY

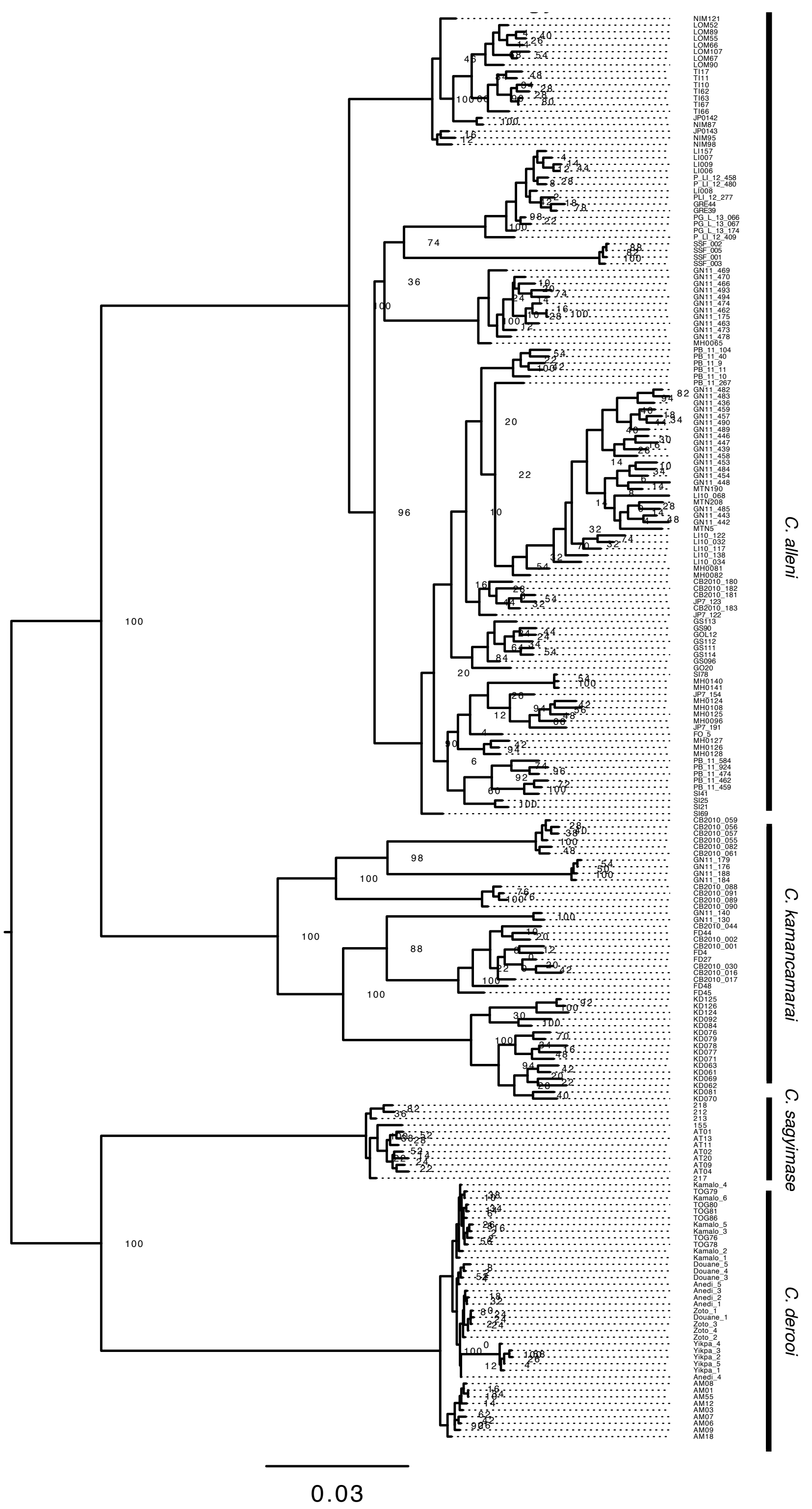

Figure S1. Phylogenetic tree for all investigated *Conraua* species inferred based on a concatenated SNP dataset.

MOLECULAR ECOLOGY

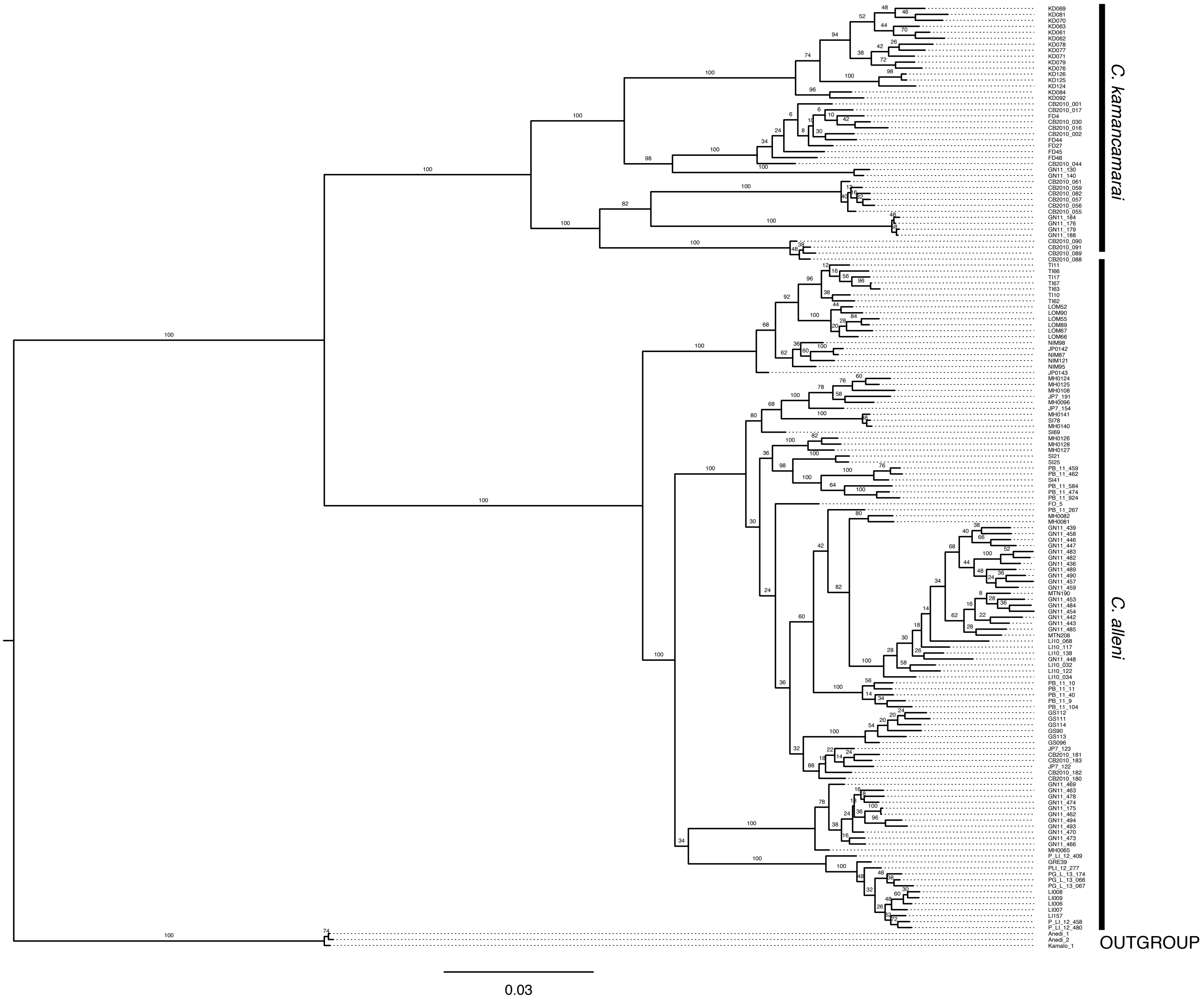

Figure S2. Phylogenetic tree for *C. kamancamarai* and *C. alleni* inferred based on a concatenated SNP dataset.

MOLECULAR ECOLOGY

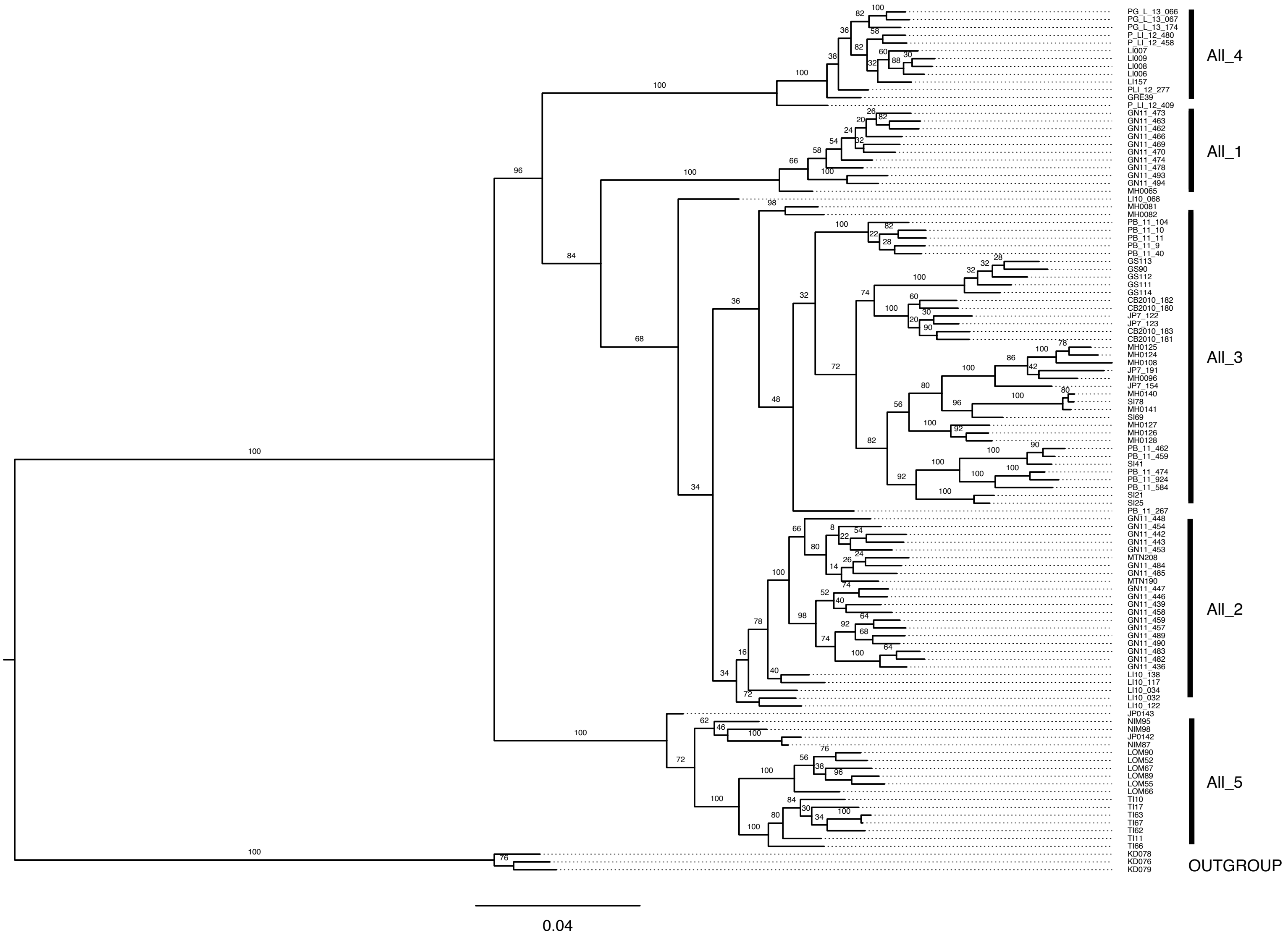

Figure S3. Phylogenetic tree for *C. alleni* inferred based on a concatenated SNP dataset.

MOLECULAR ECOLOGY

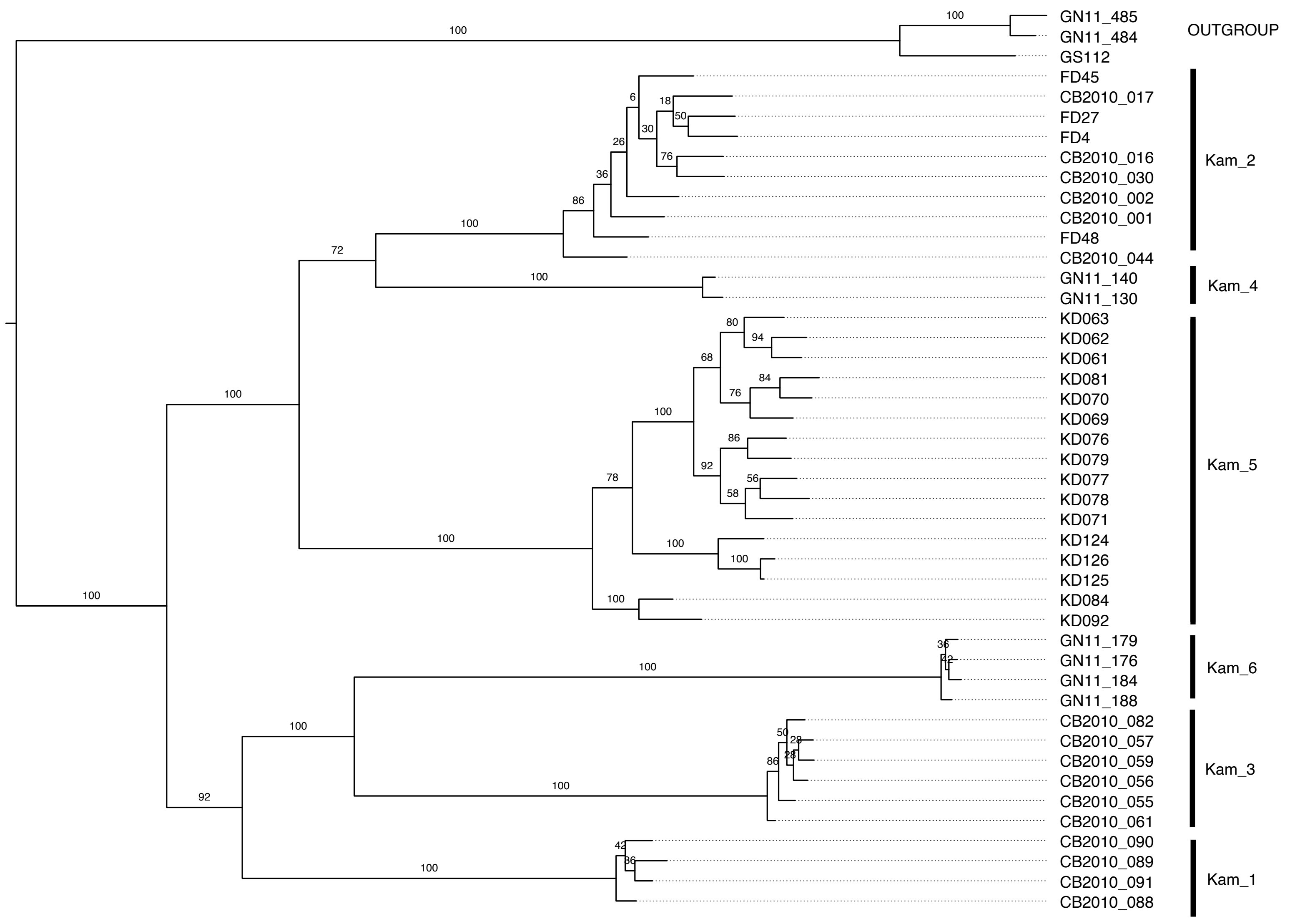

Figure S4. Phylogenetic tree for *C. kamancamarai* inferred based on a concatenated SNP dataset.

MOLECULAR ECOLOGY

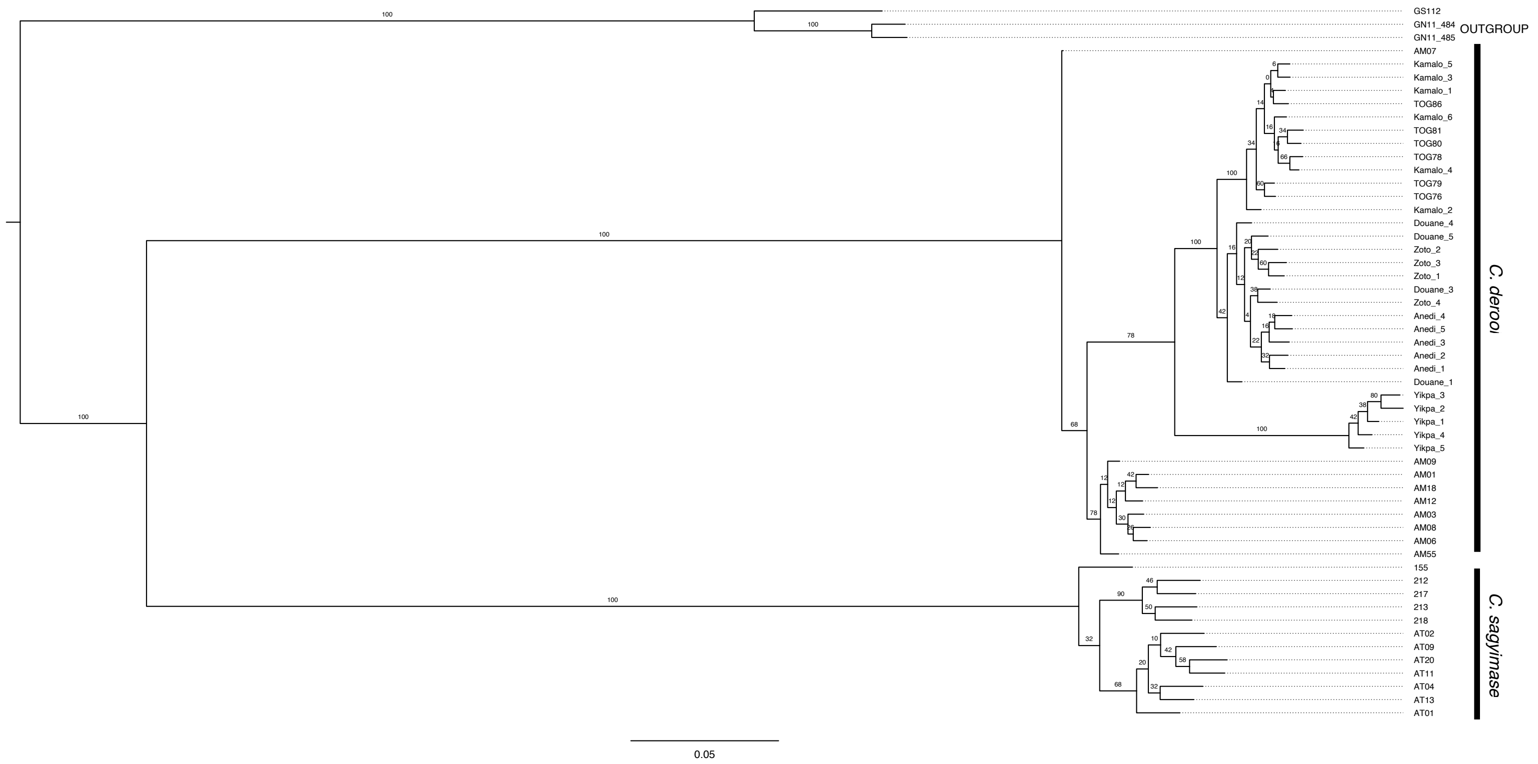

Figure S5. Phylogenetic tree for *C. derooi* and *C. sagyimase* inferred based on a concatenated SNP dataset.

MOLECULAR ECOLOGY

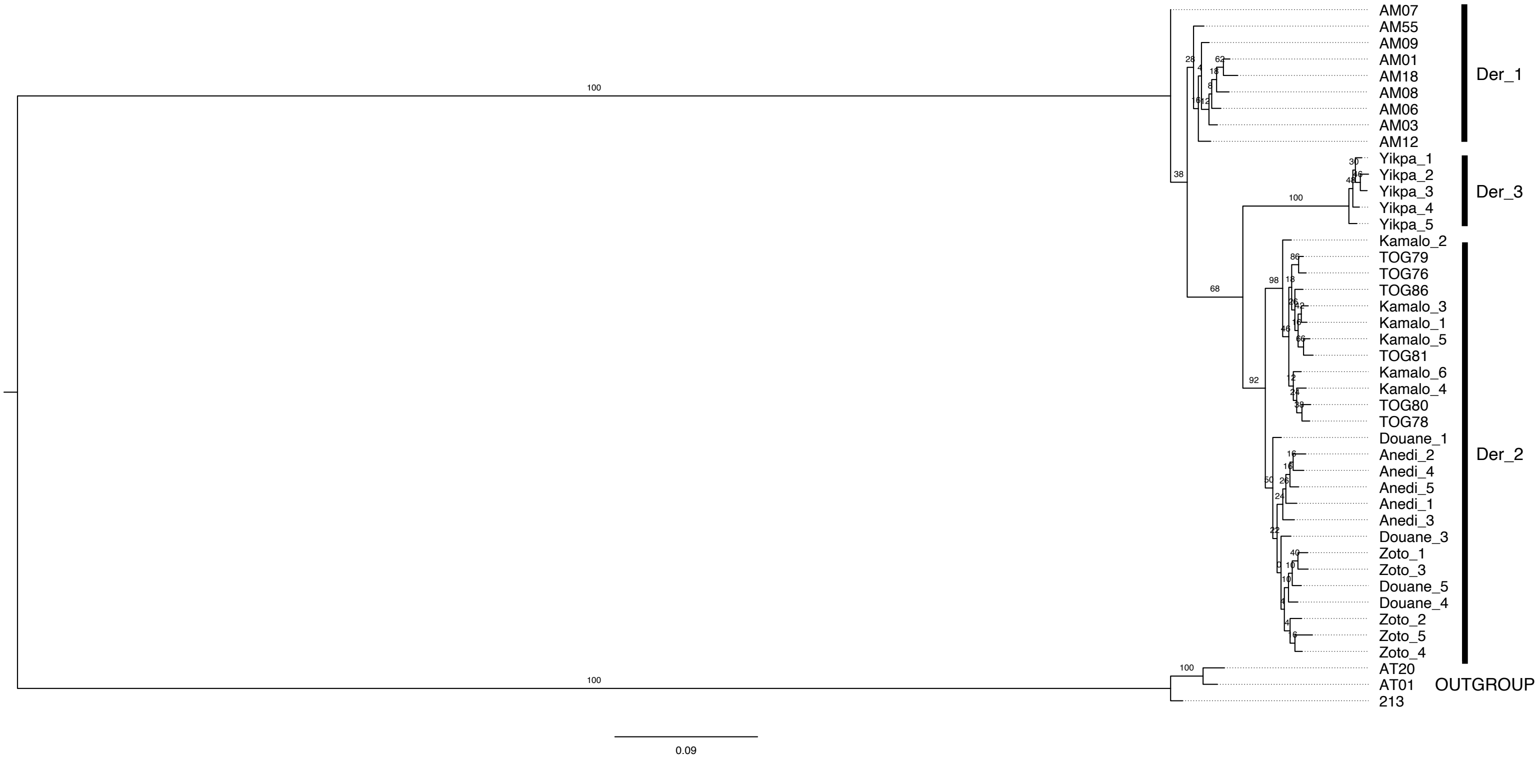

Figure S6. Phylogenetic tree for *C. derooi* inferred based on a concatenated SNP dataset.

MOLECULAR ECOLOGY

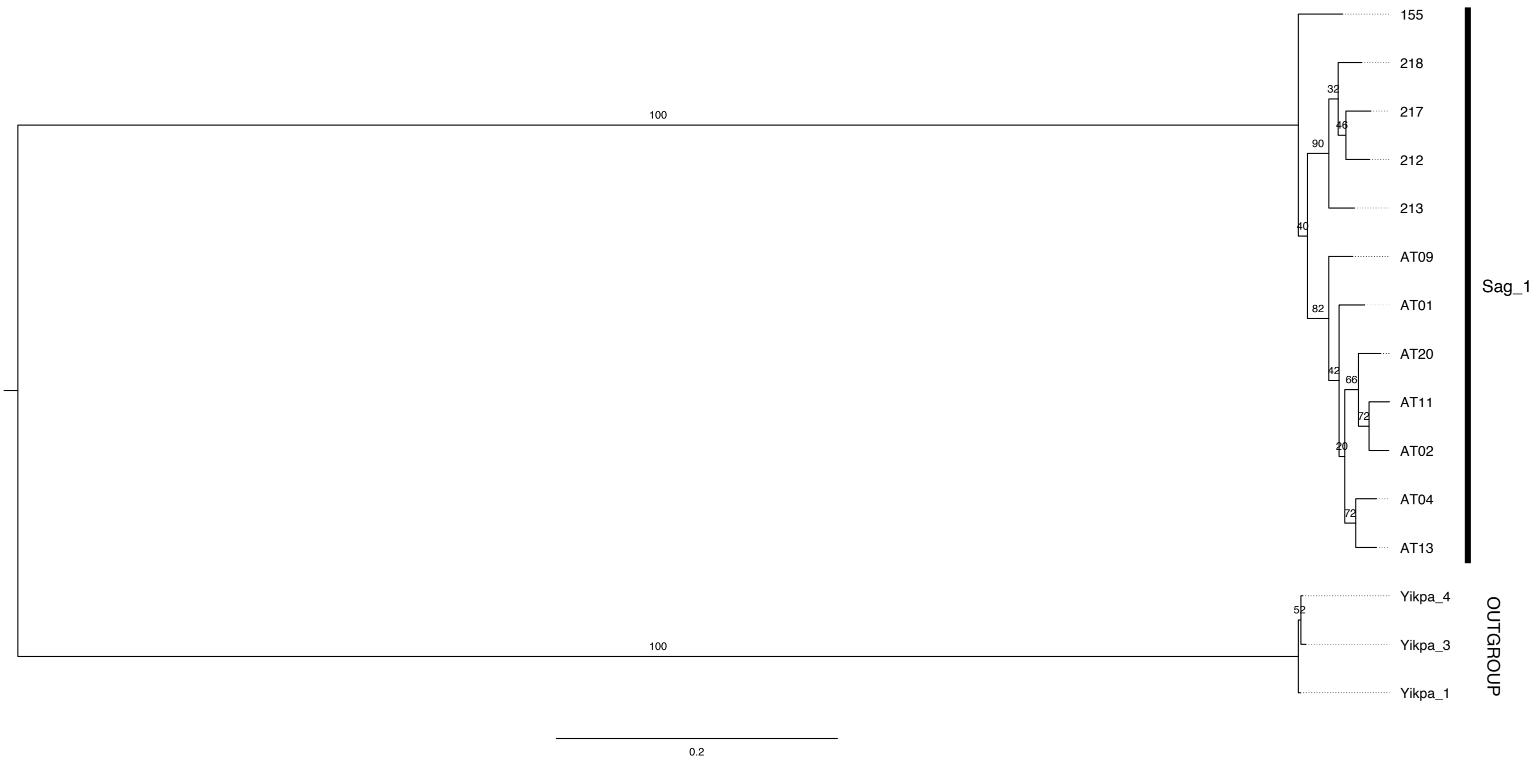

Figure S7. Phylogenetic tree for *C. sagyimase* inferred based on a concatenated SNP dataset.

MOLECULAR ECOLOGY

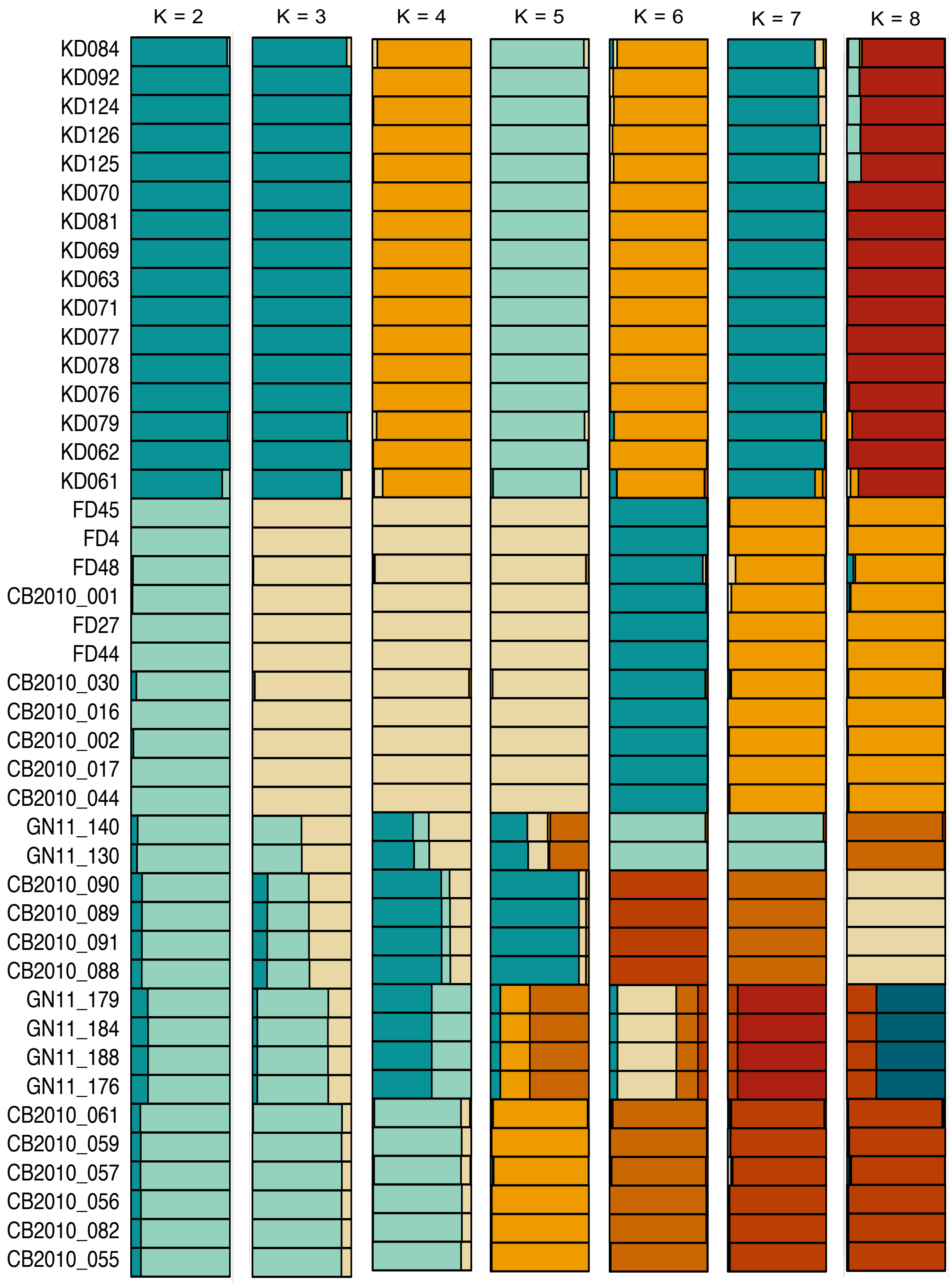

Figure S8. Structure plots for *C. kamancamarai* displaying genetic clusters across K-values ranging from 2 to 8. Each horizontal bar represents an individual with colours indicating cluster assignment based on admixture proportions. Sample IDs are listed in the left column. K values are indicated in the top row.

MOLECULAR ECOLOGY

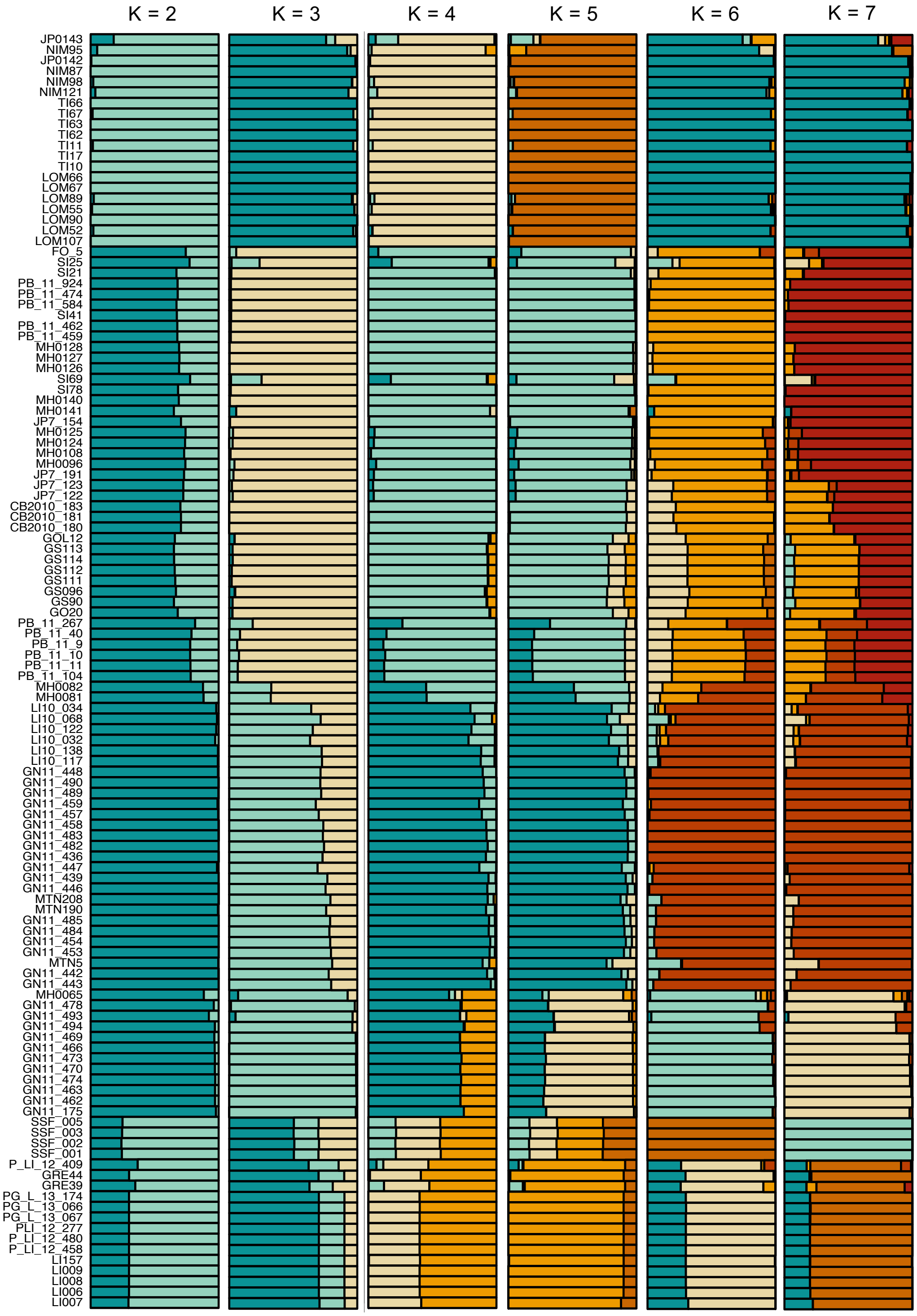

Figure S9. Structure plots for *C. alleni* displaying genetic clusters across K-values ranging from 2 to 7. Each horizontal bar represents an individual with colours indicating cluster assignment based on admixture proportions. Sample IDs are listed in the left column. K values are indicated in the top row.

MOLECULAR ECOLOGY

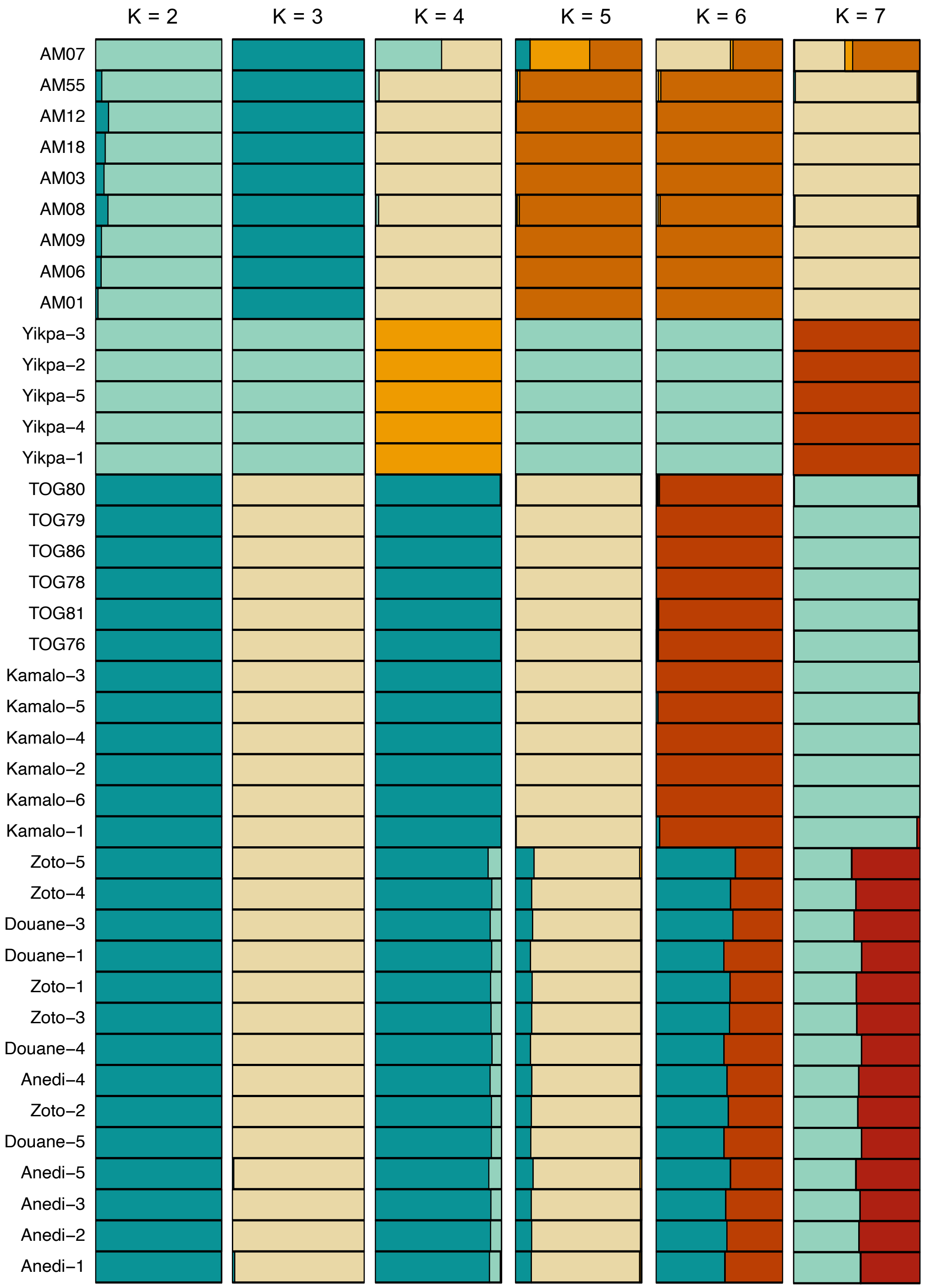

Figure S10. Structure plots for *C. derooi* displaying genetic clusters across K-values ranging from 2 to 7. Each horizontal bar represents an individual with colours indicating cluster assignment based on admixture proportions. Sample IDs are listed in the left column. K values are indicated in the top row.

MOLECULAR ECOLOGY

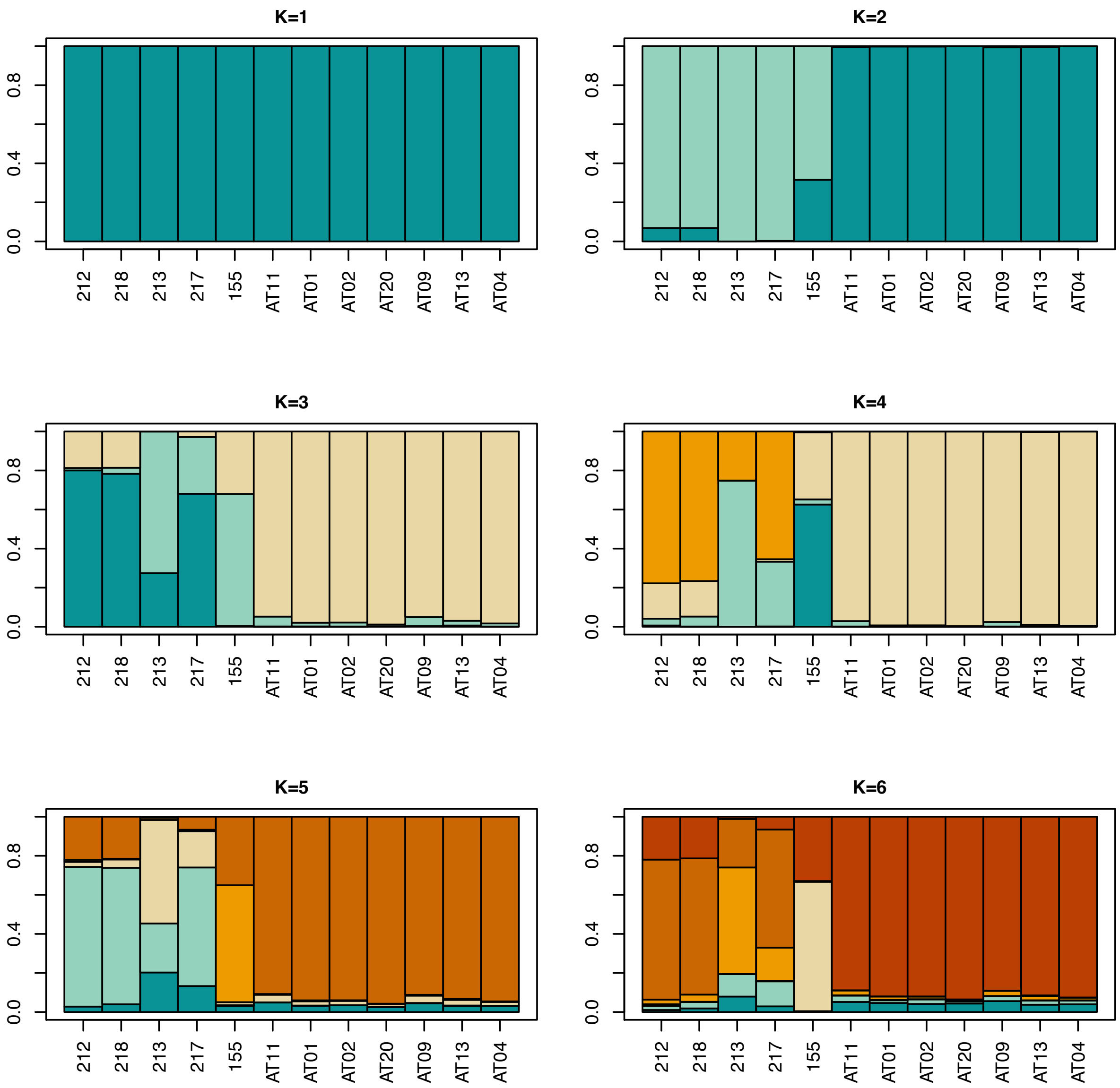

Figure S11. Structure plots for *C. sagyimase* displaying genetic clusters across K-values ranging from 2 to 6. Vertical bars represent distinct individuals with admixture proportions coloured according to cluster assignment. Sample IDs are listed below and K values are indicated above each structure plot.

MOLECULAR ECOLOGY

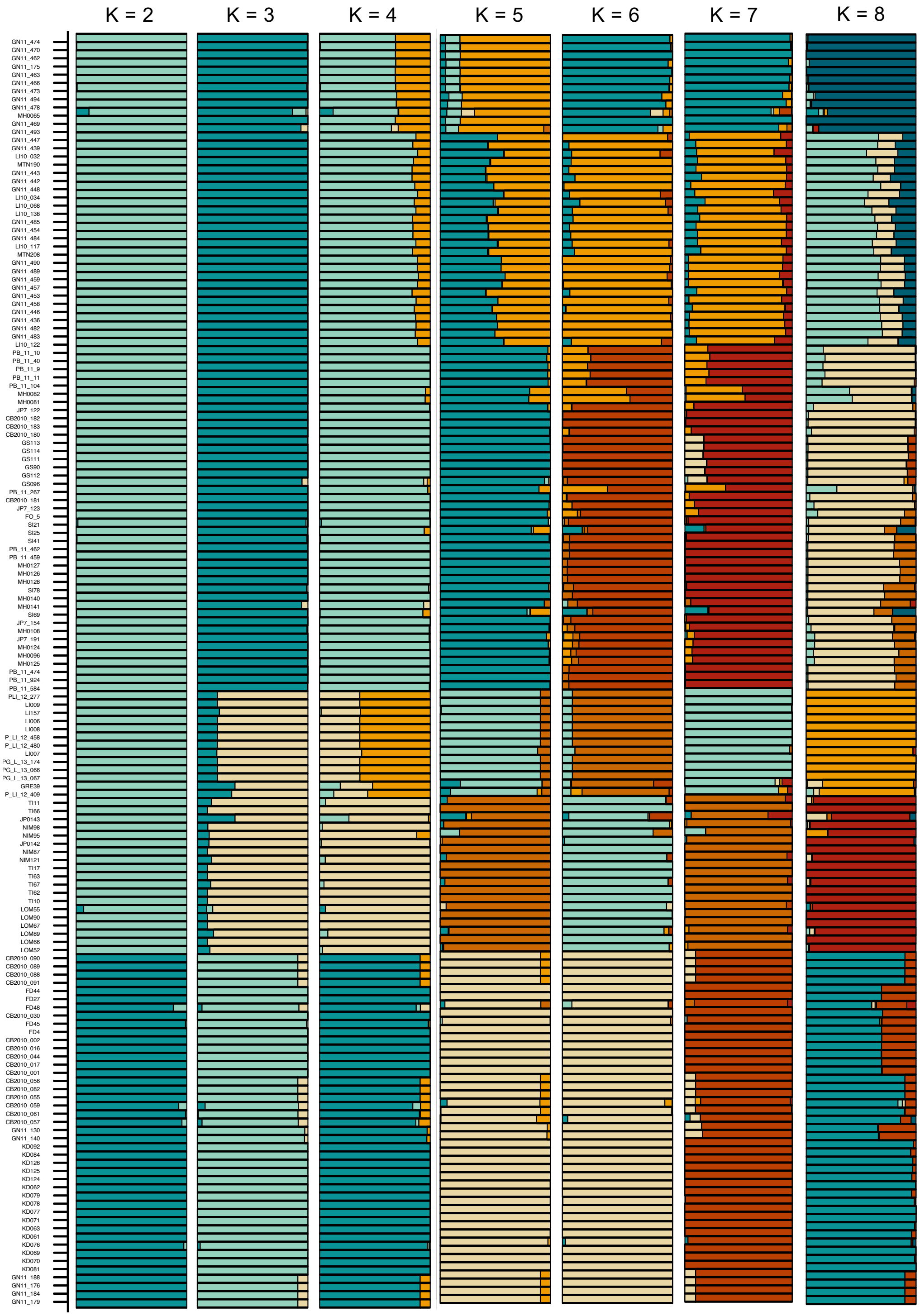

Figure S12. Structure plots for the joint *C. kamancamarai* and *C. alleni* dataset displaying genetic clusters across K-values ranging from 2 to 8. Horizontal bars represent single individuals and admixture proportions are coloured according to cluster assignment. Sample IDs are listed in the left column. K values are indicated in the top row.

MOLECULAR ECOLOGY

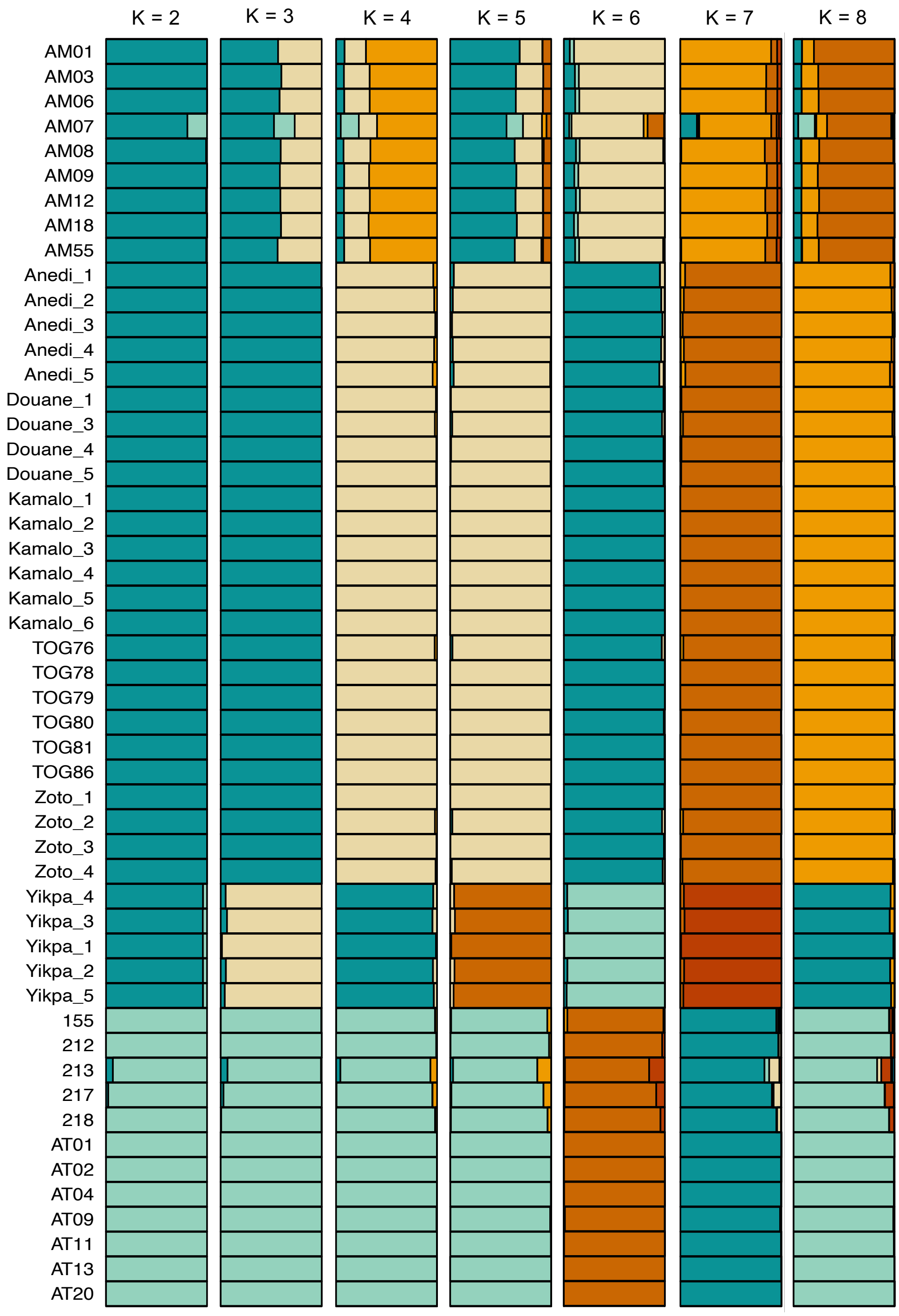

Figure S13. Structure plots for the joint *C. sagyimase* and *C. derooi* dataset displaying genetic clusters across K-values ranging from 2 to 8. Horizontal bars represent single individuals and admixture proportions are coloured according to cluster assignment. Sample IDs are listed in the left column. K values are indicated in the top row.

MOLECULAR ECOLOGY

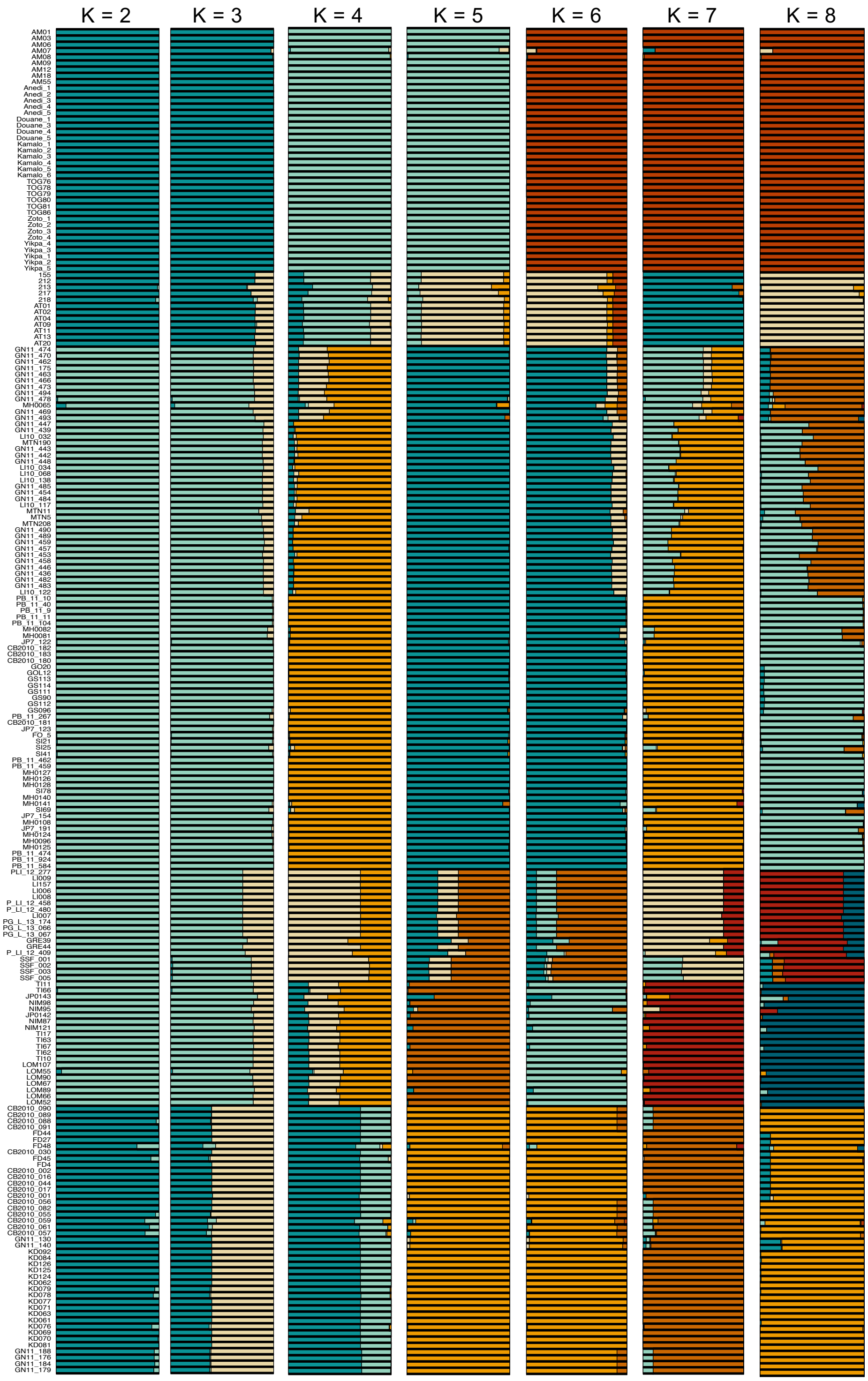

Figure S14. Structure plots for the full dataset (including samples from all species) displaying genetic clusters across K-values ranging from 2 to 8. Horizontal bars represent single individuals and admixture proportions are coloured according to cluster assignment. Sample IDs are listed in the left column. K values are indicated in the top row.

MOLECULAR ECOLOGY

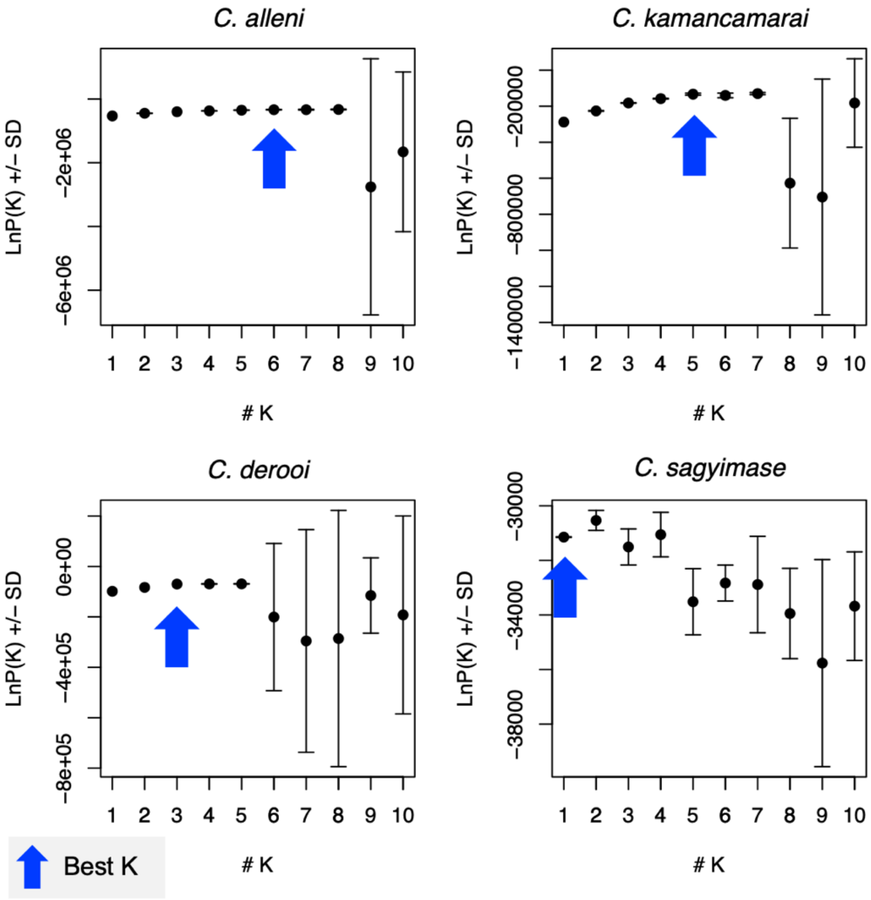

Figure S15. Mean Log Likelihood values across K values obtained with STRUCTURE for a) *C. alleni*, b) *C. kamancamarai*, c) *C. derooi*, and d) *C. sagyimase*. The Y-axis shows mean log likelihood (LnP(K)) values from 10 independent runs. The X-axis shows K values ranging from 1 to 10. Error bars indicate the standard deviation across runs. Runs at larger K values did not converge and due to large error bars, the variation at lower K values is not easily readable. We therefore excluded them in Fig. S16. Blue arrows show the selected optimal K chosen based on multiple criteria (PCA, STRUCTURE plots and their respective likelihood, as well as the phylogenetic support).

MOLECULAR ECOLOGY

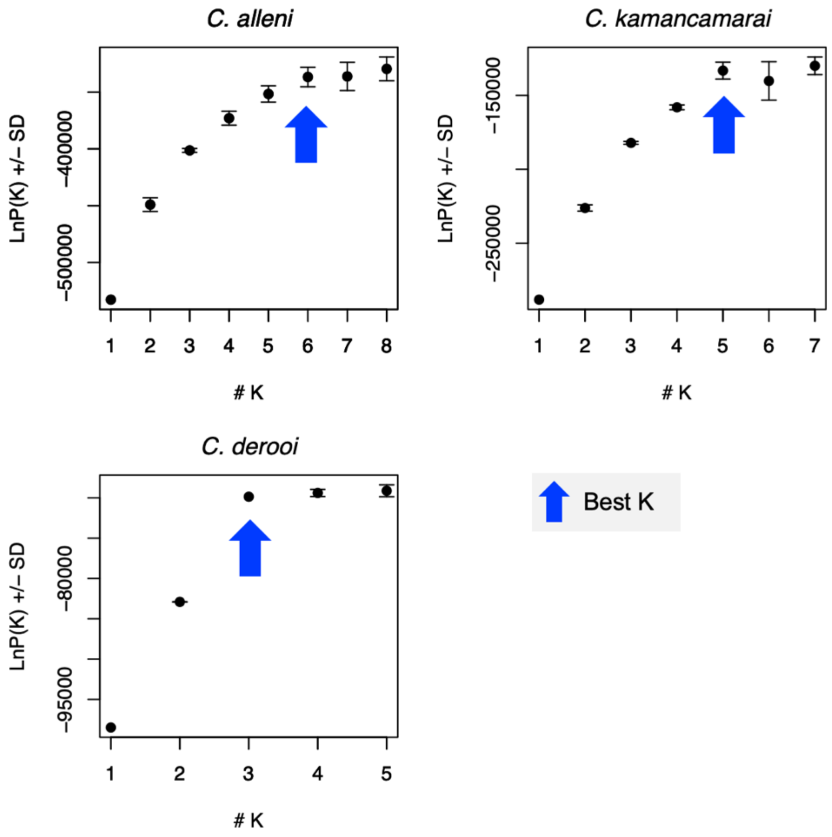

Figure S16. Mean Log Likelihood values across K values obtained with STRUCTURE for a) *C. alleni*, b) *C. kamancamarai*, c) *C. derooi*. This plot excludes higher K values that exhibited large standard deviations in LnP(K). Blue arrows show the selected optimal K chosen based on multiple criteria (PCA, STRUCTURE plots and their respective likelihood, as well as the phylogenies).

MOLECULAR ECOLOGY

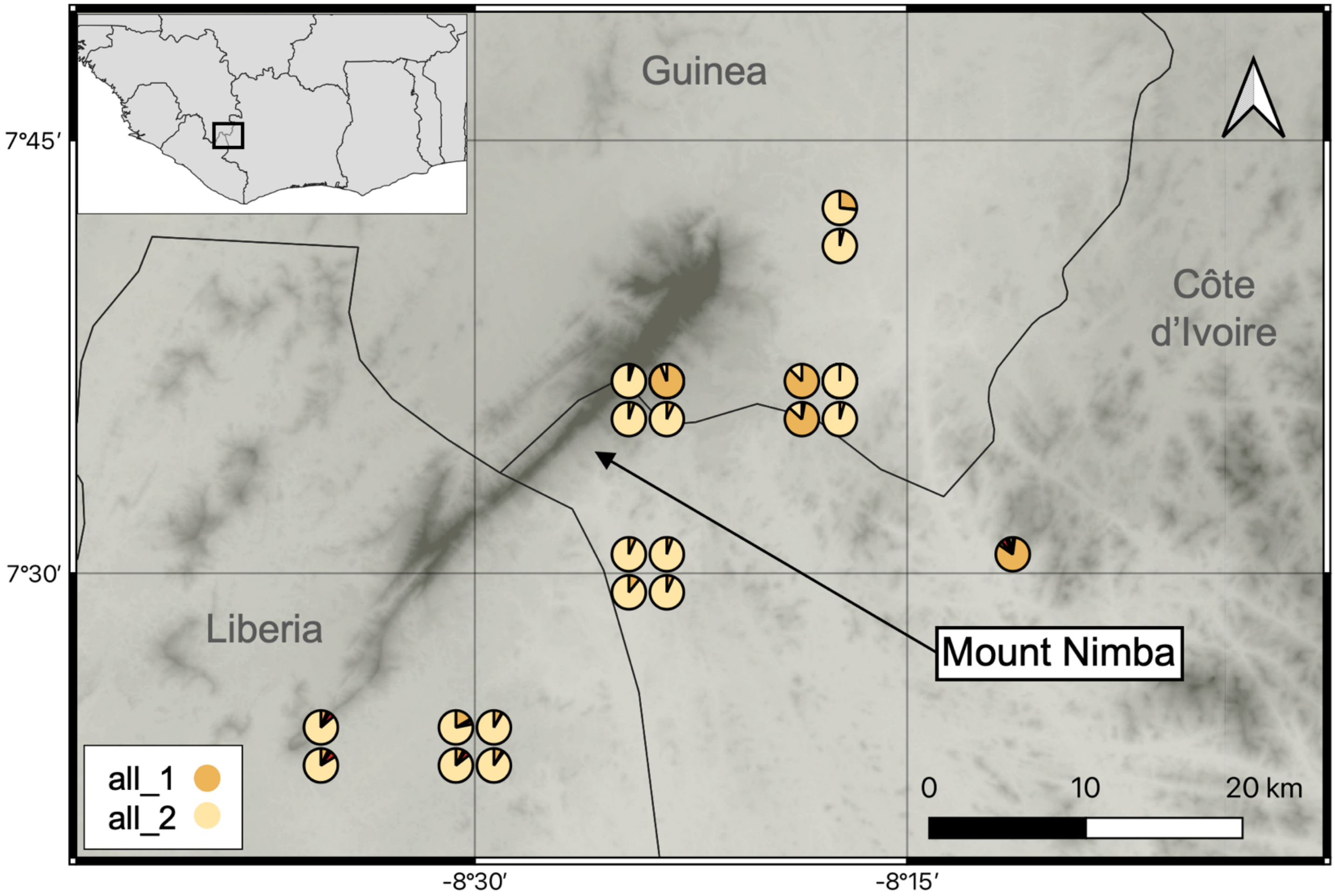

Figure S17. Zoomed-in view of spatial genetic patterns in the Mount Nimba region. Pie charts represent admixture proportions for clusters All_1 and All_2 at K = 6 (colour legend on the bottom left). Shading indicates elevation, with darker grey tones representing higher altitudes. A north arrow (top right), scale bar (bottom right), and locator map (top left) are shown.

MOLECULAR ECOLOGY

All_1

Model	AIC	Parameter values
Neutral	210.8	**Ne1**: 1
2-epoch	94.0	**Ne1**: 1, **Ne2**: 3.209, **T**: 0.110
**3-epoch**	**87.06**	**NeB**: 0.0107, **NeF**: 0.7941, **TB**: 0.0129, **TF**: 0.115

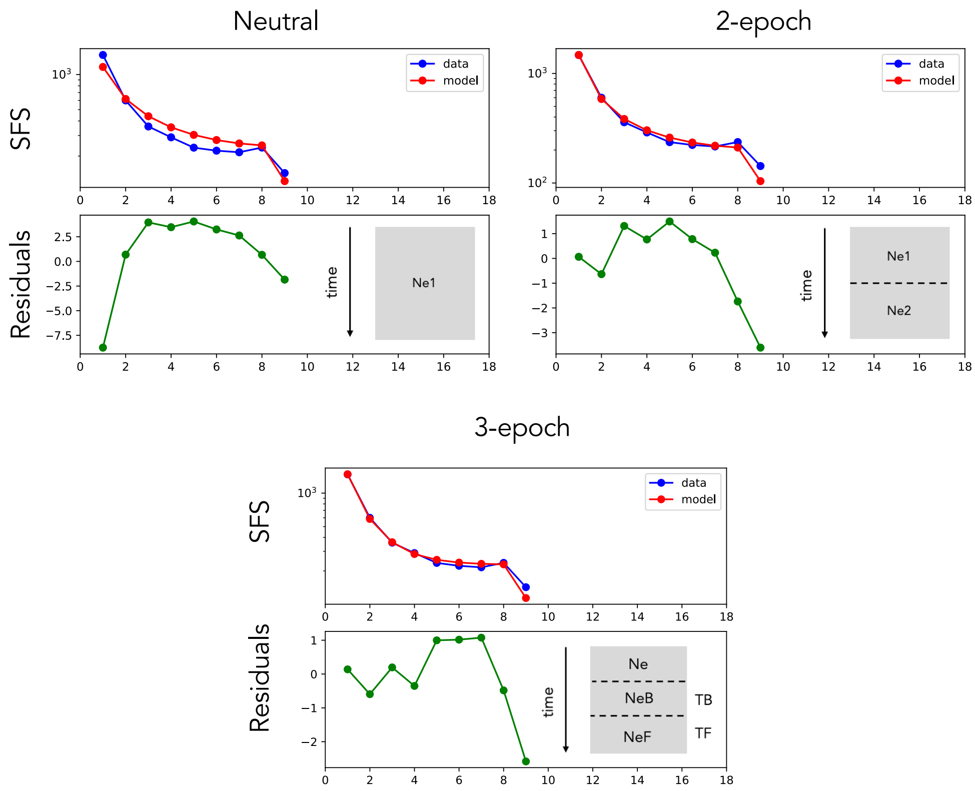

Figure S18. Results of 1D demographic modelling examining population size changes for population All_1. The top table summarizes AIC values and optimized parameter estimates for the neutral model, the 2-epoch, and the 3-epoch model allowing for instantaneous population size change at any given time. To assess model fit visually, each model's plot includes a site frequency spectrum (SFS) comparing observed data (blue line) to modelled data (red line) and a residual plot. Schematic representations of the demographic models are shown in the lower right corner of each residual plot.

MOLECULAR ECOLOGY

All_2	Model	AIC	Parameter values
	Neutral	149.0	**Ne1**: 1
	**2-epoch**	**136.2**	**Ne1**: 1, **Ne2**: 0.346, **T**: 0.840
	3-epoch	140.16	**NeB**: 6.9507, **NeF**: 0.8675, **TB**: 6.3591, **TF**: 2.8098

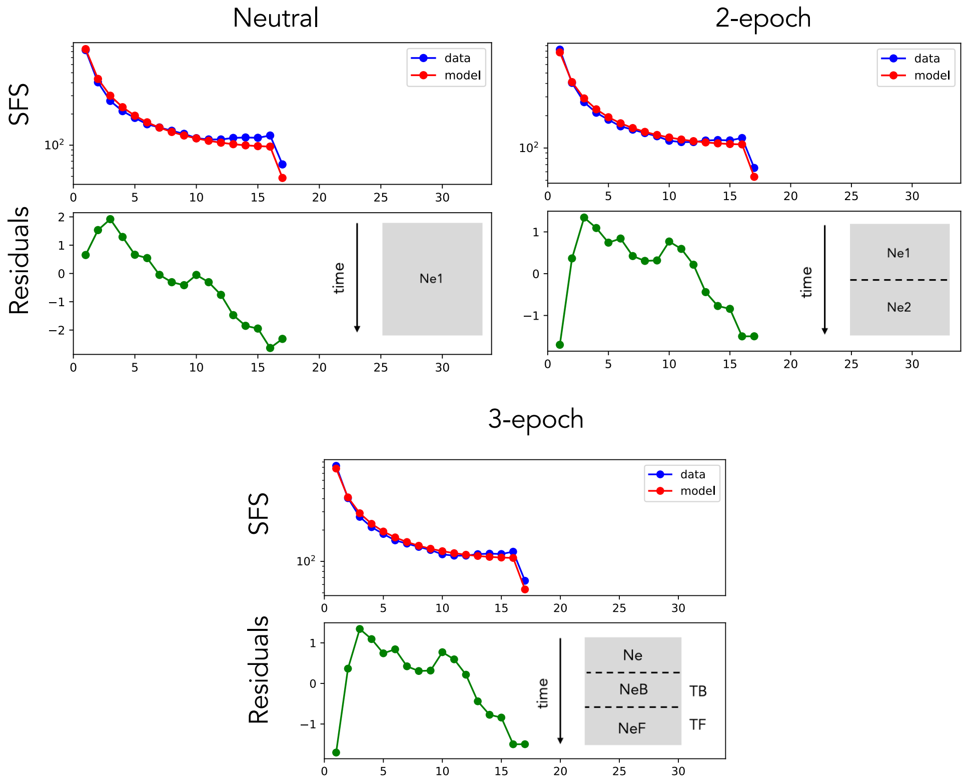

Figure S19. Results of 1D demographic modelling examining population size changes for population All_2. The top table summarizes AIC values and optimized parameter estimates for the neutral model, and the 2-epoch and the 3-epoch model allowing for instantaneous population size change at any given time. To assess model fit visually, each model's plot includes a site frequency spectrum (SFS) comparing observed data (blue line) to modelled data (red line) and a residual plot. Schematic representations of the demographic models are shown in the lower right corner of each residual plot.

MOLECULAR ECOLOGY

All_3

Model	AIC	Parameter values
Neutral	500.0	**Ne1**: 1
**2-epoch**	**164.9**	**Ne1**: 1, **Ne2**: 2.248, **T**: 0.304
3-epoch	167.6	**NeB**: 1.8627, **NeF**: 2.6207, **TB**: 0.3357, **TF**: 0.0779

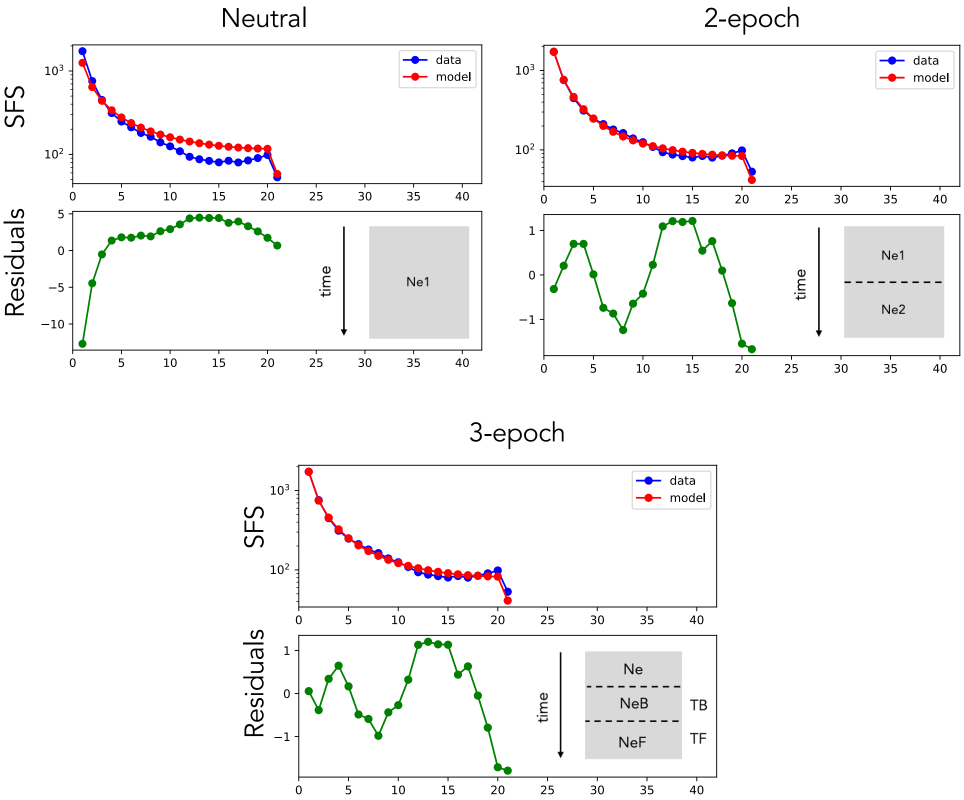

Figure S20. Results of 1D demographic modelling examining population size changes for population All_3. The top table summarizes AIC values and optimized parameter estimates for the neutral, the 2-epoch, and the 3-epoch model allowing for instantaneous population size change at any given time. To assess model fit visually, each model's plot includes a site frequency spectrum (SFS) comparing observed data (blue line) to modelled data (red line) and a residual plot. Schematic representations of the demographic models are shown in the lower right corner of each residual plot.

MOLECULAR ECOLOGY

All_4	Model	AIC	Parameter values
	Neutral	211.6	**Ne1**: 1
	2-epoch	96.6	**Ne1**: 1, **Ne2**: 5.762, **T**: 0.119
	**3-epoch**	**88.52**	**NeB**: 0.01, **NeF**: 1.0615, **TB**: 0.0126, **TF**: 0.1008

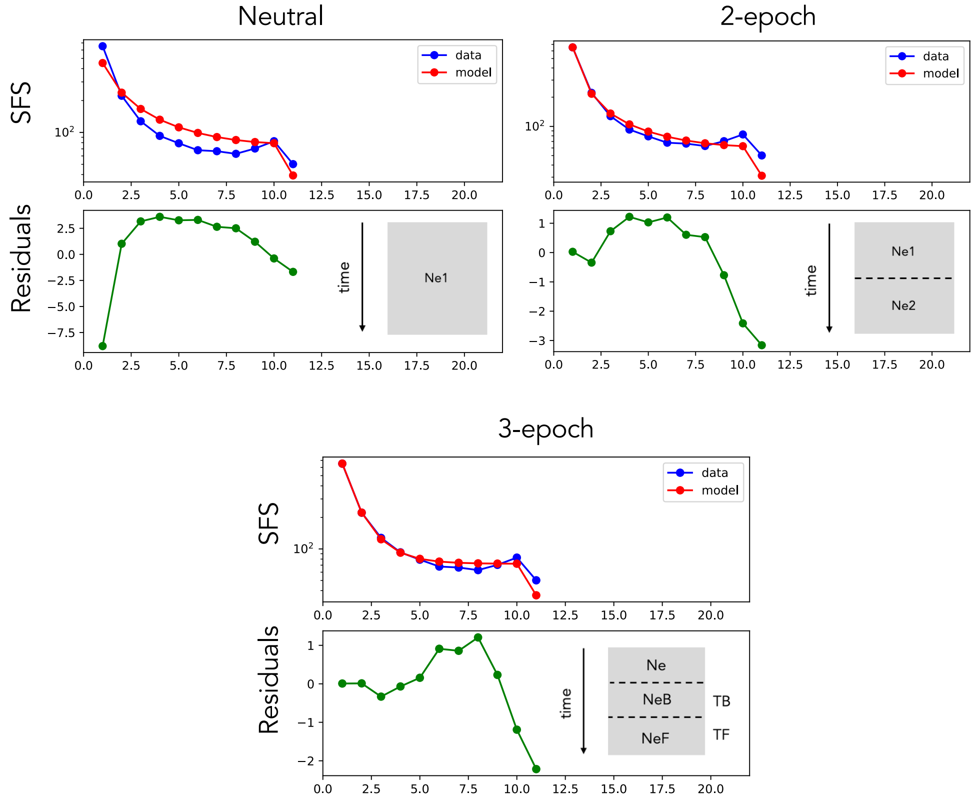

Figure S21. Results of 1D demographic modelling examining population size changes for population All_4. The top table summarizes AIC values and optimized parameter estimates for the neutral, the 2-epoch, and the 3-epoch model allowing for instantaneous population size change at any given time. To assess model fit visually, each model's plot includes a site frequency spectrum (SFS) comparing observed data (blue line) to modelled data (red line) and a residual plot. Schematic representations of the demographic models are shown in the lower right corner of each residual plot.

MOLECULAR ECOLOGY

All_5	Model	AIC	Parameter values
	Neutral	568.7	**Ne1**: 1
	**2-epoch**	**126.5**	**Ne1**: 1, **Ne2**: 2.822, **T**: 0.356
	3-epoch	126.8	**NeB**: 0.0106, **NeF**: 0.898, **TB**: 0.0135, **TF**: 0.1883

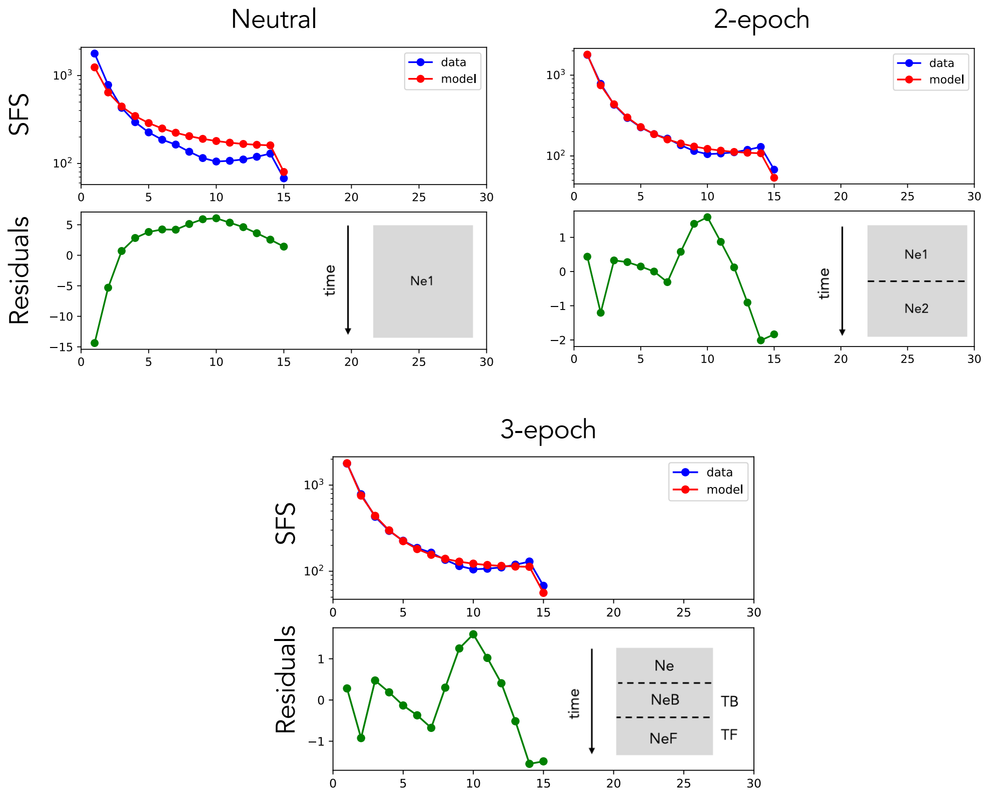

Figure S22. Results of 1D demographic modelling examining population size changes for population All_5. The top table summarizes AIC values and optimized parameter estimates for the neutral, the 2-epoch, and the 3-epoch model allowing for instantaneous population size change at any given time. To assess model fit visually, each model's plot includes a site frequency spectrum (SFS) comparing observed data (blue line) to modelled data (red line) and a residual plot. Schematic representations of the demographic models are shown in the lower right corner of each residual plot.

MOLECULAR ECOLOGY

Kam_2	Model	AIC	Parameter values
	Neutral	65.2	**Ne1**: 1
	**2-epoch**	**55.4**	**Ne1**: 1, **Ne2**: 1.943, **T**: 0.069
	3-epoch	57.5	**NeB**: 0.011, **NeF**: 0.6943, **TB**: 0.01, **TF**: 0.1597

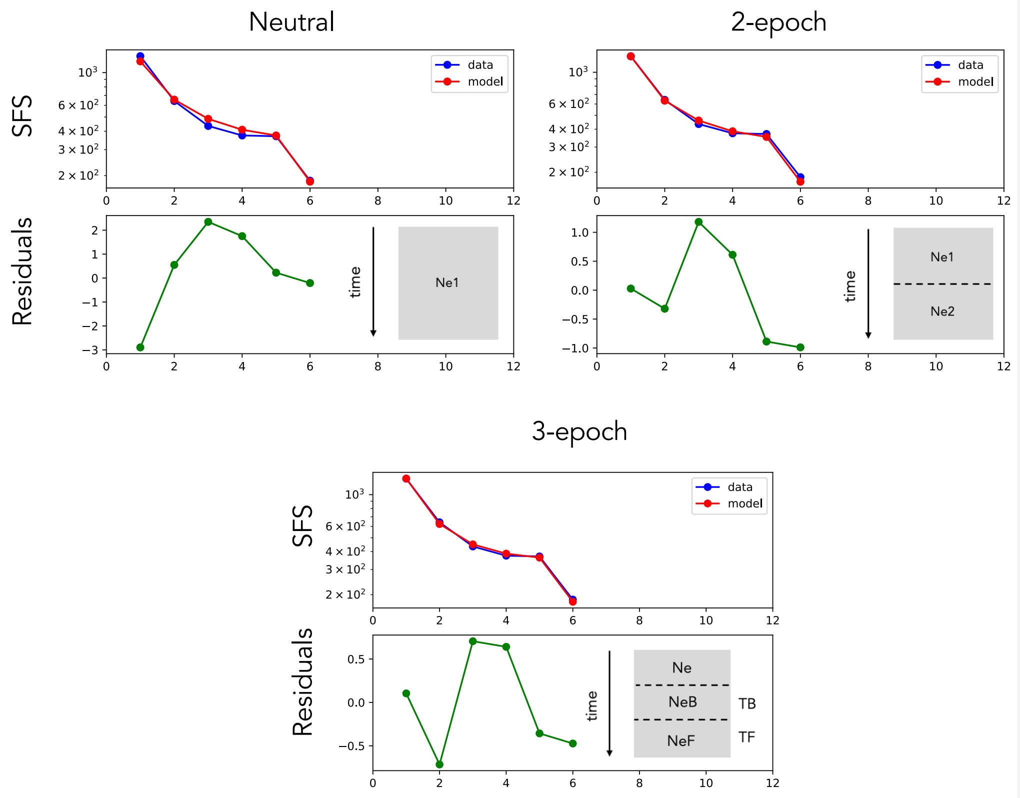

Figure S23. Results of 1D demographic modelling examining population size changes for population Kam_2. The top table summarizes AIC values and optimized parameter estimates for the neutral, the 2-epoch, and the 3-epoch model allowing for instantaneous population size change at any given time. To assess model fit visually, each model's plot includes a site frequency spectrum (SFS) comparing observed data (blue line) to modelled data (red line) and a residual plot. Schematic representations of the demographic models are shown in the lower right corner of each residual plot.

MOLECULAR ECOLOGY

Kam_5

Model	AIC	Parameter values
Neutral	118.3	**Ne1**: 1
**2-epoch**	**93.6**	**Ne1**: 1, **Ne2**: 0.376, **T**: 0.741
3-epoch	97.4	**NeB**: 0.142, **NeF**: 0.394, **TB**: 0.1144, **TF**: 0.1975

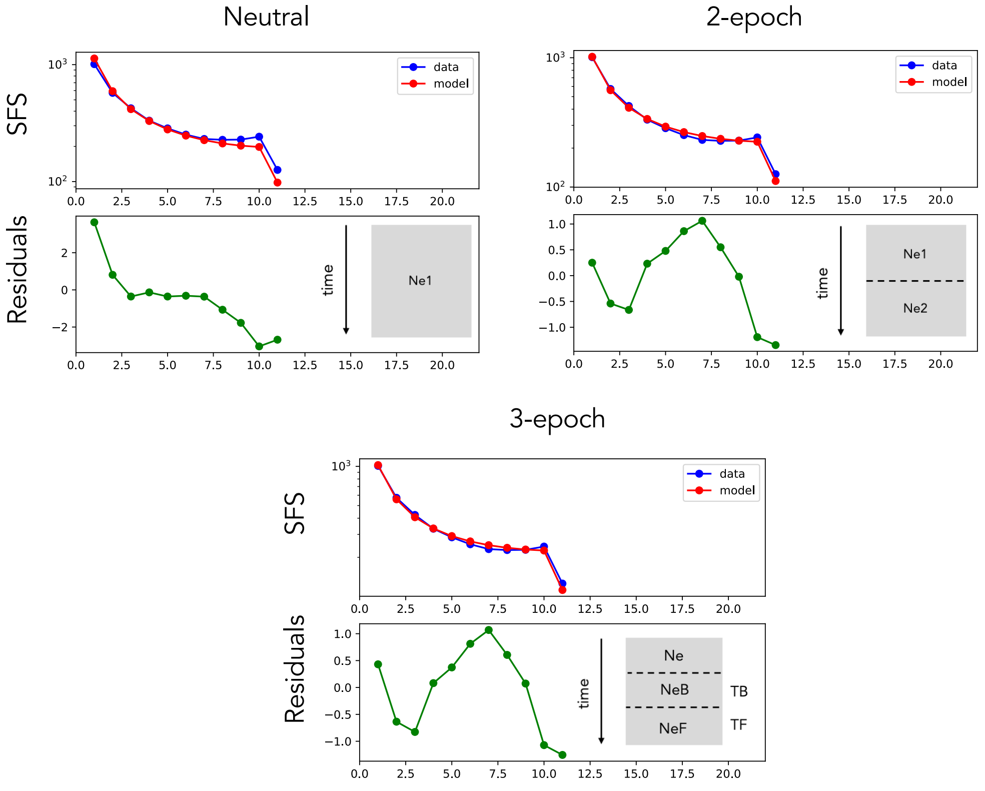

Figure S24. Results of 1D demographic modelling examining population size changes for population Kam_5. The top table summarizes AIC values and optimized parameter estimates for the neutral, the 2-epoch, and the 3-epoch model allowing for instantaneous population size change at any given time. To assess model fit visually, each model's plot includes a site frequency spectrum (SFS) comparing observed data (blue line) to modelled data (red line) and a residual plot. Schematic representations of the demographic models are shown in the lower right corner of each residual plot.

MOLECULAR ECOLOGY

Sag_1

Model	AIC	Parameter values
Neutral	154.3	**Ne1**: 1
2-epoch	101.4	**Ne1**: 1, **Ne2**: 2.241, **T**: 0.095
**3-epoch**	**94.16**	**NeB**: 0.01, **NeF**: 0.6048, **TB**: 0.0126, **TF**: 0.1049

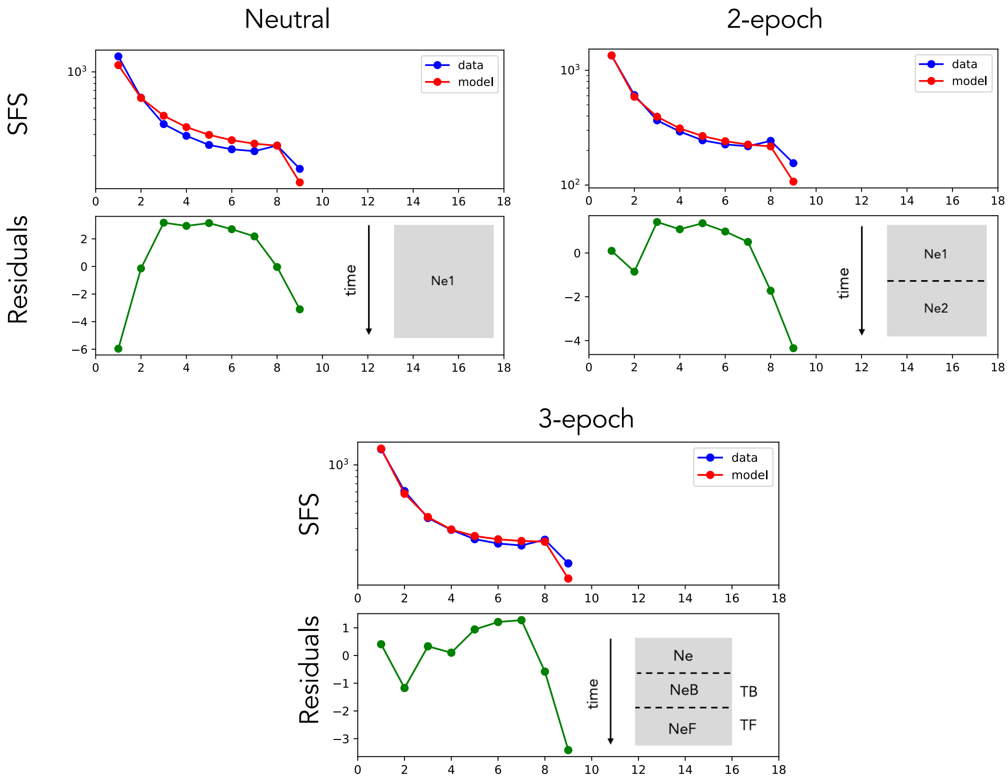

Figure S25. Results of 1D demographic modelling examining population size changes for population Sag_1. The top table summarizes AIC values and optimized parameter estimates for the neutral, the 2-epoch, and the 3-epoch model allowing for instantaneous population size change at any given time. To assess model fit visually, each model's plot includes a site frequency spectrum (SFS) comparing observed data (blue line) to modelled data (red line) and a residual plot. Schematic representations of the demographic models are shown in the lower right corner of each residual plot.

MOLECULAR ECOLOGY

Der_2

Model	AIC	Parameter values
Neutral	212.6	**Ne1**: 1
2-epoch	201.14	**Ne1**: 1, **Ne2**: 0.177, **T**: 0.547
**3-epoch**	**168.5**	**NeB**: 0.0101, **NeF**: 0.429, **TB**: 0.0109, **TF**: 0.0594

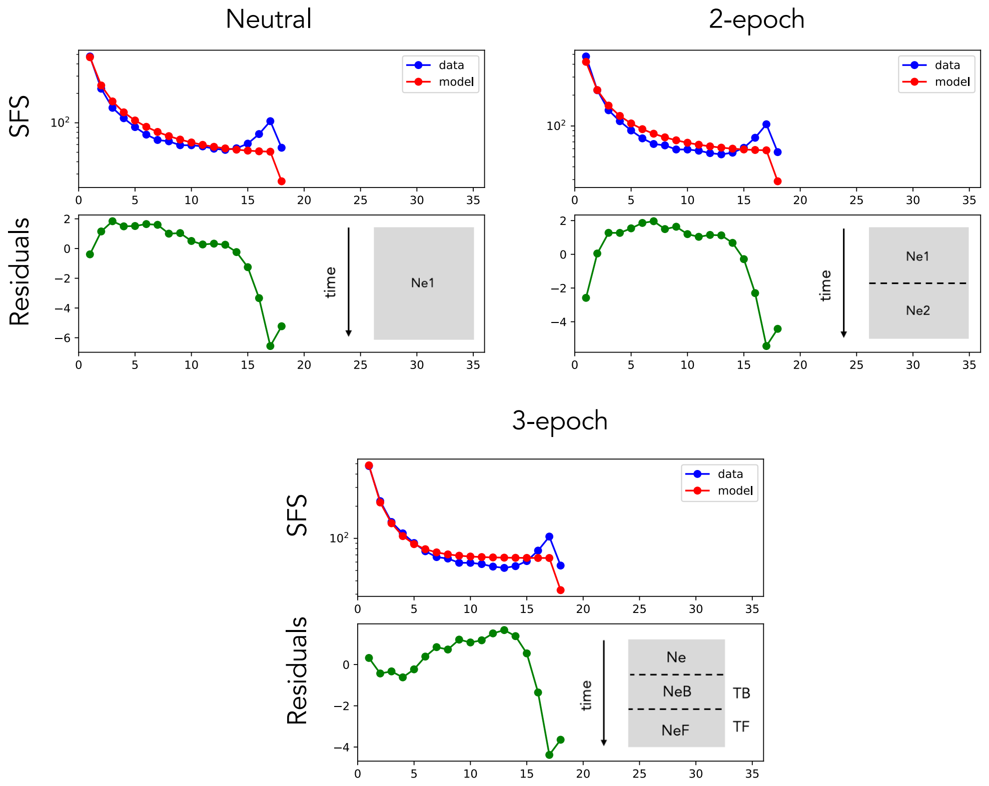

Figure S26. Results of 1D demographic modelling examining population size changes for population Der_2. The top table summarizes AIC values and optimized parameter estimates for the neutral model and the 2-epoch and the 3-epoch model allowing for instantaneous population size change at any given time. To assess model fit visually, each model's plot includes a site frequency spectrum (SFS) comparing observed data (blue line) to modelled data (red line) and a residual plot. Schematic representations of the demographic models are shown in the lower right corner of each residual plot.

All_1 vs All_2

Model	AIC
No_mig	2531.84
Sym_mig	1606.24
Sec_contact	1561.9
No_mig_size	2554.48
**Sym_mig_size**	**1448.06**
Sec_contact_size	1612.28

Parameter	Estimate
nu1a	0.5108
nu2a	0.8499
nu1b	4.9421
nu2b	1.2527
m	0.4433
T1	17.8584
T2	0.0663

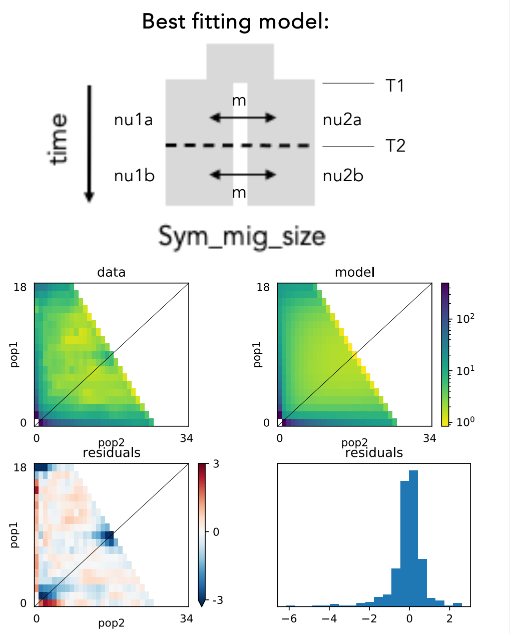

Figure S27. Results of 2D demographic modelling examining population size and connectivity over time for populations All_1 and All_2. The top-left table shows the AIC values for each model (best-fitting model in bold). The table in the lower left shows the optimized parameter values for the best model. A schematic representation of the best fitting demographic model is fount to the top right. The plots at the bottom right show: the site frequency spectrum (SFS) of the observed data (left) and modelled data (right) as well as the residual plots.

All_1 vs All_3

Model	AIC
No_mig	2986.9
Sym_mig	1892.32
**Sec_contact**	**1682.48**
No_mig_size	2942.42
Sym_mig_size	1810.38
Sec_contact_size	1816.0

Parameter	Estimate
nu1	0.561
nu2	1.3327
m	0.981
T1	0.7383
T2	0.0317

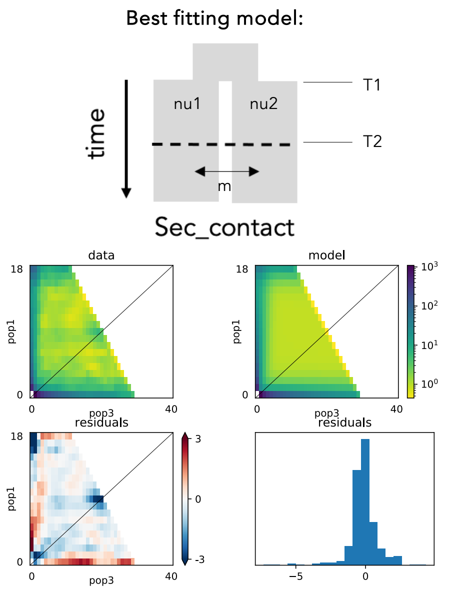

Figure S28. Results of 2D demographic modelling examining population size and connectivity over time for populations All_1 and All_3. The top-left table shows the AIC values for each model (best-fitting model in bold). The table in the lower left shows the optimized parameter values for the best model. A schematic representation of the best fitting demographic model is fount to the top right. The plots at the bottom right show: the site frequency spectrum (SFS) of the observed data (left) and modelled data (right) as well as the residual plots.

All_1 vs All_4

Model	AIC
No_mig	1285.98
Sym_mig	921.3
Sec_contact	912.04
No_mig_size	1076.7
Sym_mig_size	928.64
**Sec_contact_size**	**777.08**

Parameter	Estimate
nu1a	0.1084
nu2a	0.0817
nu1b	1.1028
nu2b	1.3133
m	0.2455
T1	0.1279
T2	0.0521

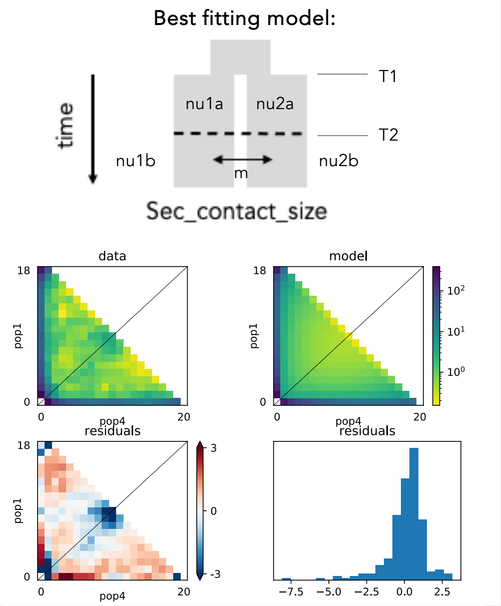

Figure S29. Results of 2D demographic modelling examining population size and connectivity over time for populations All_1 and All_4. The top-left table shows the AIC values for each model (best-fitting model in bold). The table in the lower left shows the optimized parameter values for the best model. A schematic representation of the best fitting demographic model is fount to the top right. The plots at the bottom right show: the site frequency spectrum (SFS) of the observed data (left) and modelled data (right) as well as the residual plots.

All_2 vs All_3

Model	AIC
No_mig	2857.28
Sym_mig	2276.38
**Sec_contact**	**1937.54**
No_mig_size	2863.58
Sym_mig_size	2273.8
Sec_contact_size	2204.48

Parameter	Estimate
nu1	4.7186
nu2	11.0087
m	0.104
T1	4.6283
T2	9.8704

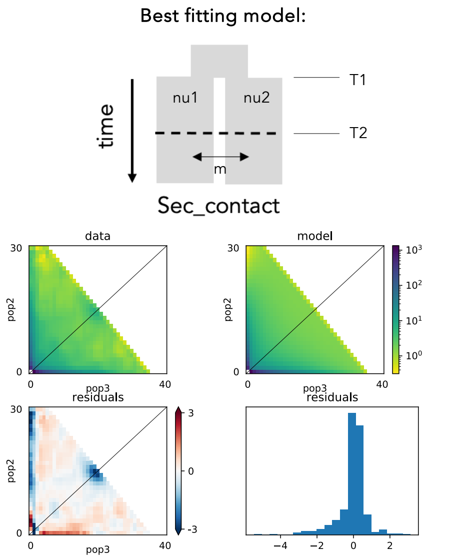

Figure S30. Results of 2D demographic modelling examining population size and connectivity over time for populations All_2 and All_3. The top-left table shows the AIC values for each model (best-fitting model in bold). The table in the lower left shows the optimized parameter values for the best model. A schematic representation of the best fitting demographic model is fount to the top right. The plots at the bottom right show: the site frequency spectrum (SFS) of the observed data (left) and modelled data (right) as well as the residual plots.

MOLECULAR ECOLOGY

All_3 vs All_5

Model	AIC
No_mig	2736.94
Sym_mig	1901.38
Sec_contact	1796.48
No_mig_size	2517.8
Sym_mig_size	1695.8
**Sec_contact_size**	**1662.74**

Parameter	Estimate
nu1a	1.1225
nu2a	0.8341
nu1b	1.5132
nu2b	2.389
m	0.2011
T1	0.9838
T2	0.1713

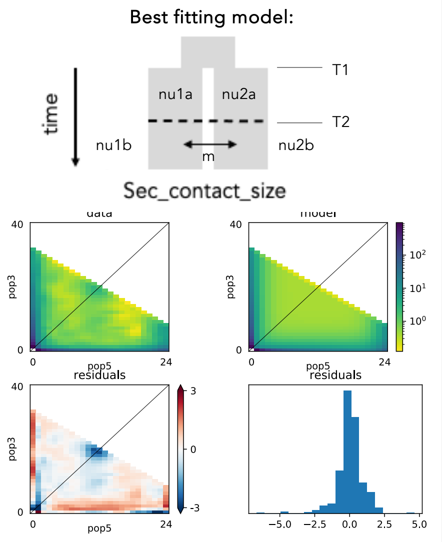

Figure S31. Results of 2D demographic modelling examining population size and connectivity over time for populations All_3 and All_5. The top-left table shows the AIC values for each model (best-fitting model in bold). The table in the lower left shows the optimized parameter values for the best model. A schematic representation of the best fitting demographic model is fount to the top right. The plots at the bottom right show: the site frequency spectrum (SFS) of the observed data (left) and modelled data (right) as well as the residual plots.

Kam_2 vs Kam_5

Model	AIC
No_mig	1296.02
Sym_mig	782.28
Sec_contact	782.16
No_mig_size	875.16
**Sym_mig_size**	**767.16**
Sec_contact_size	787.0

Parameter	Estimate
nu1a	0.4568
nu2a	0.6255
nu1b	0.877
nu2b	0.5406
m	0.354
T1	2.9777
T2	0.1527

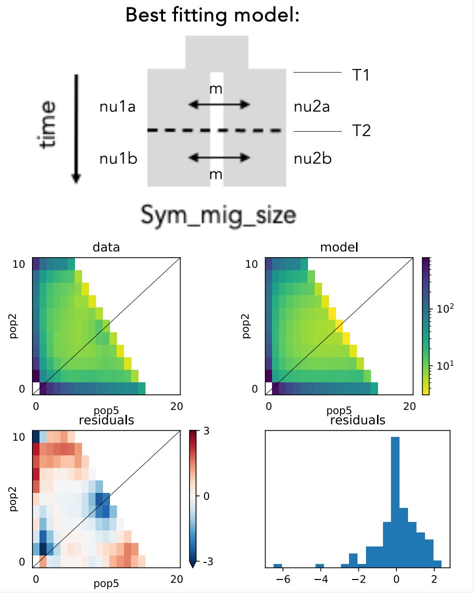

Figure S32. Results of 2D demographic modelling examining population size and connectivity over time for populations Kam_2 and Kam_5. The top-left table shows the AIC values for each model (best-fitting model in bold). The table in the lower left shows the optimized parameter values for the best model. A schematic representation of the best fitting demographic model is fount to the top right. The plots at the bottom right show: the site frequency spectrum (SFS) of the observed data (left) and modelled data (right) as well as the residual plots.
